# Phosphorus Centers of Different Hybridization in Phosphaalkene-Substituted Phospholes

**DOI:** 10.1002/chem.201402406

**Published:** 2014-05-30

**Authors:** Elisabet Öberg, Andreas Orthaber, Christophe Lescop, Régis Réau, Muriel Hissler, Sascha Ott

**Affiliations:** [a] Department of Chemistry, Ångström Laboratories, Uppsala UniversityBox 523, 75120 Uppsala (Sweden), Fax: (+46) 18-471-6844 E-mail: sascha.ott@kemi.uu.se; [b] Institut des Sciences Chimiques de Rennes, UMR6226, CNRS-Université de Rennes 1Campus de Beaulieu, 35042 Rennes Cedex (France) E-mail: muriel.hissler@univ-rennes1.frregis.reau@univ-rennes1.fr

**Keywords:** conjugation, electronic structure, phosphaalkenes, phosphorus, X-ray diffraction

## Abstract

Phosphole-substituted phosphaalkenes (PPAs) of the general formula Mes*P=C(CH_3_)–(C_4_H_2_P(Ph))–R **5 a**–**c** (Mes*=2,4,6-*t*Bu_3_Ph; R=2-pyridyl (**a**), 2-thienyl (**b**), phenyl (**c**)) have been prepared from octa-1,7-diyne-substituted phosphaalkenes by utilizing the Fagan–Nugent route. The presence of two differently hybridized phosphorus centers (σ^2^,λ^3^ and σ^3^,λ^3^) in **5** offers the possibility to selectively tune the HOMO–LUMO gap of the compounds by utilizing the different reactivity of the two phosphorus heteroatoms. Oxidation of **5 a**–**c** by sulfur proceeds exclusively at the σ^3^,λ^3^-phosphorus atom, thus giving rise to the corresponding thioxophospholes **6 a**–**c**. Similarly, **5 a** is selectively coordinated by AuCl at the σ^3^,λ^3^-phosphorus atom. Subsequent second AuCl coordination at the σ^2^,λ^3^-phosphorus heteroatom results in a dimetallic species that is characterized by a gold–gold interaction that provokes a change in π conjugation. Spectroscopic, electrochemical, and theoretical investigations show that the phosphaalkene and the phosphole both have a sizable impact on the electronic properties of the compounds. The presence of the phosphaalkene unit induces a decrease of the HOMO–LUMO gap relative to reference phosphole-containing π systems that lack a P=C substituent.

## Introduction

The field of organic π-conjugated materials has matured tremendously over the last two decades.[1] More and more materials, which range from single molecules to oligomers and polymers, have found use in organic electronics devices, for example, and a plethora of organic compounds with potential applications within the field of molecular electronics have been synthesized.[2] Today, the art of organic synthesis is well developed, and both the chemical and electronic properties of synthesized organic compounds are easily modified.[3] In particular, the insertion of heteroatoms in the backbone of π systems has attracted great interest owing to the impact of the heteroatom on the electronic properties of the molecules.[4] Furthermore, postsynthetic manipulations such as metal coordination or oxidation reactions can be performed to modify the physical properties of the π system.[1c, 5] Recently, it has been shown that the inclusion of phosphaalkenes (a σ^2^,λ^3^-phosphorus center) into oligoacetylenes has a pronounced effect on the energies of the frontier molecular orbitals.[6] Similar effects have been described for oligomeric and polymeric π-conjugated systems in which σ^2^,λ^3^-phosphorus centers feature in the form of phosphaalkenes or diphosphenes.[7] A specific class of phosphorus-containing π-conjugated materials are phospholes, that is, phosphorus analogues to pyrroles.[5] The tricoordinated phosphorus atom of phospholes possesses a pyramidal geometry with a lone pair of pronounced s character. These geometric and electronic features prevent an efficient endocyclic conjugation of the electron sextet. In fact, delocalization within the phosphole ring arises from a hyperconjugation that involves the exocyclic P–R σ bond and the π system of the diene moiety.[8] As a consequence, the phosphole ring exhibits a unique set of properties (low aromatic character, reactive P atom, σ–π hyperconjugation) that has motivated the development of new conjugated π systems that integrate the phosphole unit.[9] In particular, the intact reactivity of the phosphorus heteroatom has proven to provide a unique possibility for facile tuning of the optoelectronic properties of phospholes (e.g., through oxidation or metal coordination to the phosphorus), thereby making them suitable for applications within optoelectronic devices.[5b, 9a, 10] In an early study, Mathey and co-workers combined two differently hybridized P centers, namely, a phosphole and a phosphinine unit[11] or a phosphole decorated with an imine functionality,[12] and explored their coordination behavior. In the present work, we wish to chemically tune the electronic properties of a new class of compounds that contains two differently hybridized phosphorus centers in the form of phosphaalkenes and phospholes as integral parts of a conjugated system. Through the unique reactivity of two differently hybridized phosphorus atoms, chemical manipulations will offer possibilities to selectively alter the optical and electronic properties of the novel π-conjugated molecules. Herein, we present an unprecedented synthetic approach towards phosphole-phosphaalkenes (PPAs). Showcases for the differences in reactivity of the two phosphorus heteroatoms through metal coordination and oxidation are presented.

## Results and Discussion

### Synthesis and X-ray crystallography of phosphole-phosphaalkenes

**Scheme 1:**
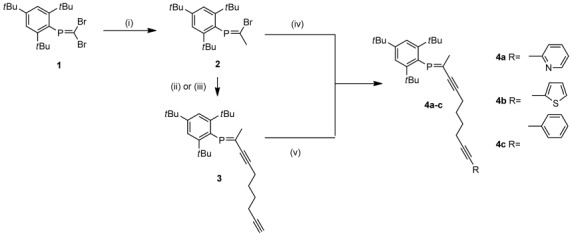
Synthesis of octadiyne-phosphaalkenes 4 a–c. i) 1) *n*BuLi, −130 °C, Trapp mixture, 30 min; 2) MeI, −120 °C, 30 min then to RT, 2 h. Compound 2 93 %. ii) 1,7-octadiynemagnesium bromide, [Pd(dba)_2_], PPh_3_, THF, reflux, 4–5 h. Compound 3 98 %. iii) 1,7-octadiyne, CuI, [Pd(PPh_3_)_2_Cl_2_], Et_2_NH, RT, 3 h. Compound 3 65 %. iv) 1-(2-pyridyl)octa-1,7-diyne, CuI, [Pd(PPh_3_)_2_Cl_2_], Et_2_NH, RT, 14 h. Compound 4 a 74 %. v) b) 2-iodothiophene, CuI, [Pd(PPh_3_)_2_Cl_2_], Et_3_N, RT, 14 h. Compound 4 b 96 %. c) iodobenzene, CuI, [Pd(PPh_3_)_2_Cl_2_], Et_2_NH, RT, 14 h. Compound 4 c 54 %.

Phosphole-substituted phosphaalkenes (PPAs) were targeted through a sequence of reactions in which the phosphole is formed in the last step from phosphaalkene-containing 1,7-octadiyne compounds **4 a**–**c** by using the Fagan–Nugent protocol (Scheme 1).[13] The strategy turned out to be a feasible synthetic route, as sterically protected phosphaalkenes are compatible with *n*BuLi and [Cp_2_ZrCl_2_] (Cp=cyclopentadienyl ligand), that is, the required reagents for the phosphole assembly. The synthetic sequence towards **4 a**–**c** starts from previously described *C*,*C*-dibromophosphaalkene **1** (Scheme 1).[14] For subsequent cross-coupling reactions, it is important to substitute one of the bromide atoms by other groups as palladium catalysts that are commonly used for cross-coupling reactions lead to a rearrangement of **1** and the formation of phosphaalkynes.[15] Using this strategy, Bickelhaupt et al. previously presented cross-coupling reactions on Mes*P=CHBr (Mes*=2,4,6-tri-*tert*-butylphenyl).[16] To increase the stabilities of the compounds, it was decided to introduce a methyl group instead of one bromide substituent in **1**. Hence, Mes*P=CBr_2_ (**1**) was treated with *n*BuLi at −120 °C in the Trapp mixture (THF/diethyl ether/pentane 4:4:1) for 30 min.[17] After quenching the reaction mixture with methyl iodide and subsequent workup, Mes*P=CCH_3_Br (**2**) was obtained in good yields.[18] The 1,7-octadiyne motif can be introduced by using one of two procedures that basically differ in the alkyne source. In the first case, **2** is coupled with 1,7-octadiynemagnesium bromide in THF by using [Pd(dba)_2_] (dba=dibenzylideneacetone) and PPh_3_ under reflux conditions.[16] This reaction gives octadiynephosphaalkene **3** in 98 % yield and is suitable for medium to large scales, preferably less than or equal to one gram, mainly due to the fact that it is easier to handle Grignard reagents on these scales. In accordance to the cross-couplings presented by Bickelhaupt et al., an isomerization occurs during the palladium-mediated coupling step, and only the *E* isomer of **3** is obtained from (*Z*)-**2** (see below).[16, 19]

**Figure 1:**
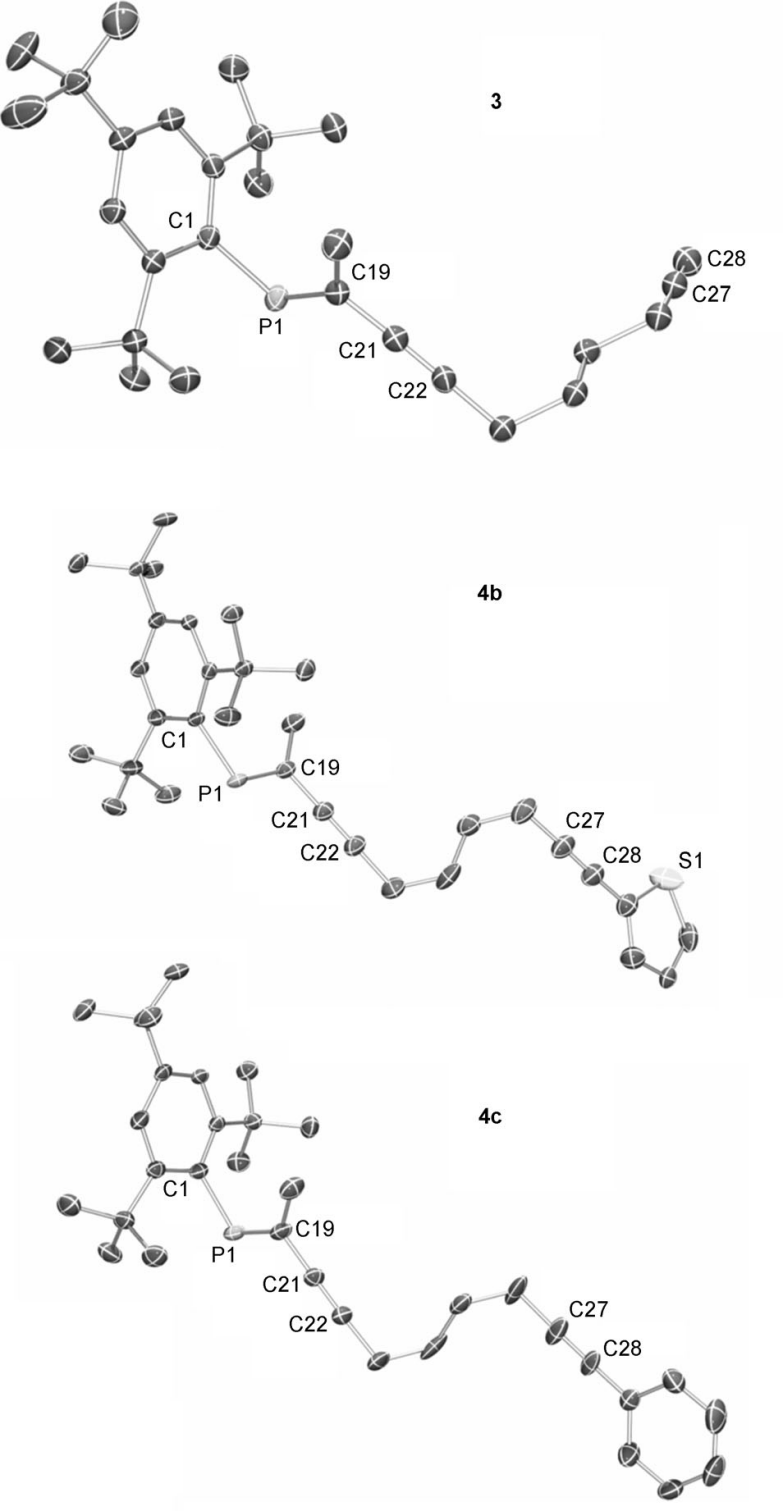
ORTEP plots of phosphaalkenes 3 and 4 b,c (ellipsoids are drawn at 50 % probability level). Hydrogen atoms and disorder in the alkyl groups are omitted for clarity. Selected bond lengths [Å]: Compound 3: P1–C1 1.855(2), P1=C19 1.693(2), C21≡C22 1.207(3), C27≡C28 1.194(4); compound 4 b: P1–C1, 1.852(3), P1=C19 1.686(3), C21≡C22 1.194(6), C27≡C28 1.183(6); Compound 4 c: P1–C1 1.8449(16), P1=C19 1.6834(18), C21≡C22 1.198(11), C27≡C28 1.185(10).

For smaller scales, **2** can be coupled directly with 1,7-octadiyne in Et_2_NH with [Pd(PPh_3_)_2_Cl_2_] and CuI as catalysts. The reaction time is crucial for this reaction, since longer reaction times result in homocoupling of the product. By using the standard Sonogashira coupling conditions, that is, amine as solvent and [Pd(PPh_3_)_2_Cl_2_] and CuI as catalysts in the presence of 2-bromopyridine, 2-iodothiophene, or iodobenzene, compound **3** can be converted into **4 a**–**c** in medium to good yields. Higher overall yields of **4 a** can be obtained by coupling **2** directly with 1-(2-pyridyl)octa-1,7-diyne. The direct cross-coupling approach also provides a larger functional group tolerance and would allow the introduction of substituents that are reactive towards Grignard reagents. Compounds **3** and **4 a**–**c** are isomerically pure, as is evident from one single ^31^P{^1^H} NMR spectroscopic resonance in the typical region for P=C compounds between *δ*=274.7 and 275.3 ppm. Figure 1 shows a depiction of the solid-state structures of **3** and **4 b**, **c**. The structures confirm the formation of *E* isomer **3** from *Z* isomer **2** during the preparation of the octadiynephosphaalkenes. In all cases, the bond lengths of the P=C double bonds and the C≡C triple bonds are in the usual range.

**Scheme 2:**
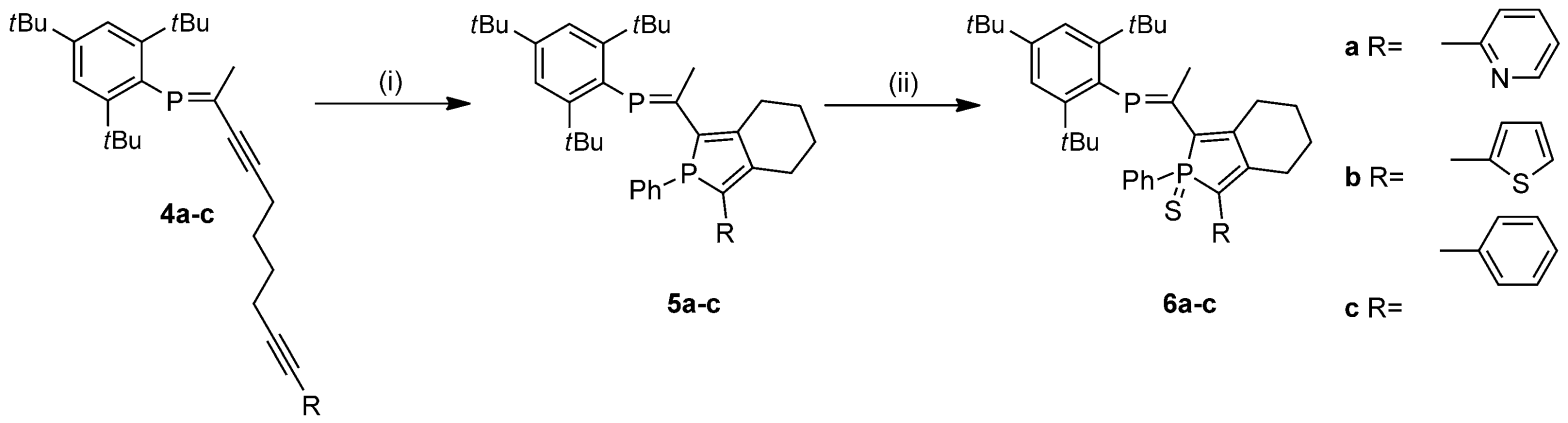
Synthesis of phosphole-phosphaalkenes (PPAs) 5,6 a–c. i) 1) [Cp_2_ZrCl_2_], *n*BuLi (2 equiv), THF, −78 °C 1 h, then RT, 14 h; 2) PhPBr_2_, −78 °C, then for 5 a; RT, 4 h, 5 b,c; RT, 24 h. Compound 5 a 42 %, 5 b 21 %, 5 c not isolated. ii) sulfur, CH_2_Cl_2_, RT, 14 h. Compound 6 a 40 %, 6 b 19 % (over 2 steps), 5 c 29 % (over 2 steps).

With phosphaalkene-substituted octadiynes in hand, PPAs were synthesized by utilizing the Fagan–Nugent route, which is a well-established method for the preparation of five-membered heterocycles.[13] Thus, treating phosphole precursors **4 a**–**c** with [Cp_2_ZrCl_2_] and *n*BuLi in THF results in the formation of zirconium metallacycle intermediates, which were subsequently treated with PhPBr_2_ to afford PPAs **5 a**–**c** (Scheme 2). Noteworthy is a difference in stability between the three PPAs. PPA **5 a** is stable under ambient conditions and on silica, thereby allowing purification by column chromatography without visible decomposition. In contrast, **5 b** was found to be less stable, and new red bands appeared during chromatographic purification, even when a rapid flash procedure was applied. The appearance of the red bands is consistent with partial oxidation of **5 b** during chromatographic workup. Compound **5 c** was not isolated and was directly subjected to oxidation with elemental sulfur (S_8_) (see below).

For a first indication of whether it would be possible to perform selective chemistry on either of the two phosphorus heteroatoms, PPAs **5 a**–**c** were subjected to oxidative conditions by using an excess amount of sulfur in CH_2_Cl_2_. Although such oxidation by sulfur is well established for phospholes[9, 10d, h] it has previously been shown that phosphaalkenes can also be oxidized by sulfur to thiaphosphiranes.[20] However, procedures for phosphaalkene oxidation usually suggest the addition of base to accelerate the reaction, although sulfurization is also described to proceed without a base but under extended reaction times.[20] It was thus an objective of this work to investigate whether selective oxidation of the phosphole in the series of PPAs is possible while maintaining the intactness of the phosphaalkene moiety. First indications of selective oxidation of the phospholes to a σ^4^,λ^5^ center can be anticipated from the ^31^P{^1^H} NMR spectra of the reaction products (Table 1). For PPAs **6 a**–**c**, the ^31^P{^1^H} NMR spectroscopic signals of the phosphole phosphorus centers are shifted downfield by approximately 40 ppm to approximately 52 ppm, which is in good accordance with the chemical shifts of other σ^4^,λ^5^-thioxophospholes.[9, 10d, h] Interestingly, also the ^31^P{^1^H} NMR spectroscopic resonances of the phosphaalkene phosphorus atoms are significantly shifted downfield by approximately 20 ppm. Nevertheless, the observed chemical shifts are still in the typical region for phosphaalkenes, which indicates that the phosphaalkene is left intact. Comparison with typical chemical shifts of oxidation products of phosphaalkenes, that is, λ^5^-phosphorane (*δ*_P_=10.0 ppm) and thiaphosphirane (*δ*_P_=−34.9 ppm) observed during oxidation of Mes*P=CPh_2_ with sulfur further supports our assignment.[20b] HRMS and X-ray crystallography (see below) further confirm that only the phosphole phosphorus is oxidized, thus encouraging our strategy to utilize the different reactivity of the two phosphorus centers in PPAs for the tuning of their electronic properties. Despite their differences in oxygen sensitivity, it is also evident that the ^31^P{^1^H} NMR spectroscopic chemical shifts of both P centers (see Table 1) of the PPAs are only marginally influenced by the electronic nature of the different aryl substituents in the 5-position, similar to previous observations of pyridyl-, thienyl-, or phenyl-substituted phospholes.[21] Furthermore, it can be noted that the coupling constants between the two phosphorus atoms decrease upon sulfurization of the phospholes.

**Table 1:** ^31^P{^1^H} NMR spectroscopic chemical shifts [ppm] of the PPAs (CD_2_Cl_2_) and ^3^*J*(P,P) coupling constants [Hz]

Entry	Substituent	^31^P{^1^H} phosphole	^31^P{^1^H} phosphaalkene	^3^*J*(P,P)
**5 a**	pyridyl	+10.4	+249.0	89.7
**5 b**	thienyl	+10.5	+249.4	92.8
**5 c**	phenyl	+12.2	+246.2	90.3
**6 a**	pyridyl	+52.3	+269.6	35.8
**6 b**	thienyl	+51.7	+268.6	36.7
**6 c**	phenyl	+53.4	+267.8	35.7
**7**	pyridyl^*^(AuCl)	+38.3	+279.4	75.6
**8**	pyridyl^*^(AuCl)_2_	+44.1	+199.0	45.0

**Figure 2:**
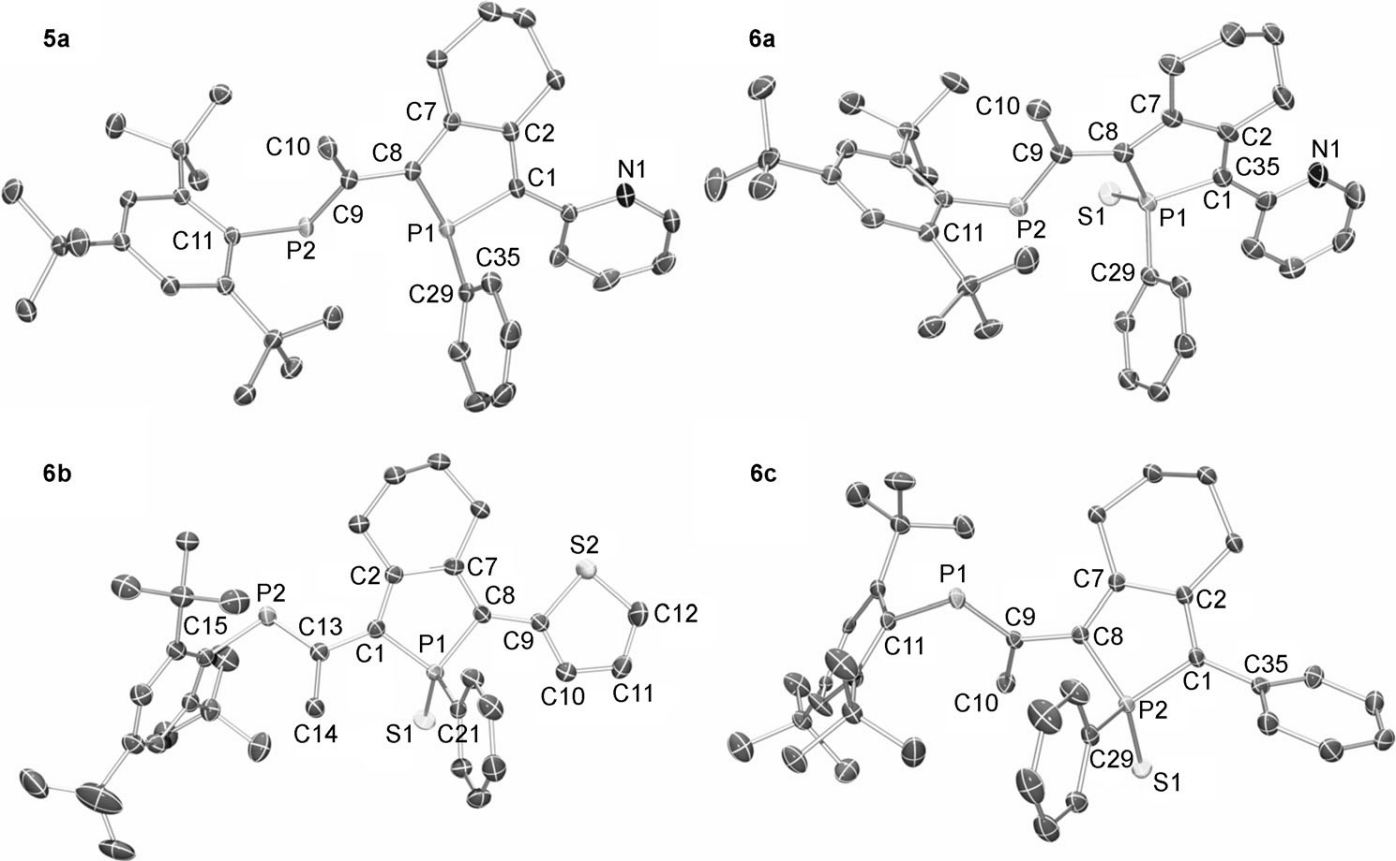
ORTEP plots of PPAs 5 a and 6 a–c (ellipsoids are drawn at 50 % probability level). Hydrogen atoms and disorder in the alkyl groups are omitted for clarity. Selected bond lengths [Å] and angles [°]: Compound 5 a: P1–C1 1.7984(19), P1–C8 1.811(2), P1–C29 1.828(2), P2=C9 1.705(2), P2–C11 1.8582(19), C1–C2 1.372(3), C2–C7 1.463(3), C7–C8 1.378(3), C8–C9 1.467(3); C1-P1-C8 91.28(9), C9-P2-C11 104.23(9), P2-C9-C8 115.61(15). Compound 6 a; P1–C1 1.802(4), P1–C8 1.823(4), P1–C29 1.821(3), P2=C9 1.706(4), P2–C11 1.839(4), C1–C2 1.367(5), C2–C7 1.482(5), C7–C8 1.377(5), C8–C9 1.455(5), P1–S1 1.9500(12); C1-P1-C8 94.17(17), C9-P2-C11 104.03(18), P2-C9-C8 114.67(3). Compound 6 b; P1–C1 1.813(2), P1–C8 1.814(2), P1–C21 1.822(2), P1–S1 1.9493(11), P2=C13 1.697(2), P2–C15 1.846(2), S2–C12 1.707(3), S2–C9 1.741(2), C1=C2 1.364(3), C1–C13 1.471(3), C2–C7 1.487(3), C7=C8 1.362(3), C9=C10 1.377(3), C10–C11 1.412(3), C11=C12 1.355(4), C13–C14 1.509(3); C13-P2-C15 101.93(10), C1-P1-C8 93.41(10), C1-C13-P2 115.99(16). Compound 6 c: P1=C9 1.6889(12), P1–C11 1.8580(12), P2–C1 1.7999(12), P2–C8 1.8214(12), P2–C29 1.8150(13), C1–C2 1.3487(17), C1–C35 1.4782(16), C2–C7 1.4955(16), C7–C8 1.3571(17), C8–C9 1.4762(16), P2–S1 1.9482(4); C1-P1-C8 94.17(17), C9-P2-C11 104.03(18), P2-C9-C8 114.67(3).

Figure 2 shows the crystal structures of **5 a** and **6 a**–**c**. Structures **6 a**–**c** confirm the selective oxidation of the σ^3^,λ^3^-phosphorus atom. The nitrogen heteroatom of the pyridyl substituent in both **5 a** and **6 a** has an s-*trans* relationship with respect to the phosphole phosphorus in the solid state, whereas the phosphaalkene resides in an s-*cis* arrangement to form a bidentate binding pocket in the solid state. In contrast, the solid-state structure of **6 b**,**c** shows an s-*trans* arrangement between the two phosphorus atoms. The sulfur heteroatom of the thienyl substituent in **6 b** has an s-*trans* relationship with respect to the phosphole phosphorus in the solid state. The bond lengths for the P=C double bonds in **5 a** and **6 a**–**c** are in the usual range as are the bond lengths within the phosphole core.[21] Furthermore the C–C bond bridging the phosphole and the phosphaalkene in **5 a** and **6 a**–**c** is typical for a Csp_2_–Csp_2_ single bond.

**Scheme 3:**
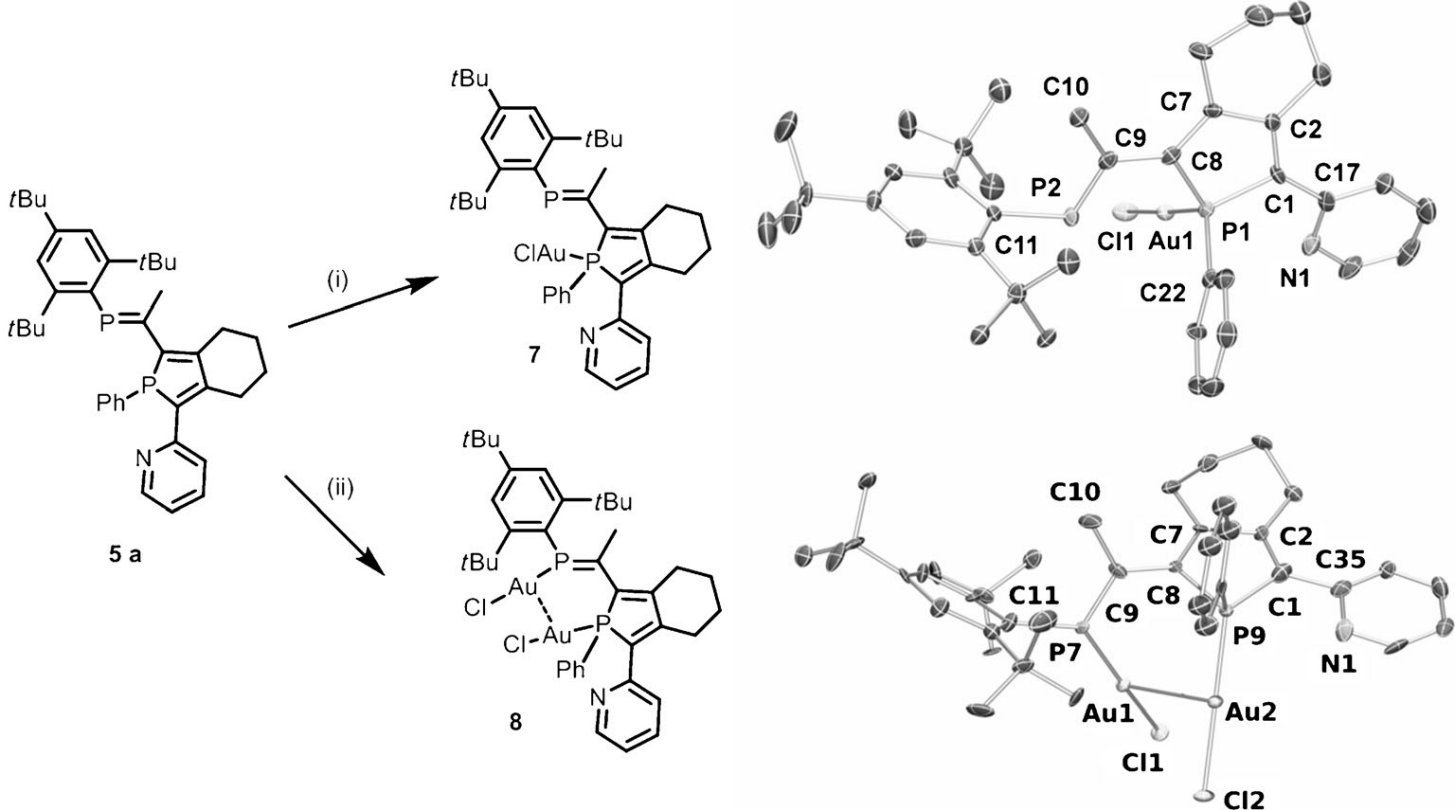
Coordination of AuCl to phosphole-phosphaalkene (PPA) 5 a. i) [AuCl(tht)] (1 equiv), CH_2_Cl_2_, RT, 1 h. 7 98 %. ii) [AuCl(tht)] (2 equiv), CH_2_Cl_2_, RT, 1 h. Compound 8 61 %. ORTEP plot of PPAs 7 and 8 (ellipsoids are drawn at 50 % probability level). Hydrogen atoms and disorder in the alkyl groups are omitted for clarity. Selected bond lengths [Å] and angles [°]: Compound 7: Au1–P1 2.234(2), Au1–Cl1 2.295(2), P1–C1 1.813(7), P1–C22 1.817(9), P1–C8 1.831(7), P2–C9 1.698(8); P1-Au1-Cl1 177.56(7). Compound 8: C1–P9 1.79(2), C9–P7 1.682(18), P7–Au1 2.226(4), P9–Au2 2.228(5), Cl1–Au1 2.280(4), Cl2–Au2 2.280(4), Au1–Au2 3.0692(11); P7-Au1-Cl1 170.85(19), P9-Au2-Cl2 175.6(2).

**Figure 3:**
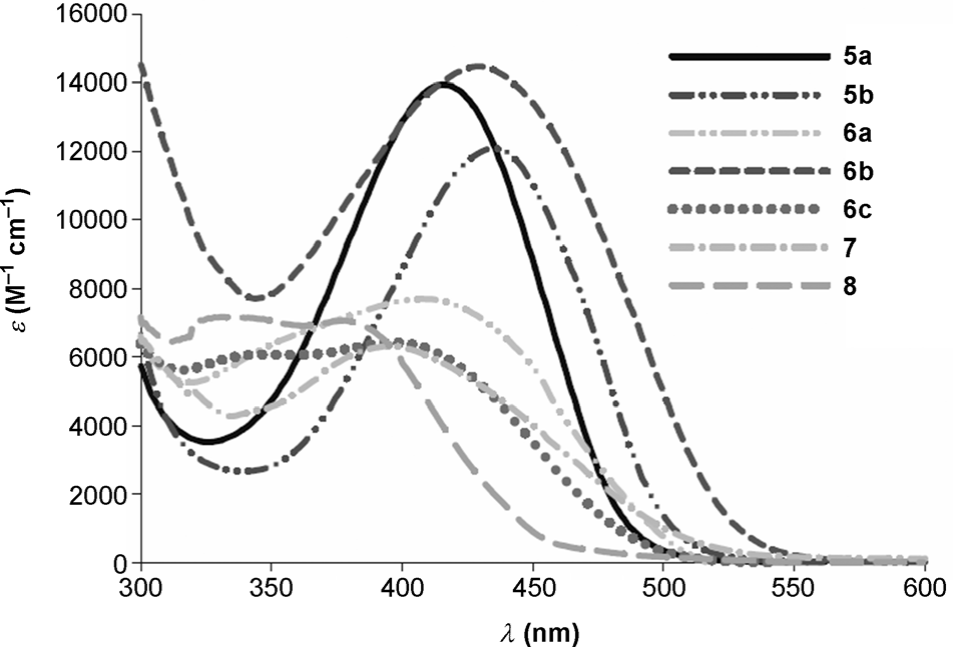
UV/Vis spectra of PPAs 5 a,b, 6 a–c, and gold complexes 7 and 8 in CH_2_Cl_2_ at room temperature. Synthesis of phosphole-phosphaalkenes (PPAs) 5,6 a–c.

To investigate the selectivity of metal coordination toward the two phosphorus heteroatoms, **5 a** was treated with [AuCl(tht)] (Scheme 3; tht=tetrahydrothiophene). With one equivalent of [AuCl(tht)], coordination occurs exclusively at the phosphole phosphorus to afford PPA **7**. The ^31^P{^1^H} NMR spectroscopic signal of the σ^4^,λ^4^-phosphorus center is shifted downfield by approximately 28 ppm to approximately 38 ppm. Analogous to the oxidation of the phosphorus atom of the phosphole ring by sulfur, the coordination by [AuCl(tht)] shifts the ^31^P{^1^H} NMR spectroscopic signal of the phosphaalkene phosphorus downfield (Table 1). The selective coordination of the σ^3^,λ^3^-phosphorus is verified by X-ray crystallography as shown in Figure 3. The solid-state structure of **7** shows similar structural parameters to **5 a** with a typical P=C bond length (1.698(8) Å) and expected Au^I^ coordination. The P-Au-Cl fragment shows a linear arrangement (177.56(7)°) with typical bond lengths (Au1–P1: 2.234(2) Å and Au1–Cl1: 2.295(2) Å). Interestingly, the phosphaalkene, the phosphole moiety, and the pending pyridine fragment are almost coplanar (P1-C8-C9-P2 −4.5(9)°) and calculated angles of the least-squares plane (l.s.pl.) between pyridine, phosphole, and phosphaalkene are 13.3(4) and 9.2(4)°, respectively. In contrast to the solid-state structures of **5 a** and **6 a**, the pyridine ring has an s-*cis* relationship with respect to the phosphole phosphorus in the solid state. Interestingly, treating **5 a** with two equivalents of [AuCl(tht)] results in an upfield shift of the ^31^P{^1^H} NMR spectroscopic signal of **8** to *δ*=199 ppm relative to *δ*=249 ppm for **5 a** of the phosphaalkene phosphorus concomitant with a similar chemical shift of the aurated phosphole. The solid-state structure of **8** confirms the formation of a dimetallic species and shows that one AuCl is coordinated to each phosphorus heteroatom; it also confirms the presence of a gold–gold interaction (3.069(1) Å) (Scheme 3). This metallophilic interaction twists the P=C out of the phosphole plane (P9-C8-C7-P7 −67(2)° and angle between l.s.pl. 64.9(10)°) and thereby breaks the effective conjugation between the phosphole moiety and the phosphaalkene in **8**. Again, the pyridine fragment is almost coplanar to the phosphole unit (9.7(10)°). (Scheme 3)

### Electronic absorption spectroscopy and cyclic voltammetry

The electronic absorption spectra of all PPAs are characterized by a strong absorption in the visible region, except for those of **6 c** and the Au_2_ complex **8**, which feature additional absorptions at higher energy (Figure 3 and Table 2). The observed absorption maxima are attributed to π→π* transitions of the conjugated system on the basis of quantum chemical calculations (see below). The nature of the 2,5-substituents and the phosphole-P oxidation state affect the optical properties of phospholes **5 a**,**b** and **6 a**–**c**. The presence of a thienyl group in **5 b** relative to a pyridyl group in **5 a** leads to a redshift of the lowest-energy absorption maximum by 20 nm. Sulfur oxidation of phospholes **5 a**,**b** to **6 a**,**b** induces a small blueshift of the absorption maximum and a redshift of the onset absorption (Δ*λ*_onset_ (**5 a**−**6 a**)=21 nm, Δ*λ*_onset_ (**5 b**−**6 b**)=27 nm). The longest wavelength absorption maximum of phenyl-substituted **6 c** is the highest in energy of all the PPAs investigated.

**Table 2:** UV/Vis spectroscopic data of PPAs 5 a,b and 6 a–c and gold complexes 7 and 8 in CH_2_Cl_2_ at room temperature

Entry	Substituent	*λ*_max_ [nm]	*ε* [m^−1^ cm^−1^]	*λ*_onset_ [nm]
**5 a**	pyridyl	415	14 000	485
**5 b**	thienyl	435	12 000v	502
**5 c**	phenyl	407	7700	506
**6 a**	pyridyl	429	14 500	529
**6 b**	thienyl	397	6400	491
**6 c**	phenyl	396	6300	514
**7**	pyridyl^*^(AuCl)	378	5900	455
**8**	pyridyl^*^(AuCl)_2_	415	14 000	485

Coordination of one AuCl to **5 a** results in a blueshift of *λ*_max_ by 19 nm, and the *λ*_onset_ shifts by 29 nm to lower energies. Interestingly, coordination of a second AuCl fragment as in **8** results in a further hypsochromic shift, both relative to **5 a** and **7** for *λ*_max_. In contrast to the monoaurated complex **7**, the *λ*_onset_ is significantly shifted to higher energies upon complexation of a second AuCl fragment. As described above, the Au–Au interaction in **8** twists the P=C unit out of the phosphole plane and thereby breaks effective conjugation between the two chromophores, which explains the hypsochromic shift that is observed in the UV/Vis spectrum of **8**. When comparing the UV/Vis data of **5 a** with a related phosphole, 1-phenyl-2,5-bis(2-pyridyl)phosphole (*λ*_max_=374 nm), it is noticeable that the incorporation of a phosphaalkene motif induces a bathochromic shift of Δ*λ*_max_=41 nm.[21] The same trend is observed for **6 c** when comparing with 1,2,5-triphenylthiooxophosphole (Δ*λ*_max_=17 nm), whereas **6 b** shows almost the same end absorption as 1-phenyl-2,5-bis(2-thienyl)thiooxophosphole (Δ*λ*_max_=3 nm),[22] thereby suggesting an electronically similar effect of the phosphaalkene and the thiophene moiety.

PPAs **5 a**,**b** and **6 a**–**c** exhibit a rich electrochemistry and their cyclic voltammograms feature multiple reduction and oxidation processes (Table 3). The first reduction of **5 a** and **5 b** occurs at very similar potential of −2.16 and −2.18 V, respectively, and is thus not influenced by the pyridyl or thienyl auxiliary substituent. The situation is somewhat different in the reduction potentials observed for oxidized **6 a**–**c**, which can be detected over a broader potential range of −1.90 to −2.05 V depending on the substituent (all potentials are measured in CH_2_Cl_2_ and given versus the ferrocene/ferrocenium (Fc^+/0^) couple). In general, electron uptake by oxidized **6 a**–**c** occurs at milder potentials than those of **5 a**,**b**.

**Table 3:** Electrochemical data for 1 mm solutions of the PPAs in CH_2_Cl_2_ (0.1 m NBu_4_PF_6_); *ν*=100 mV s^−1^ (all potentials are given versus Fc^+/0^)

Entry	*E*_p,c_ [V]				*E*_p,a_ [V]		
**5 a**	–	−2.16	−2.27	+0.48	+0.81	+0.99	+1.26
**5 b**	–	−2.18	−2.29	+0.37	+0.62	+0.99	+1.08
**6 a**	–	−1.90	−2.20	+0.50	+0.90	+1.08	–
**6 b**	–	−1.99	−2.28	+0.39	+0.59	+1.02	+1.21
**6 c**	–	−2.05	−2.27	+0.51	+0.70	+1.07	–
**7**	−1.53	−1.86	−2.24	+0.66	+1.09	–	–
**8**	−0.97	−1.57	−1.69	+1.04	–	–	–

When comparing PPAs **5 a**,**b** to literature-known phospholes that contain an additional pyridyl or thienyl group instead of the phosphaalkene,[21] a large difference in reduction potential can be observed. Whereas **5 a** displays a first reduction at −2.16 V, the equivalent process occurs at significantly more negative potential in 1-phenyl-2,5-bis(2-pyridyl)phosphole at −2.45 V.[21] The electronic effect of the phosphaalkene on the reduction potential of **6 a**,**b** is, however, negligible as they are almost identical (the difference is 20–40 mV) to those of 1-phenyl-2,5-bis(2-pyridyl)thiooxophosphole and 1-phenyl-2,5-bis(2-thienyl)thiooxophosphole, respectively. The first reduction potentials of gold complexes **7** and **8** are observed at significantly milder potentials than both **5 a** and **6 a**. It should be noted, however, that **8** was not stable during the voltammetry experiments. The reported values in Table 3 are from the first scan, whereas new reduction and/or oxidation waves are visible in the second scan, possibly due to cleavage of the highly twisted structure or demetalation.

The first oxidation potentials of **5 a** and **5 b** are almost identical to those of **6 a** and **6 b**, respectively, which points towards the absence of any contribution of the phosphole phosphorus in the HOMO and large contributions of the auxiliary substituents, interconnected by the butadiene unit within the phosphole. The similarities of the first oxidation potentials between **5 a**,**b** and their corresponding thiooxophospholes **6 a**,**b** stand in contrast to the observation of, for example, 1-phenyl-2,5-bis(2-thienyl)phosphole, in which oxidation by sulfur was found to lead to a shift of the mildest oxidation potential by 280 mV.[21] The above comparisons imply that the HOMOs of **5** and **6** reside mainly on the extended π system that includes the phosphaalkenes and possibly the butadiene of the phosphole, but with only marginal contributions of the auxiliary pyridyl or thienyl substituent. The rich oxidative chemistry of **5** and **6** at relatively mild potentials indicates closely spaced occupied frontier orbitals, which was also found in DFT calculations. The first oxidation potentials of gold complexes **7** and **8** are observed at more positive potentials than both **5 a** and **6 a**.

### Density functional theory (DFT) calculations

**Figure 4:**
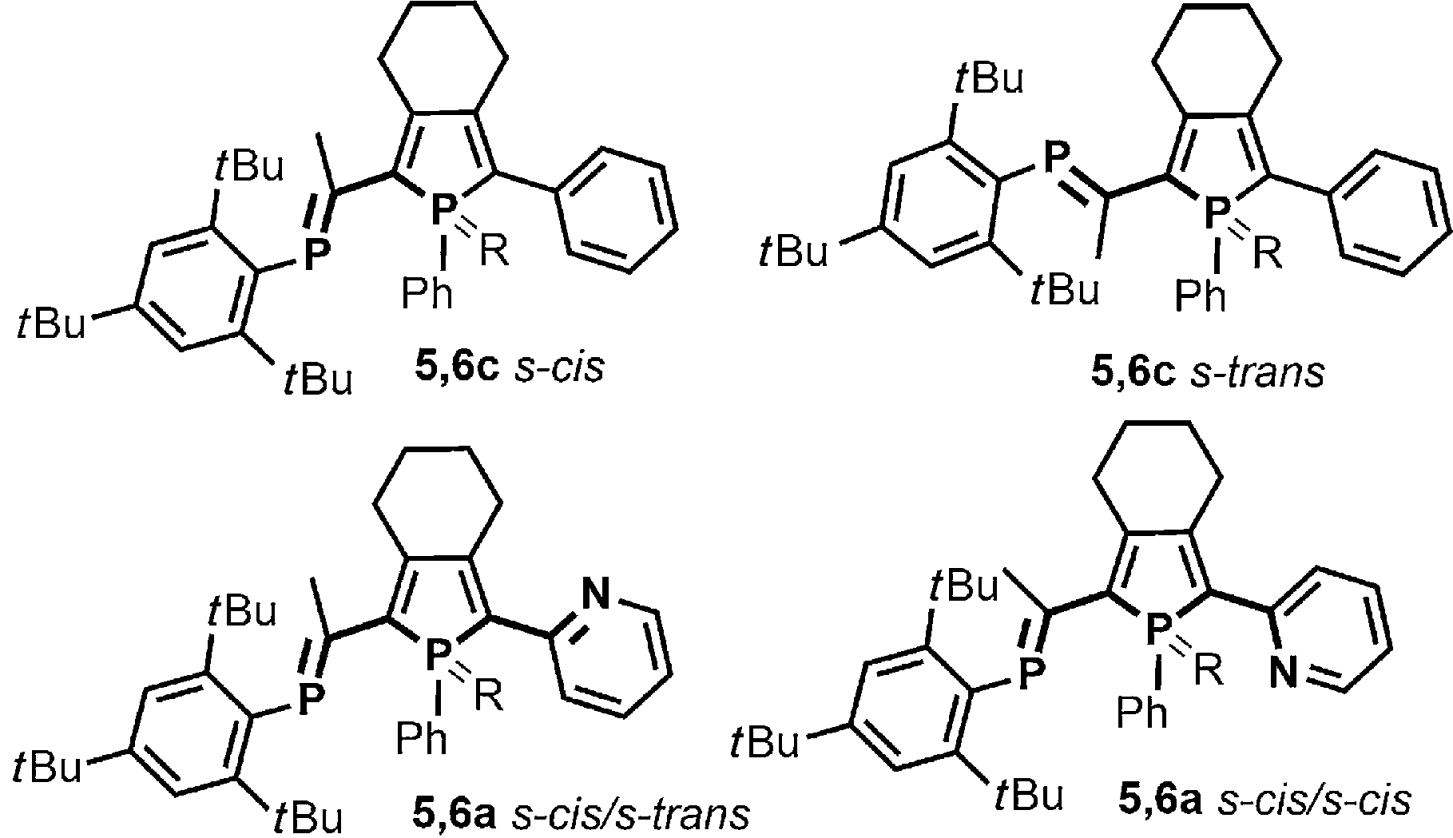
The relative conformations between the P=C double bond and the orientation of the phosphole. The designation s-*cis* is used when the torsion angle between the two phosphorus heteroatoms is close to 0° (top left) and *anti* when the torsion angle between the two phosphorus heteroatoms is close to 180° (top right). The designation s-*cis*/s-*trans* is used when the torsion angle between the two phosphorus heteroatoms is close to 0° and the torsion angle between the phosphole phosphorus and the nitrogen (or sulfur for 5,6 b) is close to 180° (bottom left); s-*cis*/s-*cis* is used when the torsion angle between the two phosphorus heteroatoms is close to 0° and the torsion angle between the phosphole phosphorus and the nitrogen (or sulfur for 5,6 b) is close to 0° (bottom right).

DFT calculations of the different PPAs were performed to gain further insight into the conformations of the PPA, delocalization through the π systems, and the influence of the phosphaalkene moiety. Computations were performed at the DFT B3LYP/6-311G** level of theory, which is known to perform well for main group systems.[7k, 23] As a first step, we decided to investigate the energy difference between the s-*cis* and s-*trans* conformations of PPAs **5 c** and **6 c** with respect to the torsion angle between the phosphole and the phosphaalkene moiety (Figure 4). Since both conformations were observed in solid-state structures of **5** and **6**, the expectation was that the energy difference between the two arrangements of the phosphorus heteroatoms would be very small. For both **5 c** and **6 c**, the s-*trans* arrangement is favored by 0.53 and 1.60 kJ mol^−1^, respectively, which is in good agreement with the observations from X-ray crystallography and also indicates that both conformers can be expected to be present in solution.

As the pyridyl-phospholes in **5 a** and **6 a** are potential bidentate metal-binding sites, the conformational preference of these two subunits relative to each other was investigated. According to the X-ray crystallographic structures of the compounds, the s-*cis* conformations of the two phosphorus heteroatoms were kept, and the torsion angles between the phosphole phosphorus and the nitrogen were set either close to 0° in s-*cis*/s-*trans* and close to 180° in the s-*cis*/s-*cis*. The structures were then allowed to fully relax during geometry optimization (Figure 4). In both **5 a** and **6 a**, the s-*cis*/s-*trans* conformations were favored by 1.35 and 2.08 kJ mol^−1^, respectively, values that correlate well with the X-ray crystallographic structures. However, we assume that the rotational barrier is very low, which might also explain the pyridine conformation found in gold complexes **7** and **8**, both of which show the s-*cis*/s-*cis* conformation. Owing to the small energy difference found between the different conformers for **5 a**,**c** and **6 a**,**c**, it was decided that the geometries for geometry optimization **5 b** and **6 b** were based on the structures obtained from the X-ray crystallographic analysis.

**Figure 5:**
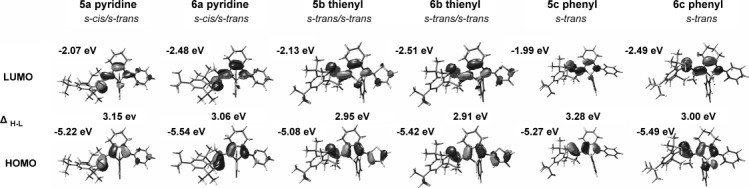
Calculated HOMO and LUMO with orbital energies [eV] for PPAs 5 and 6 at the DFT B3LYP/6-311G** level of theory; Δ_H–L_ HOMO–LUMO splitting in eV.

Graphical representation of the calculated FMOs of compounds **5 a**–**c** and **6 a**–**c** are shown in Figure 5. The FMOs have almost exclusively a p_π_ character. In agreement with the electronic absorption spectra, both the phosphaalkene moiety and the π-conjugated backbone of the phosphole are participating in both the HOMO and the LUMO. The lowest energy absorptions in the UV/Vis spectra can therefore be expected to result from π→π* transitions. The trends observed for the PPAs from the electronic absorption spectroscopy are also observed from the LUMO–HOMO energies of the DFT calculations. The longest-wavelength absorption maximum of phenyl-substituted **5 c** and **6 c** are highest, and thienyl-substituted **5 b** and **6 b** are the lowest in energy of all PPAs. The bathochromic shifts of the *λ*_onset_ upon oxidation by sulfur is also reflected in the values of the calculated LUMO–HOMO gaps. The calculated LUMO–HOMO gaps for **6 a**,**b** of 3.06 and 2.91 eV are in excellent agreement with the values obtained for the lowest energy absorption maxima of 3.05 (407) and 2.89 eV (429 nm), respectively, obtained from the UV/Vis absorption experiments.

The calculations also show that the phosphole-phosphorus does not participate in the HOMOs of **5** and **6**, which are localized on the phosphaalkene and the butadiene with contributions from the auxiliary substituents. These results are thus in excellent agreement with the conclusions from the electrochemical studies. In general, PPAs **6 a**,**b** are more difficult to oxidize than **5 a**,**b**; this is reflected well in the energies of the calculated HOMOs, which are higher in energy for **5 a**,**b** than for **6 a**,**b**, even though the quantitative agreement is not excellent.

As for the HOMOs, the LUMOs are located on the phosphaalkene and the butadiene unit with contributions from the substituents. However, for PPAs **5**, there is a contribution from the phosphorus lone pair of the phosphole, whereas the contribution of the P=S is smaller for the oxidized PPAs **6**. The LUMO lies higher in energy for **5 a**,**b** relative to **6 a**,**b**, which reflects the experimentally obtained reduction potentials in which **6 a**,**b** are reduced at milder potentials than **5 a**,**b**.

**Figure 6:**
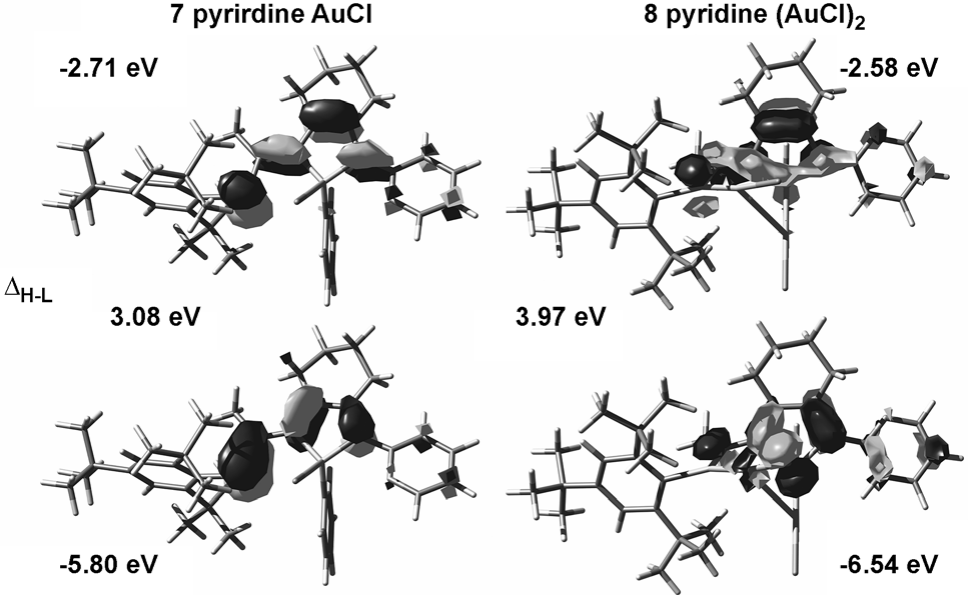
Calculated HOMO and LUMO with orbital energies [eV] for PPAs 7 and 8 at the DFT PBE1PBE/6-311G**/LANL2DZ level of theory; Δ_LUMO–HOMO_ HOMO–LUMO splitting in eV.

Gold complexes **7** and **8** were calculated at the PBE1PBE/6-311G**/LANL2DZ level of theory. Similar to their precursor **5 a**, both the HOMOs and the LUMOs of **7** and **8** are localized on the phosphole and phosphaalkene motifs with small contributions from the pyridyl substituent. The AuCl moiety connected to the phosphaalkene of **8** contributes to the HOMO with a d_*xy*_ orbital localized on the Au and a p orbital localized on the chlorine atom. The contribution of the phosphaalkene moiety in **8** is decreased owing to the twist between the phosphaalkene and the phosphole ring (dihedral angle of 78.3°) relative to the situation in **5** and **6** in which the two moieties are generally are more coplanar (dihedral angles of (150±15)° both in the experimental and the theoretical data). This twist is also responsible for the relatively high-energy transition in the UV/Vis spectrum of **8**. The HOMO and the LUMO are lower in energy for **7** and **8** than those of **5 a** and **6 a**, which is in good agreement with the electrochemical data in which both **7** and **8** are more difficult to oxidize than **5** and **6**. Diaurate complex **8** is the most difficult compound to be oxidized of the series (Figure 6).

## Conclusion

In summary, we have established a synthetic versatile route to the first examples of phosphole-phosphaalkenes that unite two differently hybridized (σ^3^,λ^3^, and σ^2^,λ^3^) phosphorus heteroatoms as integral parts of one fully π-conjugated framework. We have also demonstrated that selective modifications of these compounds at the phosphole unit can be achieved by using oxidation (S_8_) and metal coordination (AuCl). Ultimately, a diaurated species was obtained with intermetallic distances of 3.069(1) Å. The Au–Au interaction induces a twist in the molecular structure that is responsible for a significant decrease in the conjugation path. UV/Vis spectroscopic studies show that the phosphaalkene is part of the extended π system and thereby shifts the lowest-energy absorption maxima towards lower energies than similar phosphole reference compounds that lack the phosphaalkene unit. Cyclic voltammetry and ab initio calculations both indicate that the phosphaalkene moiety is mainly involved in the HOMO, whereas the phosphole phosphorus participates in the LUMO of PPAs **5**. This result encourages further manipulations of the PPAs to tune the optical properties of this new class of π-conjugated materials and the use of such materials for organic electronics applications.

## Experimental Section

### Materials and general methods

Chemicals were purchased from Sigma–Aldrich, VWR, ABCR, and Fisher Scientific and were used as received. THF and Et_2_O were distilled from sodium/benzophenone. CH_2_Cl_2_ was distilled from calcium hydride or freshly purified using MBraun SPS-800 drying columns. All reactions were performed under an inert atmosphere of N_2_ or Ar using standard Schlenk techniques. Column chromatography was performed using Merck silica gel Si-60 Å (35–70 or 0.063–0.200 mm) or on basic alumina (Aldrich type 5016A, 150 mesh, 58 Å).

### NMR spectroscopy

^1^H NMR spectra were recorded at ambient temperature using a JEOL Eclipse 400 MHz spectrometer (operating at 399.8 MHz), a Varian Mercury Plus 300 MHz spectrometer (operating at 300.03 MHz) or Bruker AM300, AM400, or AM500 instruments. Chemical shifts are given in ppm and referenced internally to the residual solvent signal (CHCl_3_, *δ*_H_=7.26 ppm or CH_2_Cl_2_, *δ*_H_=5.32 ppm). ^13^C NMR spectra were recorded on the same instrument (100.5 or 75.4 MHz) and were also referenced internally to the residual solvent signal (CHCl_3_, *δ*_c_=77.1 ppm, central signal, CH_2_Cl_2_, *δ*_c_=54.00 ppm). ^31^P{^1^H} NMR spectra were recorded using a JEOL Eclipse 400 MHz spectrometer (operating at 161.8 MHz), referenced externally to 85 % H_3_PO_4_ (aqueous).

### UV/Vis spectroscopy

UV/Vis measurements were performed using a Varian Cary 50 instrument or a Varian Cary 5000 instrument at room temperature. The measurements were performed in 1×1 cm^2^ optical quartz cells as solutions in CH_2_Cl_2_.

### Electrochemistry

The electrochemical measurements were conducted in dry CH_2_Cl_2_ (distilled from calcium hydride). An Autolab potentiostat with a GPES electrochemical interface (Eco Chemie) was used for cyclic voltammetry measurements with a glassy carbon disc (diameter 3 mm, freshly polished) as the working electrode. The counter electrode was a glassy carbon rod and the reference electrode a nonaqueous Ag^+^/Ag electrode (a silver wire immersed into 10 mm AgNO_3_ in acetonitrile) with a potential of −0.08 V versus the ferrocenium/ferrocene (Fc^+^/Fc) couple in CH_2_Cl_2_ as an external standard. The cyclic voltammograms were conducted at a scan rate (*ν*) 100 mV s^−1^. All solutions were conducted on 1 mm solutions of the phospholes in dry CH_2_Cl_2_ with 0.1 m tetrabutylammonium hexafluorophosphate (NBu_4_PF_6_) as supporting electrolyte. Before all measurements, oxygen was removed by bubbling with argon and during the measurement all samples were kept under argon.

### Mass spectrometry

Low-resolution mass spectrometry was performed using a Thermo LCQ Deca XP Max with direct injection electrospray ionization (ESI). A small amount of the compounds were dissolved in a solution of approximately 2.5 mm AgBF_4_ in MeOH. High-resolution mass spectrometry (HRMS) were performed using a high-resolution and FTMS+pNSI mass spectrometer (orbitrapXL), using a MicroTOF spectrometer with ESI core at University of Muenster, or using a Varian MAT 311, Waters Q-TOF 2, or ZabSpec TOF Micromass instrument at CRMPO of Rennes 1. MALDI-MS spectra were obtained in a Ditranol matrix using a nitrogen laser accumulating 50 laser shots and a Bruker Microflex LT MALDI-TOF mass spectrometer.

### Computational details

Calculations were carried out with the Gaussian 09 program package (G09 rev. A.02). The PPAs were fully optimized at the B3LYP/6-311G** level of theory and were determined as true minima by inspection of their harmonic frequencies with no imaginary frequencies. Molecular orbitals were calculated at the same level of theory. Gold complexes were calculated at the PBE1PBE level of theory with a 6-311G** basis set for CHNPCl and a LANL2DZ with ECP for gold.[24]

### Synthesis

**Phosphaalkene 3**: Mg turnings (350 mg, 14.4 mmol) were added to 25 mL three-necked round-bottomed flask equipped with a condenser, a stirring bar, and a dropping funnel. THF (5 mL) was added and the suspension was heated to reflux. EtBr (0.25 mL, 3.4 mmol) was added in one portion, and EtBr (0.75 mL, 10.2 mmol) dissolved in THF (20 mL) was added to the dropping funnel. The solution was added dropwise to the Mg suspension that was heated to reflux, and the Mg turnings started to disappear. After stirring for 30 min, the solution was transferred to a 250 mL three-necked round-bottomed flask that contained THF (100 mL). The pale gray solution was brought to reflux, and 1,7-octadiyne (1.5 mL, 11.3 mmol) in THF (40 mL) was added dropwise to the solution that was heated to reflux. The pale gray solution became clear and a white precipitate was formed. Mes*P=CCH_3_Br **2** (1.80 g, 4.70 mmol), [Pd(dba)_2_] (269 mg, 0.47 mmol), and PPh_3_ (123 mg, 0.47 mmol) were added to the white suspension. The reaction mixture turned dark green and was stirred at reflux for 4–5 h until all the starting material had disappeared by TLC (silica, pentane). If no change could be detected by TLC and there was still starting material left, additional [Pd(dba)_2_] was added to complete the reaction. The reaction mixture was cooled to room temperature and filtered through a plug of silica, which was rinsed with CH_2_Cl_2_. The solvent was removed under vacuum and subsequently subjected to column chromatography on silica with pentane as eluent to yield an off-white solid. Crystallization from CH_2_Cl_2_/CH_3_CN gave colorless crystals. Yield: 1.88 g, 4.60 mmol (98 %).

*Alternative procedure*: Mes*P=CCH_3_Br **2** (1.50 g, 3.90 mmol) was dissolved in Et_2_NH (30 mL), and the solution was deaerated by bubbling with argon. CuI (70 mg, 0.37 mmol), [Pd(PPh_3_)_2_Cl_2_] (223 mg, 0.32 mmol), and finally 1,7-octadiyne (1.0 mL, 8.0 mmol) were added. The reaction mixture was stirred for 3 h at room temperature until no Mes*P=CCH_3_Br could be detected by TLC. The reaction mixture was concentrated and the solid extracted with pentane. Column chromatography as above. Yield: 1.04 g, 2.54 mmol (65 %). ^1^H NMR (400 MHz, CDCl_3_): *δ*=1.28 (d, ^3^*J*(P,H)=13.6 Hz, 3 H; C*H*_3_), 1.33 (s, 9 H; *p*-*tert*-Bu), 1.48 (s, 18 H; *o*-*tert*-Bu), 1.69 (m, 4 H; C≡CCH_2_C*H_2_*), 1.93 (t, ^4^*J*(P,H)*=*2.6 Hz, 1 H; C≡CC*H*), 2.25 (m, 2 H; C≡CCH_2_), 2.47 (m; C≡CCH_2_), 7.40 ppm (s, 2 H; Ar); ^31^P{^1^H} NMR (161.8 MHz, CDCl_3_): *δ*=275.3 ppm; ^13^C NMR (100 MHz, CDCl_3_): *δ*=18.0 (s; C≡CCH_2_*C*H_2_), 19.9 (d, ^4^*J*(P,C)=3.8 Hz; C≡CCH_2_*C*H_2_), 25.4 (d, ^2^*J*(P,C)=14.4 Hz; CH_3_), 27.7 (s; C≡C*C*H_2_CH_2_), 27.8 (s; C≡C*C*H_2_CH_2_), 31.3 (s; *p*-C(*C*H_3_)_3_), 32.5 (d, ^4^*J*(P,C)=6.2 Hz; *o*-C(*C*H_3_)_3_), 35.0 (s; *o*-*C*(CH_3_)_3_), 37.9 (s; *p*-*C*(CH_3_)_3_), 68.4 (s; C≡*C*H), 84.3 (s; *C*≡CH), 85.8 (d, ^3^*J*(P,C)=29.1 Hz; P=C–C≡*C*), 97.5 (d, ^2^*J*(P,C)=18.6 Hz; P=C–*C*≡C), 121.6 (s; *meta*-Ph), 136.1 (d, ^1^*J*(P,C)=60.3 Hz; P–C), 150.3 (s; *para*-Ar), 153.7 (s; *ortho*-Ar), 162.0 ppm (d, ^1^*J*(P,C)=32.4 Hz; P=C); MS (ESI): *m*/*z*: 515.4 (100) [*M*+Ag]^+^; HRMS (ESI): *m*/*z* calcd for C_28_H_41_NaP: 431.28436; found: 431.2843 [*M*+Na]^+^; elemental analysis calcd (%) for [C_28_H_41_P+1/3 CH_3_CN]: C 81.53, H 10.02; found: C 81.73, H 10.02.

**Phosphaalkene 4 a**: 1-(2-Pyridyl)octa-1,7-diyne (367 mg, 2.0 mmol) and Mes*P=CCH_3_Br **2** (500 mg, 1.30 mmol) were added to a deaerated solution of [Pd(PPh_3_)_2_Cl_2_] (71 mg, 0.10 mmol) and CuI (10 mg, 0.05 mmol) in Et_2_NH (10 mL). Upon addition of the reagents, the yellow solution turned to a suspension. The mixture was stirred overnight, and following morning it was filtered and concentrated. Column chromatography (40 % pentane in Et_2_O (*R*_f_=0.40)) gave the product as a pale yellow solid. Yield: 466 mg, 0.96 mmol (74 %). ^1^H NMR (400 MHz, CDCl_3_): *δ*=1.28 (d, ^3^*J*(P,H)=13.6 Hz, 3 H; C*H*_3_), 1.32 (s, 9 H; *p*-*tert*-Bu), 1.48 (s, 18 H; *o*-*tert*-Bu), 1.78 (m, 4 H; C*H_2_*), 2.50 (m, 4 H; C*H_2_*), 7.18 (m, 1 H; pyridine H5), 7.38 (m, 1 H; pyridine H4), 7.40 (s, 2 H; Mes*), 7.60 (m, 1 H; pyridine H4), 8.54 ppm (d, 1 H; pyridine H6); ^31^P{^1^H} NMR (161.8 MHz, CDCl_3_): *δ*=274.7 ppm; ^13^C NMR (100 MHz, CDCl_3_): *δ*=19.0 (s; Py-C≡CCH_2_*C*H_2_), 19.9 (d, ^4^*J*(P,C)=4.8 Hz; C≡CCH_2_*C*H_2_), 25.4 (d, ^2^*J*(P,C)=14.5 Hz; CH_3_), 27.6 (s; Py-C≡C*C*H_2_CH_2_), 28.0 (d, ^5^*J*(P,C)=4.6 Hz; C≡C*C*H_2_CH_2_), 31.4 (s; *p*-C(*C*H_3_)_3_), 32.6 (d, ^4^*J*(P,C)=6.8 Hz; *o*-C(*C*H_3_)_3_), 35.0 (s; *o*-*C*(CH_3_)_3_), 38.0 (s; *p*-*C*(CH_3_)_3_), 80.6 (s; C≡*C*Py), 85.9 (d, ^3^*J*(P,C)=18.6 Hz; P=C–C≡*C*), 90.6 (s; *C*≡CPy), 97.5 (d, ^2^*J*(P,C)=29.3 Hz; P=C–*C*≡C), 121.6 (s; *meta*-Ph), 122.3 (s; C5, Py), 126.8 (s; C3, Py), 136.8 (s; C4, Py), 136.2 (d, ^1^*J*(P,C)=60.3 Hz; P–C), 143.9 (s; C2, Py), 149.8 (s; C6, Py), 150.3 (s; *para*-Ar), 153.7 (s; *ortho*-Ar), 162.1 ppm (d, ^1^*J*(P,C)=32.3 Hz; P=C); MS (ESI): *m*/*z*: 592.5 (100) [*M*+Ag]^+^; HRMS (ESI): *m*/*z* calcd for C_33_H_44_NNaP: 508.3109; found: 508.3106 [*M*+Na]^+^; *m*/*z* calcd for C_33_H_45_NNaP: 486.3290; found: 486.3303 [*M*+H]^+^; elemental analysis calcd (%) for C_33_H_44_NP: C 81.61, H 9.13; found: C 81.59, H 8.92. The assignment was based on the assignment of 1-(2-pyridyl)octa-1,7-diyne.[25]

Phosphaalkene **4 b** and **4 c** were prepared in a procedure analogous to that used for the preparation of **4 a** in 96 and 54 % yield, respectively (see the Supporting Information).

**Phosphaalkene 5 a**: Phosphaalkene **4 a** (250 mg, 0.51 mmol) and [Cp_2_ZrCl_2_] (149 mg, 0.51 mmol) were added to a 150 mL Schlenk flask, and the two compounds were dried for 2 h. THF (10 mL) was added, and the yellow solution was cooled to −78 °C. *n*BuLi (0.41 mL, 1.02 mmol, 2.5 m in hexanes) was added, and the resulting green solution was stirred at this temperature for 1 h and then warmed to room temperature upon which it turned red and left overnight. The following morning the solution was cooled to −78 °C and PhPBr_2_ (0.11 mL, 0.56 mmol) was added. The red solution was warmed to room temperature and stirred for 4 h followed by a filtration through basic alumina. The alumina was rinsed with THF (3×15 mL) until the color of the eluent disappeared. The yellow-orange solution was concentrated to yield **5 a** as a yellow solid. In cases in which purification was needed, the phosphole was purified by column chromatography on silica with 10 % Et_2_O in pentane or by recrystallization from either pentane or CH_2_Cl_2_/CH_3_CN. Yield: 127 mg, 0.21 mmol (42 %). ^1^H NMR (400 MHz, CDCl_2_): *δ*=1.23 (s, 9 H; *tert*-Bu) 1.30 (s, 9 H; *tert*-Bu), 1.37 (s, 9 H; *tert*-Bu), 1.52 (d, ^3^*J*(P,H)=13.5 Hz, 3 H; CH_3_), 1.70 (m, 2 H; CCH_2_C*H_2_*), 1.82 (m, 2 H; CCH_2_C*H_2_*), 2.81 (m, 2 H; C*CH_2_*CH_2_), 3.11 (m, 1 H; C*CH_2_*CH_2_), 3.33 (m, 1 H; C*CH_2_*CH_2_), 6.95 (ddd, ^3^*J*(H,H)=7.4 Hz, 4.8 Hz, ^5^*J*(H,H)=1.1 Hz, 1 H; pyridyl, H5) 7.15–7.34 (m, 3 H; Ph), 7.34–7.41 (m, 4 H; Mes*, Ph), 7.41–7.43 (m, 1 H; pyridyl, H3), 7.52 (ddd, ^3^*J*(H,H)=7.9, 7.4 Hz, *J*(H,H)=1.9 Hz, 1 H; pyridyl H4), 8.47 ppm (ddd, ^3^*J*(H,H)=4.8 Hz, ^4^*J*(H,H)=1.9 Hz, ^5^*J*(H,H)=1.0 Hz, 1 H; pyridyl H6); ^31^P NMR (161.8 MHz, CD_2_Cl_2_): *δ*=10.4 (d, ^3^*J*(P,P)=89.7 Hz), 249.0 ppm (d, ^3^*J*(P,P)=89.7 Hz); ^13^C NMR (100 MHz, CDCl_2_): *δ*=23.7 (s; CCH_2_*C*H_2_), 24.0 (s; CCH_2_*C*H_2_), 26.6 (dd, *J*(P,C)=14.4, 14.6 Hz; P=C*C*H_3_), 29.7 (s; C*C*H_2_CH_2_, C9), 30.6 (d, *J*(P,C)=4.9 Hz; C*C*H_2_CH_2_, C12), 31.6 (s, CH_3_, *para*-*t*Bu), 32.4 (d, *J*(P,C)=6.4 Hz; CH_3_, *ortho*-*t*Bu), 32.9 (d, *J*(P,C)=7.4 Hz; CH_3_, *ortho*-*t*Bu), 35.4 (s; *para*-*C*(CH_3_)_3_), 38.2 (s; *ortho*-*C*(CH_3_)_3_), 38.4 (s; *ortho*-*C*(CH_3_)_3_), 120.7 (s; C5, pyridyl), 122.2 (d, *J*(P,C)=13.7 Hz; CH, phenyl), 123.8 (d, *J*(P,C)=9.5 Hz; C3, pyridyl), 128.9 (d, *J*(P,C)=8.5 Hz; CH, phenyl), 130.0 (s; CH, phenyl), 132.4 (d, *J*(P,C)=11.0 Hz; *ipso*-C, phenyl), 135.4 (s; CH, Mes*), 135.2 (s; CH, Mes*), 136.3 (s; C4, pyridyl), 138.8 (dd, *J*(P,C)=6.5 Hz, 61.9 Hz; *ipso*-C, Mes*), 143.5 (dd, *J*(P,C)=11.0, 3.0 Hz; C14),144.5 (dd, *J*(P,C)=18.4, 11.4 Hz; C7), 149.6 (dd, *J*(P,C)=10.0, 4.5 Hz; C13), 149.7 (s; C6, pyridyl), 150.5 (s; *para*-C, Mes*), 152.7 (d, *J*(P,C)=24.2 Hz; C8), 154.2 (s; *ortho*-C, Mes*), 154.7 (s; *ortho*-C, Mes*), 156.5 (d, *J*(P,C)=18.6 Hz; C2, pyridyl), 179.7 ppm (dd, *J*(P,C)=16.2 Hz, 43.3 Hz; P=C); MS (ESI): *m*/*z*: 700.4 [*M*+Ag]^+^; elemental analysis calcd (%) for [C_39_H_49_NP_2_+CH_2_Cl_2_]: C 70.79, H 7.57, N 2.06; found: C 70.80, H 7.50, N 1.88.

Phosphaalkene **5 b** was prepared analogously to **5 a**.

**Phosphaalkene 6 a**: An excess amount of sulfur was added to PPA **5 a** (100 mg, 0.17 mmol) in CH_2_Cl_2_ (20 mL), and the red reaction mixture was stirred overnight. The solvent was removed under vacuum. The mixture was subjected to column chromatography in pentane to elute the excess amount of sulfur. The polarity was then increased to 40:60 pentane/Et_2_O to elute the product. After removal of the solvent, **6 a** was obtained as a yellow solid. Yield: 42 mg, 0.067 mmol (40 %). ^1^H NMR (400 MHz, CD_2_Cl_2_): *δ*=1.31 (s, 9 H; *tert*-Bu) 1.37 (s, 9 H; *tert*-Bu), 1.39 (d, ^3^*J*(P,H)=10.1 Hz, 3 H; CH_3_), 1.54 (s, 9 H; *tert*-Bu), 1.88 (m, 4 H; CCH_2_C*H_2_*), 3.10–3.40 (m, 4 H; CH_2_), 6.95 (m, 1 H; pyridyl, H5), 7.43 (s, 1 H, Mes*), 7.47 (s, 1 H, Mes*), 7.48–7.55 (m, 3 H, Ph), 7.58–7.60 (m, 2 H, Ph), 7.58–7.60 (m, 2 H; Ph), 7.61 (m, 1 H; pyridyl, H3), 7.94–7.99 (m, 1 H; pyridyl, H4), 8.59–8.61 ppm (m, 1 H; pyridyl H6); ^31^P NMR (161.8 MHz, CD_2_Cl_2_): *δ*=52.3 (d, ^3^*J*(P,P)=35.8 Hz), 269.6 ppm (d, ^3^*J*(P,P)=35.8 Hz); ^13^C NMR (75 MHz, CD_2_Cl_2_): *δ*=23.0 (s; CCH_2_*C*H_2_), 23.5 (s; CCH_2_*C*H_2_), 25.4 (dd, *J*(P,C)=4.2 Hz, 14.3 Hz; P=C*C*H_3_), 30.0 (d, *J*(P,C)=12.5 Hz; C*C*H_2_CH_2_), 30.4 (dd, *J*(P,C)=7.0, 20.7 Hz; C*C*H_2_CH_2_), 31.6 (s; *para*-*t*Bu), 32.8 (d, *J*(P,C)=7.0 Hz; CH_3_, *ortho*-*t*Bu), 33.1 (d, *J*(P,C)=6.9 Hz; CH_3_, *ortho*-*t*Bu), 35.4 (s; *para*-*C*(CH_3_)_3_), 38.2 (s; *ortho*-*C*(CH_3_)_3_), 38.5 (s; *ortho*-*C*(CH_3_)_3_), 122.2 (s; C5, pyridyl), 122.4 (s; CH, phenyl), 124.6 (d, *J*(P,C)=2.5 Hz; C3, pyridyl), 129.2 (d, *J*(P,C)=73.2 Hz; *ipso*-C, phenyl), 129.2 (d, *J*(P,C)=12.4 Hz; CH, phenyl), 131.5 (s; CH, Mes*), 131.5 (s; CH, Mes*), 132.1 (d, *J*(P,C)=3.0 Hz; phenyl), 136.2 (s; C4, pyridyl), 136.9 (d, *J*(P,C)=63.7 Hz; *ipso*-C, Mes*), 141.4 (d, *J*(P,C)=21.0 Hz; C14/C7), 142.2 (d, *J*(P,C)=21.1 Hz; C14/C7), 147.8 (dd, *J*(P,C)=18.3, 23.9 Hz; C13), 149.8 (s; pyridyl C6), 150.8 (s; Mes*), 152.9 (d, *J*(P,C)=16.8 Hz; C8), 153.7 (dd, *J*(P,C)=20.9, 5.4 Hz; pyridyl C2), 154.6 (s; Mes*), 174.0 ppm (dd, *J*(P,C)=8.2, 46.5 Hz; P=C); HRMS (ESI): *m*/*z* calcd for C_78_H_98_AgP_4_S_2_: 1359.51936; found: 1359.51927 [2 *M*+Ag]^+^.

**Phosphaalkene 6 b**: Phosphaalkene **4 b** (300 mg, 0.62 mmol) and [Cp_2_ZrCl_2_] (224 mg, 0.77 mmol) were added to a 100 mL Schlenk flask, and the two compounds were dried for 4 h. THF (20 mL) was added, and the yellow solution was cooled to −78 °C. *n*BuLi (0.50 mL, 1.25 mmol, 2.5 m in hexanes) was added, and the resulting red solution was stirred at this temperature for 1 h and then warmed to room temperature and left overnight. The following morning the solution was cooled to −78 °C, and PhPBr_2_ (0.18 mL, 0.9 mmol) was added. The red solution was warmed to room temperature and stirred for 24 h followed by a filtration through basic alumina. The alumina was rinsed with THF (3×20 mL) until the color of the eluent disappeared. The yellow-orange solution was concentrated, redissolved in CH_2_Cl_2_, and an excess amount of sulfur was added. The solution was stirred overnight at room temperature, filtered, and the solvent was removed under vacuum. Column chromatography was performed on silica using pure pentane to elute the remaining starting material and byproducts, and then the polarity was increased to 5 % Et_2_O in pentane. This gave **6 b** as an orange solid. Yield 74 mg, 0.12 mmol (19 %). ^1^H NMR (300 MHz, CDCl_2_): *δ*=1.24 (s, 9 H; *tert*-Bu), 1.29 (s, 9 H; *tert*-Bu), 1.40 (d, ^3^*J*(P,H)=1.9 Hz, 3 H; C*H*_3_), 1.45 (s, 9 H; *tert*-Bu), 1.87 (m, 4 H; C*H_2_*), 2.92 (m, 2 H; C*H_2_*), 3.08 (m, 2 H; C*H_2_*) 6.96 (dd, ^1^*J*(H,H)=3.8, 5.1 Hz, 1 H; H4, thienyl H4), 7.24 (d, ^1^*J*(H,H)=3.5 Hz, 1 H; thienyl, H5), 7.33 (d, ^1^*J*(H,H)=5.7 Hz, 1 H; thienyl, H3), 7.34 (s, 1 H; Mes*) 7.38 (s, 1 H; Mes*), 7.41 (m, 3 H; *m-*/*p*-H Ph), 7.87 ppm (ddd, *J*=1.5, 8.0, 13.9 Hz, 2 H; *ortho*-phenyl); ^31^P{^1^H} NMR (161.8 MHz, CD_2_Cl_2_): *δ*=51.7 (d, ^3^*J*(P,P)=36.7 Hz), 268.6 ppm (d, ^3^*J*(P,P)=36.7 Hz); ^13^C NMR (75 MHz, CDCl_2_): *δ*=23.1 (s; CCH_2_*C*H_2_), 23.5 (d, *J*(P,C)=2.3 Hz; CCH_2_*C*H_2_), 25.6 (dd, *J*(P,C)=14.3, 4.6 Hz; P=C*C*H_3_), 29.6 (d, *J*(P,C)=13.0 Hz; C*C*H_2_CH_2_), 30.5 (dd, *J*(P,C)=9.1, 13.3 Hz; C*C*H_2_CH_2_), 31.6 (s; CH_3_, *para*-*t*Bu), 32.8 (d, *J*(P,C)=7.0 Hz; CH_3_, *ortho*-*t*Bu), 33.1 (d, *J*(P,C)=7.1 Hz; CH_3_, *ortho*-*t*Bu), 35.4 (s; *para*-*C*(CH_3_)_3_), 38.2 (s; *ortho*-*C*(CH_3_)_3_), 38.4 (s; *ortho*-*C*(CH_3_)_3_), 122.3 (s; CH, phenyl), 127.2 (s; thienyl C5), 127.7 (s; thienyl C4), 128.2 (dd, *J*(P,C)=5.2, 1.9 Hz; thienyl C3), 129.2 (d, *J*(P,C)=72.7 Hz; CH, *ipso*-C, phenyl), 129.2 (d, *J*(P,C)=12.4 Hz; CH, phenyl), 131.4 (s; CH, Mes*), 131.6 (s; CH, Mes*), 132.3 (d, *J*(P,C)=3.0 Hz; phenyl), 135.5 (dd, *J*(P,C)=1.7, 16.8 Hz; thienyl, C2), 137.0 (d, *J*(P,C)=63.4 Hz; *ipso*-C, Mes*), 138.9 (d, *J*(P,C)=8.7 Hz; C_β_), 140.2 (dd, *J*(P,C)=20.2, 78.0 Hz; C_α_), 145.8 (dd, *J*(P,C)=6.6, 22.0 Hz; C_α_), 148.2 (dd, *J*(P,C)=18.8, 23.0 Hz; C_β_), 150.8 (s; *para*-C Mes*) 154.5 (s; *ortho*-C Mes*), 174.0 ppm (dd, *J*(P,C)=8.2, 46.3 Hz; P=C); HRMS (ESI): *m*/*z* 653.2567 [*M*+Na]^+^; calcd for C_38_H_48_NaP_2_S_2_ 653.25649.

Phosphaalkene **6 c** was prepared from **4 c** in 29 % isolated yield analogously to the procedure described for **6 b** (see the Supporting Information).

**Phosphaalkene 7**: [AuCl(tht)] (22 mg, 0.068 mmol) was added to PPA **5 a** (40 mg, 0.068 mmol) in CH_2_Cl_2_ (10 mL), and the reaction mixture turned immediately red. The mixture was stirred under argon for 1 h and the solvent was removed under vacuum. Recrystallization from CH_2_Cl_2_/CH_3_CN. Compound **7** was obtained as a red-orange solid. Yield: 55 mg, 0.067 mmol (98 %). ^1^H NMR (400 MHz, CD_2_Cl_2_): *δ*=1.23 (br s, 9 H; *tert*-Bu), 1.30 (s, 9 H; *tert*-Bu), 1.39 (s, 9 H; *tert*-Bu) 1.54 (d, ^3^*J*(P,H)=14.9 Hz, 3 H; CH_3_), 1.81 (m, 4 H; CCH_2_C*H_2_*), 2.94 (m, 1 H; CC*H_2_*CH_2_) 3.05 (m, 2 H; CC*H_2_*CH_2_), 3.23 (m, 1 H; CC*H_2_*CH_2_), 7.09 (m, 1 H; pyridyl, H5), 7.38 (m, 5 H; Mes*, Ph), 7.62 (m, 2 H; pyridyl H3 and/or H4 and/or phenyl), 7.75 (m, 2 H; pyridyl H3 and/or H4 and/or phenyl), 8.45 ppm (m, 1 H; pyridyl H6); ^31^P NMR (161.8 MHz, CD_2_Cl_2_): *δ*=38.3 (d, ^3^*J*(P,P)=75.6 Hz), 279.4 ppm (d, ^3^*J*(P,P)=75.6 Hz); ^13^C NMR (101 MHz, CDCl_3_): *δ*=22.3 (s; CCH_2_*C*H_2_), 22.8 (s; CCH_2_*C*H_2_), 26.4 (br m; P=C*C*H_3_), 29.7 (d, *J*(P,C)=9.9 Hz; C*C*H_2_CH_2_), 30.6 (br m; C*C*H_2_CH_2_), 31.4 (s; *para*-*t*Bu), 32.5 (br m; CH_3_, *ortho*-*t*Bu, both groups), 35.1 (s; *para*-*C*(CH_3_)_3_), 37.7 (s; *ortho*-*C*(CH_3_)_3_), 37.9 (s; *ortho*-*C*(CH_3_)_3_), 121.8 (s; C5, pyridyl), 122.0 (s; CH, phenyl), 123.8 (br m, 2 C; C3, pyridyl and *ipso*-C Ph), 129.2 (d, *J*(P,C)=13.0 Hz; CH, phenyl), 129.2 (s; CH, Mes*), 129.3 (s; CH, Mes*), 132.1 (s; phenyl), 134.6 (d, *J*(P,C)=15.3 Hz; C4, pyridyl), 136.4 (2 br m; *ipso*-C, Mes* and C7, C8, C13, or C14), 148.1 (br m; C7, C8, C13, or C14), 149.3 (br m, 2 C; C7, C8, C13, or C14), 150.7 (s; pyridyl C6), 153.6 (s; Mes*), 154.0 (br m under Mes* signals, pyridyl C2) 154.6 (s; Mes*), 174.0 ppm (dd, *J*(P,C)=9.2, 42.8 Hz; P=C); APCI/ASAP: *m*/*z* calcd for C_39_H_50_NClP_2_Au: 826.2760; found: 826.2767 [*M*+H]^+^; *m*/*z* calcd for C_39_H_50_NP_2_: 594.3413; found: 594.3414 [*M*−AuCl+H]^+^.

**Phosphaalkene 8**: [AuCl(tht)] (22 mg, 0.069 mmol) was added to PPA **5 a** (20 mg, 0.034 mmol) in CH_2_Cl_2_ (5 mL), and the reaction mixture turned immediately yellow to orange. The mixture was stirred under argon for 1 h, and the solvent was removed under vacuum. After recrystallization from CH_2_Cl_2_/CH_3_CN, compound **8** was obtained as an orange solid. Yield: 22 mg, 0.021 mmol (61 %). ^1^H NMR (400 MHz, CD_2_Cl_2_): *δ*=1.31 (s, 9 H; *tert*-Bu) 1.36 (s, 9 H; *tert*-Bu), 1.56 (d, ^3^*J*(P,H)=28.6 Hz, 3 H; CH_3_), 1.65 (s, 9 H; *tert*-Bu), 1.89 (m, 4 H; CCH_2_C*H_2_*), 2.70 (m, 1 H; CC*H_2_*CH_2_) 3.20 (m, 3 H; CC*H_2_*CH_2_), 7.14 (m, 1 H; pyridyl, H5), 7.43–7.60 (m, 5 H; Mes*, Ph), 7.60 (m, 1 H; pyridyl H3), 7.67 (m, 1 H; pyridyl H4), 7.82 (m, 2 H; phenyl), 8.45 ppm (m, 1 H; pyridyl H6); ^31^P NMR (161.8 MHz, CD_2_Cl_2_): *δ*=44.1 (d, ^3^*J*(P,P)=45.0 Hz), 199.0 (d, ^3^*J*(P,P)=45.0 Hz); ^13^C NMR (101 MHz, CD_2_Cl_2_): *δ*=22.6 (s; CCH_2_*C*H_2_), 22.8 (s; CCH_2_*C*H_2_), 27.4 (dd, *J*(P,C)=3.5, 7.4 Hz; P=C*C*H_3_), 29.7 (dd, *J*(P,C)=9.3, 63.2 Hz; C*C*H_2_CH_2_), 31.1 (s; C*C*H_2_CH_2_), 31.3 (s; *para*-*t*Bu), 34.2 (d, *J*(P,C)=1.3 Hz; CH_3_, *ortho*-*t*Bu), 34.3 (m; CH_3_, *ortho*-*t*Bu), 35.8 (s; *para*-*C*(CH_3_)_3_), 39.0 (s; *ortho*-*C*(CH_3_)_3_), 39.3 (s; *ortho*-*C*(CH_3_)_3_), 123.1 (s; C5, pyridyl), 123.7 (d, *J*(P,C)=23.0; C2, pyridyl), 123.7 (d, *J*(P,C)=23.0; C2, pyridyl), 124.0 (d, *J*(P,C)=5.9 Hz; C3 pyridyl or phenyl), 124.2 (d, *J*(P,C)=17.6 Hz; C3 pyridyl or phenyl), 124.2 (s; CH, phenyl), 125.0 (d, *J*(P,C)=60.6 Hz; *ipso*-C, phenyl), 130.2 (s; CH, Mes*), 130.3 (s; CH, Mes*), 137.3 (s; C4, pyridyl), 139.1 (dd, *J*(P,C)=5.8, 54.5 Hz; *ipso*-C, Mes*), 149.7 (s; pyridyl C6), 151.8 (d, *J*(P,C)=12.9 Hz; C7 or C8 or C13 or C14), 152.8–153.2 (3 m overlapping; C7 or C8 or C13 or C14), 155.2 (d, *J*(P,C)=2.4 Hz; Mes*), 157.0 (s; Mes*), 167.2 ppm (dd, *J*(P,C)=9.4, 72.2 Hz; P=C); APCI/ASAP: *m*/*z* calcd for C_39_H_50_NClP_2_Au: 826.2767; found: 826.2761 [*M*−AuCl+H]^+^; *m*/*z* calcd for C_39_H_50_NP_2_: 594.3413; found: 594.3414 [*M*−2 AuCl+H]^+^. The P–Au bond of the P=C is unstable.

## Supporting Information

As a service to our authors and readers, this journal provides supporting information supplied by the authors. Such materials are peer reviewed and may be re-organized for online delivery, but are not copy-edited or typeset. Technical support issues arising from supporting information (other than missing files) should be addressed to the authors.

miscellaneous_information

## References

[1a] Haley MM, Tykwinski RR (2006). Carbon-Rich Compounds.

[1b] Diederich F, Stang PJ, Tykwinski RR (2005). Acetylene Chemistry.

[1c] Tour JM (2003). Molecular Electronics, Commercial Insights, Chemistry, Devices, Architecture and Programming.

[2a] Müllen K, Scherf U (2006). Organic Light Emitting Devices: Synthesis Properties and Applications.

[2b] Usta H, Facchetti A, Marks TJ (2011). Acc. Chem. Res.

[2c] Cheng Y-J, Yang S-H, Hsu C-S (2009). Chem. Rev.

[2d] Zucchero AJ, McGrier PL, Bunz UHF (2009). Acc. Chem. Res.

[2e] Grimsdale AC, Leok Chan K, Martin RE, Jokisz PG, Holmes AB (2009). Chem. Rev.

[2f] Kivala M, Diederich Fo (2008). Acc. Chem. Res.

[2g] Frampton MJ, Anderson HL (2007). Angew. Chem.

[b01] (2007). Angew. Chem. Int. Ed.

[2h] Anthony JE (2006). Chem. Rev.

[2i] Nielsen MB, Diederich F (2005). Chem. Rev.

[2j] Gholami M, Tykwinski RR (2006). Chem. Rev.

[3] Ball P (2006). Nature.

[4a] Salzner U, Lagowski JB, Pickup PG, Poirier RA (1998). Synth. Met.

[4b] Cyrañski MK, Krygowski TM, Katritzky AR, Schleyer PvR (2002). J. Org. Chem.

[5a] Dillon KB, Mathey F, Nixon JF (1998). Phosphorus: The Carbon Copy. From Organophosphorus to Phospha-organic Chemistry.

[5b] Peruzzini M, Gonsalvi L (2011). Phosphorus Compounds: Advanced Tools in Catalysis and Material Sciences.

[5c] Quin LD (2000). A Guide to Organophosphorus Chemistry.

[6a] Geng X-L, Hu Q, Schäfer B, Ott S (2010). Org. Lett.

[6b] Geng X-L, Ott S (2009). Chem. Commun.

[6c] Geng X-L, Ott S (2011). Chem. Eur. J.

[6d] Öberg E, Geng X-L, Santoni M-P, Ott S (2011). Org. Biomol. Chem.

[6e] Öberg E, Schäfer B, Geng X-L, Pettersson J, Hu Q, Kritikos M, Rasmussen T, Ott S (2009). J. Org. Chem.

[6f] Schäfer B, Öberg E, Kritikos M, Ott S (2008). Angew. Chem.

[b02] (2008). Angew. Chem. Int. Ed.

[7a] Hissler M, Dyer PW, Reau R, Majoral J-P (2005). The Rise of Organophosphorus Derivatives in π-Conjugated Materials Chemistry New Aspects in Phosphorus Chemistry V, Vol. 250.

[7b] Wright VA, Gates DP (2002). Angew. Chem.

[b03] (2002). Angew. Chem. Int. Ed.

[7c] Bates JI, Dugal-Tessier J, Gates DP (2010). Dalton Trans.

[7d] Wright VA, Patrick BO, Schneider C, Gates DP (2006). J. Am. Chem. Soc.

[7e] Gates DP, Majoral J-P (2005). Expanding the Analogy Between P=C and C=C Bonds to Polymer Science: New Aspects in Phosphorus Chemistry V, Vol. 250.

[7f] Lejeune M, Grosshans P, Berclaz T, Sidorenkova H, Besnard C, Pattison P, Geoffroy M (2011). New J. Chem.

[7g] Smith RC, Protasiewicz JD (2004). Eur. J. Inorg. Chem.

[7h] Gudimetla VB, Ma L, Washington MP, Payton JL, Cather Simpson M, Protasiewicz JD (2010). Eur. J. Inorg. Chem.

[7i] Smith RC, Protasiewicz JD (2004). J. Am. Chem. Soc.

[7j] Baumgartner T, Réau R (2006). Chem. Rev.

[7k] Orthaber A, Herber RH, Pietschnig R (2012). J. Organomet. Chem.

[8a] Baumgartner T, Neumann T, Wirges B (2004). Angew. Chem.

[b04] (2004). Angew. Chem. Int. Ed.

[8b] Schaefer W, Schweig A, Mathey F (1976). J. Am. Chem. Soc.

[8c] Mattmann E, Mathey F, Sevin A, Frison G (2002). J. Org. Chem.

[8d] Mattmann E, Mercier F, Ricard L, Mathey F (2002). J. Org. Chem.

[8e] Nyulászi L, Holloczki O, Lescop C, Hissler M, Réau R (2006). Org. Biomol. Chem.

[8f] Delaere D, Pham-Tran N-N, Nguyen MT (2004). Chem. Phys. Lett.

[9a] Fadhel O, Gras M, Lemaitre N, Deborde V, Hissler M, Geffroy B, Réau R (2009). Adv. Mater.

[9b] Fadhel O, Benkö Z, Gras M, Deborde V, Joly D, Lescop C, Nyulászi L, Hissler M, Réau R (2010). Chem. Eur. J.

[9c] Fave C, Hissler M, Kárpáti T, Rault-Berthelot J, Deborde V, Toupet L, Nyulászi L, Réau R (2004). J. Am. Chem. Soc.

[10a] Ren Y, Kan WH, Henderson MA, Bomben PG, Berlinguette CP, Thangadurai V, Baumgartner T (2011). J. Am. Chem. Soc.

[10b] Ren Y, Kan WH, Thangadurai V, Baumgartner T (2012). Angew. Chem.

[b05] (2012). Angew. Chem. Int. Ed.

[10c] Graule Sb, Rudolph M, Vanthuyne N, Autschbach J, Roussel C, Crassous J, Réau Rg (2009). J. Am. Chem. Soc.

[10d] Chen H, Delaunay W, Yu L, Joly D, Wang Z, Li J, Wang Z, Lescop C, Tondelier D, Geffroy B, Duan Z, Hissler M, Mathey F, Réau R (2012). Angew. Chem.

[b06] (2012). Angew. Chem. Int. Ed.

[10e] Matano Y, Saito A, Fukushima T, Tokudome Y, Suzuki F, Sakamaki D, Kaji H, Ito A, Tanaka K, Imahori H (2011). Angew. Chem.

[b07] (2011). Angew. Chem. Int. Ed.

[10f] Nohra B, Graule S, Lescop C, Réau R (2006). J. Am. Chem. Soc.

[10g] Bruch A, Fukazawa A, Yamaguchi E, Yamaguchi S, Studer A (2011). Angew. Chem.

[b08] (2011). Angew. Chem. Int. Ed.

[10h] Bouit P-A, Escande A, Szűcs R, Szieberth D, Lescop C, Nyulászi L, Hissler M, Réau R (2012). J. Am. Chem. Soc.

[11a] Waschbuesch K, Le Floch P, Mathey F (1995). Bull. Soc. Chim. Fr.

[11b] Sava X, Mézailles N, Maigrot N, Nief F, Ricard L, Mathey F, Le Floch P (1999). Organometallics.

[12] Grundy J, Donnadieu B, Mathey F (2006). J. Am. Chem. Soc.

[13a] Fagan PJ, Nugent WA, Calabrese JC (1994). J. Am. Chem. Soc.

[13b] Fagan PJ, Nugent WA (1988). J. Am. Chem. Soc.

[14] Appell R, Casser C, Immenkeppel M (1985). Tetrahedron Lett.

[15a] Jun H, Angelici RJ (1993). Organometallics.

[15b] Jun H, Young VG, Angelici RJ (1994). Organometallics.

[15c] Romanenko VD, Sanchez M, Sarina TV, Mazières M-R, Wolf R (1992). Tetrahedron Lett.

[16] van der Sluis M, Klootwijk A, Wit JBM, Bickelhaupt F, Veldman N, Spek AL, Jolly PW (1997). J. Organomet. Chem.

[17] Köbrich G, Trapp H (1966). Chem. Ber.

[18] van der Sluis M, Wit JBM, Bickelhaupt F (1996). Organometallics.

[19] Orthaber A, Öberg E, Jane RT, Ott S (2012). Z. Anorg. Allg. Chem.

[20a] Turcheniuk KV, Rozhenko AB, Shevchenko IV (2011). Eur. J. Inorg. Chem.

[20b] Toyota K, Shimura K, Takahashi H, Yoshifuji M (1994). Chem. Lett.

[20c] Ruf SG, Dietz J, Regitz M (2000). Tetrahedron.

[20d] Mahnke J, Zanin A, du  Mont W-W, Ruthe F, Jones PG (1998). Z. Anorg. Allg. Chem.

[21] Hay C, Hissler M, Fischmeister C, Rault-Berthelot J, Toupet L, Nyulászi L, Réau R (2001). Chem. Eur. J.

[22] Su H-C, Fadhel O, Yang C-J, Cho T-Y, Fave C, Hissler M, Wu C-C, Réau R (2005). J. Am. Chem. Soc.

[23] Benkõ Z, Gudat D, Nyulászi L (2008). Chem. Eur. J.

[25] Sauthier M, Leca F, Toupet L, Réau R (2002). Organometallics.

