# Supplementary material for: Phosphorus Centers of Different Hybridization in Phosphaalkene-Substituted Phospholes
Source: Chemistry. 2014 May 30;20(27):8421–32. doi: 10.1002/chem.201402406 (PMC4506554; doi:10.1002/chem.201402406)
Supplement: Supplementary file 1 — miscellaneous_information [file chem0020-8421-sd1.pdf]

CHEMISTRY
A European Journal

Supporting Information

© Copyright Wiley-VCH Verlag GmbH & Co. KGaA, 69451 Weinheim, 2014

Phosphorus Centers of Different Hybridization in Phosphaalkene-Substituted Phospholes

Elisabet Öberg,[a] Andreas Orthaber,[a] Christophe Lescop,[b] Régis Réau,*[b] Muriel Hissler,*[b] and Sascha Ott*[a]

chem_201402406_sm_miscellaneous_information.pdf

Materials and general methods: Chemicals were purchased from Sigma-Aldrich, VWR, ABCR and Fisher Scientific and used as received. THF and Et_2O were distilled from sodium/benzophenone. CH_2Cl_2 was distilled from calcium hydride or freshly purified using MBRAUN SPS-800 drying columns. All reactions were performed under an inert atmosphere of N_2 or Ar using standard Schlenk techniques. Column chromatography was performed on Merck silica gel SI-60Å (35-70 or 0.063-0.200 mm), or on basic alumina (Aldrich type 5016A, 150 mesh, 58 Å).

NMR Spectroscopy: ^{1}H-NMR spectra were recorded at ambient temperature on a JEOL Eclipse + 400 MHz spectrometer (operating at 399.8 MHz), Varian Mercury Plus 300 MHz spectrometer (operating at 300.03 MHz) or Bruker AM300, AM400, AM500. Chemical shifts are given in ppm and referenced internally to the residual solvent signal ($CHCl_3$, δ_H = 7.26 ppm or CH_2Cl_2, δ_H = 5.32 ppm). ^{13}C-NMR spectra were recorded on the same instrument (100.5 MHz respectively 75.4 MHz) and were also referenced internally to the residual solvent signal ($CHCl_3$, δ_c = 77.1 ppm, central signal, CH_2Cl_2, δ_c = 54.00 ppm). ^{31}P-{^{1}H}-NMR spectra were recorded on JEOL Eclipse + 400 MHz spectrometer (operating at 161.8 MHz), referenced externally to 85% H_3PO_4 (aq).

UV/Vis Spectroscopy: UV/Vis was performed using a Varian Cary 50 instrument or in a Varian Cary 5000 instrument at room temperature. The measurements were performed in 1x1 cm^2 optical quartz cells as solutions in CH_2Cl_2.
Mass Spectrometry: Low resolution mass spectrometry was performed on a Thermo LCQ Deca XP Max with direct injection electrospray ionization (ESI). A small amount of the compounds were dissolved in a solution of ~2.5 mM $AgBF_4$ in MeOH. High-resolution mass spectrometry (HRMS) were performed on a high-resolution and FTMS+pNSI mass spectrometer (orbitrapXL) or on a MicroTOF spectrometer with ESI core at University of Muenster or on a Varian MAT 311, Waters Q-TOF 2 or ZabSpec TOF Micromass instrument at CRMPO of Rennes 1. MALDI-MS spectra were obtained in Ditranol matrix using a nitrogen laser accumulating 50 laser shots and a Bruker Microflex LT MALDI-TOF mass spectrometer.

Phosphaalkene 2

Phosphaalkene **2** was prepared in analogy to the procedure for (Z)-[Bromo(trimethylsilyl)methylene](2,4,6-tri-*tert*-butylphenyl)phosphane reported Bickelhaupt.[1] Mes*P=C(Br)$_2$ (2.6 g, 5.8 mmol) was dissolved in 100 ml THF, 25 ml Et_2O and 25 ml pentane. The mixture was cooled to -120 °C with dry ice and N_2(l) in a cooling bath containing the 4:1:1 THF:Et_2O:pentane or a bath containing only pentane. n-BuLi (2.5 M, 2.3 ml, 5.80 mmol) was added dropwise and the yellow to orange reaction mixture was kept at -120 °C for 30 min. MeI (0.72 ml, 11.6 mmol) was added dropwise, the color of the reaction mixture became slightly paler and it was kept at -120 °C for 30 min before warming up to r.t. for 2 h. 20 ml of a 1:1 V:V mixture of (28 % NH_3 in water:EtOH was added and the solution was stirred for 30 min. After that the organic phase was washed with a 2 x 50 ml of a saturated aqueous solution of $NaHCO_3$ and 2 x 50 ml of a saturated aqueous solution of NH_4Cl. The organic phase was dried with $MgSO_4$ and filtered. Solvent removed in vacuo. Column chromatography in pentane yielded a white solid. Yield 2.07 g, 5.40 mmol (93 %). Recrystallization from CH_2Cl_2/acetonitrile yields white crystals. ^{1}H-NMR (399.8 MHz, $CDCl_3$): δ 1.36 (s, 9H, p-*tert*-butyl), 1.50 (s, 18H, o-*tert*-butyl), 2.68 (d, J_{PH} = 24.4 Hz, 3H, CH_3) 7.43 (s, 2H, Ar). ^{31}P{^{1}H}-NMR (161.8 MHz, $CDCl_3$): δ 242.4. ^{13}C-NMR(100.5 MHz, $CDCl_3$): 31.3 (d, J_{PC}=35.2Hz, CH_3), 31.4 (s, p-C($\underline{C}H_3$)$_3$), 32.6 (s, o-C($\underline{C}H_3$)$_3$), 32.7 (s, o-C($\underline{C}H_3$)$_3$), 35.0 (s, p-$\underline{C}$(CH_3)$_3$), 37.8 (s, o-C($\underline{C}H_3$)$_3$), 122.0 (s, meta-Ar), 138.7 (d, P-C, J_{PC} =52.2 Hz), 150.7 (s, para-Ar), 152.9 (s, orto-Ar), 161.2 (d, P-C, J_{PC} = 57.0 Hz). ESI MS: *m/z* [2M+Ag]$^+$ 872.95 (100), [MH+Ag]$^+$ 490.96 (50).

Phosphaalkene 4b

Phosphaalkene **3** (500 mg, 1.22 mmol), $Pd(PPh_3)_2Cl_2$ (43 mg, 0.062 mmol) and CuI (12 mg, 0.062 mmol) were dried. Deaerated Et_3N, 20 ml, was added to the reagents and finally 2-iodothiophene (0.20 ml, 1.8 mmol). The yellow solution turned to a suspension and after stirring overnight the color had changed to brown. The solvent was removed in vacuo. The product was extracted from the solid by pentane. Column chromatography in pentane gave the product as a white solid. Yield: 575 mg, 1.17 mmol (96 %). Recrystallization from CH_3CN/CH_2Cl_2 gives colorless crystals. ^{1}H-NMR (400 MHz, $CDCl_3$): δ = 1.29 (d, 3H, C*H₃*, $^3J_{PH}$ = 13.7 Hz), 1.33 (s, 9H, p-*tert*-Bu), 1.48 (s, 18H, o-*tert*-Bu), 1.75 (m, 4H, CH_2), 2.49 (m, 4H, CH_2), 6.93 (m, 1H, H4, thienyl), 7.12 (m, 1H, H3 thienyl), 7.17 (m, 1H, thienyl H5), 7.41 (s, 2H, Mes*). ^{31}P-{^{1}H}-NMR (161.8 MHz, $CDCl_3$): δ = 274.8 ppm. ^{13}C-NMR (100 MHz, $CDCl_3$): δ = 19.3 (s, , thienyl-C≡CCH_2*C*H_2), 19.9 (d, , C≡CCH_2*C*H_2, $^4J_{PC}$ = 5.0 Hz), 25.4 (d, CH_3, $^2J_{PC}$ = 14.4 Hz), 27.8 (s, thieny-C≡C*C*H_2CH_2), 28.0 (d, C≡C*C*H_2CH_2, $^5J_{PC}$ = 4.5 Hz), 31.3 (s, p-C(*C*H_3)$_3$), 32.6 (d, o-C(*C*H_3)$_3$, $^4J_{PC}$ = 6.8 Hz), 35.0 (s, o-*C*(CH_3)$_3$), 37.9 (s, p-*C*(CH_3)$_3$), 73.9 (s, C≡*C*-thienyl), 85.8 (d, P=C-C≡*C*, $^3J_{PC}$ = 28.8 Hz), 90.6 (s, *C*≡C-thienyl), 97.5 (d, P=C-*C*≡C, $^2J_{PC}$ = 18.8 Hz), 121.6 (s, meta-Ph), 124.1 (s, C2, thienyl), 125.9 (s, C4, thienyl), 126.7 (s, C5, thienyl), 131.0 (s, C3, thienyl),136.1 (d, $^1J_{PC}$ = 60.3 Hz, P-C), 150.3 (s, para-Ar), 153.7 (s, orto-Ar), 162.1 (d, P=C, $^1J_{PC}$ =32.3 Hz, P=C). ESI MS: *m/z* $[M+Ag]^+$ 597.4 (100). HR-MS (ESI): m/z 513.2720 $[M+Na]^+$; calcd for $C_{32}H_{43}NaPS$ 513.27208. Anal. Calcd. for $[C_{32}H_{43}Ps+CH_3CN]$ C, 76.79; H, 8.72. Found C, 76.49; H, 8.69. The assignment was based on the assignment of 1-(2-thienyl)octa-1,7-diyne.[2]

Phosphaalkene 4c

Phosphaalkene **3** (800 mg, 1.96 mmol), $Pd(PPh_3)_2Cl_2$ (140 mg, 0.20 mmol) and CuI (39 mg, 0.20 mmol) were dried. Deaerated Et_2NH 20 ml was added to the reagents and finally iodobenzene (1.11 ml, 10.0 mmol). The yellow solution turned to a suspension and after stirring overnight the color had changed to brown. The solvent was removed in vacuo. The product was extracted from the solid by pentane. Column chromatography in pentane gave the product as a white solid. Yield: 513 mg, 1.06 mmol (54 %). Recrystallization from CH_3CN/CH_2Cl_2 gives colorless crystals. ^{1}H-NMR (400 MHz, $CDCl_3$): δ = 1.28 (d, 3H, CH_3, $^3J_{PH}$ = 13.6 Hz), 1.32 (s, 9H, p-*tert*-Bu), 1.48 (s, 18H, o-*tert*-Bu), 1.76 (m, 4H, CH_2), 2.48 (m, 4H, CH_2), 7.25 (m, 3H, Ph), 7.38 (m, 2H, Ph) 7.39 (br. s, 2H, Mes*). ^{31}P-{^{1}H}-NMR (161.8 MHz, $CDCl_3$): δ = 275.3 ppm. ^{13}C-NMR (100 MHz, $CDCl_3$): δ = 19.0 (s, Ph-C≡CCH_2*C*H_2), 19.9 (d, C≡CCH_2*C*H_2, $^4J_{PC}$ = 3.0 Hz), 25.4 (d, CH_3, $^2J_{PC}$ = 14.4 Hz), 28.0 (s, Ph-C≡C*C*H_2CH_2), 29.6 (s, C≡C*C*H_2CH_2), 31.3 (s, p-C(*C*H_3)$_3$), 32.6 (d, o-C(*C*H_3)$_3$, $^4J_{PC}$ = 6.0 Hz), 35.0 (s, o-*C*(CH_3)$_3$), 37.9 (s, p-*C*(CH_3)$_3$), 80.9 (s, C≡C-Ph), 85.8 (d, P=C-C≡C, J_{PC} = 29.0 Hz), 89.9 (s, C≡C-Ph), 97.6 (d, P=C-C≡C, J_{PC} = 18.5 Hz), 121.6 (s, meta-Mes*), 124.0 (s, ipso-Ph), 127.5 (s, para-Ph), 128.2 (s, meta-Ph), 131.6 (s, ortho-Ph), 136.2 (d, $^1J_{PC}$ = 59.9 Hz, P-C), 150.3 (s, para-Mes*), 153.7 (s, orto-Mes*), 162.1 (d, P=C, $^1J_{PC}$ =32.6 Hz, P=C). HR-MS (ESI): m/z 591.22792 $[M+Ag]^+$; calcd for $C_{34}H_{45}PAg$ 519.23043. Anal. Calcd. for $C_{34}H_{45}P$; C, 84.25; H, 9.36. Found C, 83.95; H, 9.33.

PPA 5b: Phosphaalkene **4b** (200 mg, 0.41 mmol) and Cp_2ZrCl_2 (149 mg, 0.51 mmol) were added to a 100 ml Schlenk flask and the two compounds were dried for 4 h. THF (15 ml) was added and the yellow solution was cooled to -78 °C. n-BuLi (0.33 ml, 0.82 mmol, 2.5M in hexanes) was added and the resulting red solution was stirred at this temperature for 1h and then warmed to room temperature and left overnight. Following morning the solution was cooled to -78 °C and $PhPBr_2$ (0.12 ml, 0.60 mmol) was added. The red solution was warmed to room temperature and stirred for 24 h followed by a filtration through basic alumina. The alumina was rinsed with THF (3 x 15 ml) until the colour of the eluent disappeared. The yellow-orange solution was concentrated yielding (**5b**) as a yellow solid.

Rapid column chromatography on silica with pentane gave (**5b**) as a yellow solid. Yield: 21 mg, 0.090 mmol (21 %). ^{1}H-NMR (400 MHz, $CDCl_3$): δ = 1.22 (s, 9H, *tert*-Bu), 1.29 (s, 9H, *tert*-Bu), 1.37 (s, 9H, *tert*-Bu), 1.49 (d, 3H, CH_3, $^{3}J_{PH}$ = 13.5 Hz), 1.83 (m, 4H, CH_2), 2.82 (m, 2H, CH_2), 3.01 (m, 2H, CH_2) 6.93 (ddd, 1H, $^{5}J_{PH}$ =1.1Hz, $^{3}J_{HH}$ =3.7Hz, $^{3}J_{HH}$ =4.9 Hz, H4, thienyl), 7.04 (d broad, 1H, $^{3}J_{HH}$ =3.7Hz H5, thienyl), 7.18 (m, 1H, H3, thienyl), 7.25 (m, 3H, *m*-/*p*-H Ph) 7.34 (s, 1H, Mes*) 7.36 (s, 1H, Mes*), 7.42 (ddd, 2H, $^{3}J_{HH}$ =1.4Hz, 8.2 Hz, $^{3}J_{PH}$ = 8.2Hz, *o*-H Ph). ^{31}P-{^{1}H}-NMR (161.8 MHz, CD_2Cl_2): δ = 10.5 (d, $^{3}J_{PP}$ =92.8 Hz), 249.4 (d, $^{3}J_{PP}$ = 92.8 Hz). ^{13}C-NMR (100 MHz, CD_2Cl_2): δ = 23.1 (s, CCH_2CH_2), 23.4 (d, J_{PC}=1.9 Hz, , CCH_2CH_2), 25.9 (dd, J_{PC} = 9.8 Hz, 14.5 Hz, P=CCH_3), 29.7 (s, CCH_2CH_2, C9), 30.5 (dd, J_{PC} = 1.6 Hz, 5.6 Hz), 31.1 (s, CH_3, para-*t*Bu), 31.6 (d, J_{PC} = 6.6 Hz, CH_3, orto-*t*Bu), 32.3 (d, J_{PC} = 7.5 Hz, CH_3, orto-*t*Bu), 34.9 (s, para-$C(CH_3)_3$), 37.6 (s, orto-$C(CH_3)_3$), 37.9 (s, orto-$C(CH_3)_3$), 121.7 (d, J_{PC} =13.0 Hz, CH, phenyl), 124.6 (dd, J_{PC} = 1.8 Hz, 2.2 Hz, thienyl, C5), 125.0 (dd, J_{PC} = 2.5 Hz, 9.8 Hz, thienyl, C3), 127.3 (s, thienyl, C4), 128.5 (d, J_{PC} = 8.7 Hz, CH, phenyl), 129.8 (d, J_{PC} = 1.8 Hz, CH, phenyl), 132.3 (d, J_{PC} = 11.0 Hz, ipso-C, phenyl), 134.7 (s, CH, Mes*), 134.9 (s, CH, Mes*), 136.5 (d, J_{PC} = 14.4 Hz, thienyl, C2), 138.1 (dd, J_{PC} = 6.8 Hz, 61.7 Hz, ipso-C, Mes*), 140.0 (dd, J_{PC} = 2.8 Hz, 22.1 Hz, C_β or $C_{\beta'}$), 144.6 (dd, J_{PC} = 10.3 Hz, 20.4 Hz, C_α or $C_{\alpha'}$), 145.0 (dd, J_{PC} = 5.1 Hz, 9.4 Hz, C_α or $C_{\alpha'}$), 149.3 (dd, J_{PC} = 1.5 Hz, 23.6 Hz, C_β or $C_{\beta'}$) 149.9 (s, para-C, Mes*), 153.6 (s, s, orto-C, Mes*), 154.2 (d, J_{PC} = 1.8 Hz, s, orto-C, Mes*), 178.8 (dd, J_{PC} = 16.5 Hz, 42.8 Hz, P=C). ESI MS: *m/z* $[M+Ag]^+$ 705.3 HR-MS (ESI): m/z 599.30108 $[M+H]^+$; calcd for $C_{38}H_{48}P_2SH$ 599.30247

PPA 6c: Phosphaalkene **4c** (250 mg, 0.52 mmol) and Cp_2ZrCl_2 (152 mg, 0.52 mmol) were added to a 100 ml Schlenk flask and the two compounds were dried for 10 h. THF (25 ml) was added and the colorless solution was cooled to -78 °C. n-BuLi (0.44 ml, 1.10 mmol, 2.5 M in hexanes) was added and the resulting red solution was stirred at this temperature for 1h and then warmed to room temperature and left overnight. Following morning the solution was cooled to -78 °C and $PhPBr_2$ (0.13 ml, 0.65 mmol) was added. The red solution was warmed to room temperature and stirred for 24h at room temperature followed by a filtration through basic alumina. The alumina was rinsed with THF (3 x 20 ml) until the color of the eluent disappeared. The yellow-orange solution was concentrated and ^{31}P-{^{1}H}-NMR showed the product **5c**; ^{31}P-{^{1}H}-NMR (161.8 MHz, CD_2Cl_2): δ = 12.2 (d, $^{3}J_{PP}$ = 90.3 Hz), 246.2 (d, $^{3}J_{PP}$ = 90.3 Hz). The mixture was redissolved in CH_2Cl_2 and an excess of sulfur was added. Stirred overnight at room temperature. Solvent was removed in vacuo. The mixture was subjected to column chromatography on silica using pure pentane to elute remaining starting material and by-products and then increasing the polarity to 5 % Et_2O in pentane. This gave PPA **6c** as a yellow solid. Yield: 95 mg, 0.15 mmol (29 %). ^{1}H-NMR (400 MHz, CD_2Cl_2): δ = 1.23 (s, 9H, *tert*-Bu), 1.29 (s, 9H, *tert*-Bu), 1.35 (d, 3H, CH_3, $^{3}J_{PH}$ = 17.8 Hz), 1.45 (s, 9H, *tert*-Bu), 1.82 (m, 4H, CH_2), 2.71 (m, 2H, CH_2), 3.08 (m, 2H, CH_2) 7.15 (m, 2H, Ph), 7.24 (m, 3H, Ph), 7.34 (s, 1H, Mes*) 7.38 (s, 1H, Mes*), 7.43 (m, 3H, Ph), 7.85 (m, 2H). ^{31}P-{^{1}H}-NMR (161.8 MHz, CD_2Cl_2): δ = 53.4 (d, $^{3}J_{PP}$ = 35.7 Hz), 267.8 (d, $^{3}J_{PP}$ =35.7 Hz). ^{13}C-NMR (75 MHz, $CDCl_2$): δ = 23.0 (s, CCH_2CH_2), 23.6 (d, J_{PC} = 2.1 Hz, CCH_2CH_2), 25.2 (dd, J_{PC} = 14.4 Hz, J_{PC} = 4.4 Hz, P=CCH_3), 28.6 (d, J_{PC} = 13.0 Hz, CCH_2CH_2), 30.2 (m, CCH_2CH_2), 31.6 (s,CH_3, para-*t*Bu), 32.8 (d, J_{PC} = 7.1 Hz, CH_3, orto-*t*Bu), 33.1 (d, J_{PC} = 7.1 Hz, CH_3, orto-*t*Bu), 35.4 (s, para-$C(CH_3)_3$), 38.2 (s, orto-$C(CH_3)_3$), 38.4 (s, orto-$C(CH_3)_3$), 122.3 (s, CH, phenyl), 128.2 (s, CH, phenyl) 128.7 (s, CH, phenyl), 129.2 (d, J_{PC} = 12.3 Hz, C-ipso, phenyl), 129.5 (dd, J_{PC} = 1.4 Hz, 4.8 Hz, phenyl), 131.6 (s, CH, Mes*),131.7 (s, CH, Mes*), 132.1 (d, J_{PC} = 3.0 Hz, phenyl), 133.4 (d, J_{PC} =11.7 Hz, phenyl), 134.9 (dd, J_{PC} =6.9 Hz, J_{PC} = 79.4 Hz, C_β or $C_{\beta'}$), 137.0 (d, J_{PC} = 63.5 Hz, ipso-C Mes*), 140.5 (dd, J_{PC} = 20.7 Hz, J_{PC} =76.0 Hz, C_α or $C_{\alpha'}$), 147.4 (dd, J_{PC} = 18.7 Hz, J_{PC} = 24.2 Hz, C_α or $C_{\alpha'}$), 149.3 (dd, J_{PC} = 5.8 Hz, J_{PC} = 22.8 Hz, C_β or C_β), 150.8 (s, para-C Mes*) 154.5 (s, orto-C Mes*), 174.3 (dd, J_{PC} = 8.2 Hz, J_{PC} = 46.2 Hz, P=C). HR-MS (ESI): m/z

1271.61191 [2M+Na]$^+$; calcd for $C_{80}H_{100}P_4S_2Na$ 1271.61091; m/z 647.30046 [M+Na]$^+$; calcd for $C_{40}H_{50}P_2SNa$ 647.30007

Crystallographic Studies: All measurements were performed using graphite-monochromatized Mo-*K*α radiation at 100 K with a Bruker Smart APEX-II (Uppsala University). Single crystal data collection of **6b** and **7** were performed at 150 K with a Bruker AXS APEX-II (Centre de Diffractométrie, Université de Rennes 1) France). Data integration and absorption correction was carried out with SAINT and SADABS, respectively.[3, 4] Structures were solved by direct methods (SHELXS-97) and refined by full-matrix least squares techniques against *F*2 (SHELXL-97)[5] using WinGX.[6] Graphical representations are prepared with ORTEP for Windows [7] and POV-Ray. The non-hydrogen atoms were refined with anisotropic displacement parameters. The hydrogen atoms of the phenyl rings were put at the external bisector of the C–C–C angle at a C–H distance of 0.95 Å. The hydrogen atoms of the methyl groups were refined with common isotropic displacement parameters for the H atoms of the same group and idealized geometry with tetrahedral angles, enabling rotation around the X–C bond, and C–H distances of 0.98 Å. CCDC 987053-987061contain supplementary crystallographic data for these structures. These data can be obtained free of charge from The Cambridge Crystallographic Data Centre via www.ccdc.cam.ac.uk/data_request/cif.

Phosphaalkene 3

Single crystals suitable for X-ray diffraction were obtained by slow evaporation of a CH_2Cl_2/CH_3CN solution of **3** as colorless plates. Compound **3** crystallizes in the monoclinic space group *P2(1)/c* (No. 14), $C_{28}H_{4o}P$, M = 407.57 g mol^{-1}, crystal dimensions 0.30 x 0.22 x 0.16 mm, a = 9.964(3) Å, b = 16.993(4) Å, c = 15.737(4) Å, β = 104.503(4)°, V = 2579.5(11) Å^3, Z = 2, $2\Theta_{max}$=55.72°, ρ = 1.049 g*cm^{-1}, $\mu(MoK_\alpha)$ = 0.117 mm^{-1}, F_{000} = 892, -13 ≤ h ≤ 13, -22≤ k ≤ 22, -20 ≤ l ≤ 20, 29165 reflections measured, 6109 unique (R_{int} = 0.0567) R1 = 0.0713 (I>2.0σ(I)), wR2 = 0.1991 (all data), GooF = 1.023, 290 parameters, 60 restraints.

Phosphaalkene 4b

Single crystals suitable for X-ray diffraction were obtained by slow evaporation of a CH_2Cl_2/CH_3CN solution of **4b** as colorless plates. Compound **4b** crystallizes in the triclinic space group *P-1* (No. 2), $C_{32}H_{43}PS$, M = 490.69g mol^{-1}, crystal dimensions 0.12 x 0.32 x 0.50 mm, a = 9.7441(4)Å, b = 9.9858(3)Å, c = 15.0447(5)Å, α = 91.634(2)°, β = 92.718(2)°, γ = 101.040(2)°, V = 1434.13(9)Å^3, Z = 2, $2\Theta_{max}$=56.98°, ρ = 1.1364(1)g*cm^{-1}, $\mu(MoK_\alpha)$ = 0.186 mm^{-1}, F_{000} = 532, -11 ≤ h ≤ 12, -12≤ k ≤ 12, -17 ≤ l ≤ 18, 21418 reflections measured, 5912 unique (R_{int} = 0.0616) R1 = 0.0753 (I>2.0σ(I)), wR2 = 0.2212 (all data), GooF = 1.065, 308 parameters, no restraints.

Phosphaalkene 4c

Single crystals suitable for X-ray diffraction were obtained by slow evaporation of a CH_2Cl_2/CH_3CN solution of **4c** at room temperature as colorless needles. Compound **4c** crystallizes in the triclinic space group *P-1*(No. 2), $C_{34}H_{45}P$, M = 484.67g mol^{-1}, crystal dimensions 0.13 x 0.18 x 0.3 mm, a = 9.7764(4)Å, b = 10.0297(5)Å, c = 15.2935(6)Å, α = 91.679(2)°, β = 93.271(3)°, γ = 101.101(2)°, V = 1467.90(11)Å^3, Z = 2, $2\Theta_{max}$=56.24°, ρ = 1.097g*cm^{-1}, $\mu(MoK_\alpha)$ = 0.113mm^{-1}, F_{000} = 528, -12 ≤ h ≤ 12, -13 ≤ k ≤ 13, -20 ≤ l ≤ 20, 25575 reflections measured, 7090 unique (R_{int} = 0.0695) R1 = 0.0514 (I>2.0σ(I)), wR2 = 0.1281(all data), GooF = 1.031, 357 parameters, 14 restraints. The flexible carbon atoms (C8-C15) show a disorder over two sites (*sof* 0.75 : 0.25) and were refined anisotropically with equal ADPs for each atom pair. Further restraints on distances and angles (SAME) have been applied to assure a geometrically meaningful refinement.

PPA 5a

Single crystals suitable for X-ray diffraction were obtained by slow evaporation of a solution of **5a** in CH_2Cl_2/CH_3CN at room temperature as yellow needles. Compound **5a** crystallizes in the monoclinic

space group $P2_1$/n (No. 14), $C_{39}H_{49}NP_2$, M = 593.73 g mol^{-1}, a = 13.2230(12) Å, b = 6.2038(6)Å, c = 43.674(4) Å, V = 3576.7(6) $Å^3$, Z = 4, $2\Theta_{max}$=54.54°, ρ = 1.103 g*cm^{-1}, μ(MoK_α) = 0.147 mm^{-1}, F_{000} = 1280, -16 ≤ h ≤ 16, -7 ≤ k ≤ 7, -56 ≤ l ≤ 55, 53892 reflections measured, 7946 unique (R_{int} = 0.0683) R1 = 0.0491 (I>2.0σ(I)), wR2 = 0.1737 (all data), GooF = 0.635, 391 parameters, 0 restraints. In order to improve the model to the data the contributions from disordered solvent molecules were eliminated with the PLATON/SQUEEZE algorithm. SQUEEZE estimated a total solvent accessible void of 334.9 $Å^3$. A total count of 84 electron fits two molecules of pentane, being used during the crystallization.[8]

PPA 6a

Single crystals suitable for X-ray diffraction were obtained by slow evaporation of a solution of **6a** in CH_2Cl_2/CH_3CN at room temperature as orange plates. Compound **6a** crystallizes in the orthorhombic space group *Pna*2₁ (No. 33), $C_{39}H_{49}NP_2S$, M = 625.80 g mol^{-1}, crystal dimensions 0.12 x 0.3 x 0.5 mm, a = 12.2123(3) Å, b = 22.2563(6)Å, c = 12.6685(3) Å, V = 3443.31(15) $Å^3$, Z = 4, $2\Theta_{max}$=54.3°, ρ = 1.207 g*cm^{-1}, μ(MoK_α) = 0.215 mm^{-1}, F_{000} = 1344, -15 ≤ h ≤ 15, -28 ≤ k ≤ 27, -15 ≤ l ≤ 16, 23892 reflections measured, 6995 unique (R_{int} = 0.0687) R1 = 0.0428 (I>2.0σ(I)), wR2 = 0.0952 (all data), GooF = 1.016, 402 parameters, 4 restraints.

PPA 6b

Single crystals suitable for X-ray diffraction were obtained by slow evaporation of a solution of **6b** in CH_2Cl_2/CH_3CN at room temperature as orange plates. Compound **6b** crystallizes in the monoclinic space group $P2_1$/n (No. 14), $C_{38}H_{48}P_2S_2$, M = 630.82 g mol^{-1}, a = 9.179(1) Å, b = 41.916(2)Å, c = 9.659(1) Å, α = 90°, β = 105.177(3)°, γ = 90°, V = 3586.7(6) $Å^3$, Z = 4, Θ_{max}=26.390°, ρ = 1.168 g*cm^{-1}, μ(MoK_α) = 0.262 mm^{-1}, F_{000} = 1352.0, -11 ≤ h ≤ 9, -41 ≤ k ≤ 52, -12 ≤ l ≤ 12, 7305 reflections measured, 5507 unique (R_{int} = 0.0695) R1 = 0.0457 (I>2.0σ(I)), wR2 = 0.1281 (all data), GooF = 1.048, 379 parameters, 0 restraints.

PPA 6c

Single crystals suitable for X-ray diffraction were obtained by slow evaporation of a solution of **6c** in CH_2Cl_2/CH_3CN at room temperature. Compound **6c** crystallizes in the monoclinic space group $P2_1$/n (No. 14), $C_{40}H_{50}P_2S$, M = 624.80 g mol^{-1}, a = 9.1028(3) Å, b = 15.77006(5)Å, c = 25.1978(8) Å, α = 90°, β = 93.780(2)°, γ = 90°, V = 3609.4(2) $Å^3$, Z = 4, Θ_{max}=29.69°, ρ = 1.150 g*cm^{-1}, μ(MoK_α) = 0.204 mm^{-1}, F_{000} = 1344, -12 ≤ h ≤ 12, -21 ≤ k ≤ 21, -35 ≤ l ≤ 34, 10213 reflections measured, 8396 unique (R_{int} = 0.0678) R1 = 0.0378 (I>2.0σ(I)), wR2 = 0.1007 (all data), GooF = 1.046, 440 parameters, 15 restraints. The phenyl atoms (C13-C15) of the Mes* and the *para tert*-Bu group (C21-C24) show a disorder over two sites (*sof* 0.51 : 0.49) and were refined anisotropically with equal ADPs for each atom pair, when necessary. Further restraints on distances and angles (SAME) have been applied to assure a geometrically meaningful refinement.

PPA 7

Single crystals suitable for X-ray diffraction were obtained by slow evaporation of a solution of **7** in CH_2Cl_2/CH_3CN at room temperature. Compound **7** crystallizes in the monoclinic space group $P2_1$/a.
(No. 14), $C_{39}H_{49}Au_1Cl_1N_1P_2$, M = 826.15 g mol^{-1}, a = 11.482(5) Å, b = 26.013(5) Å, c = 12.846(5) Å, α = 90°, β = 92.974(5)°, γ = 90°, V = 3832(2) $Å^3$, Z = 4, Θ_{max}=27.54°, ρ = 1.432 g*cm^{-1}, μ(MoK_α) = 4.019 mm^{-1}, F_{000} = 1664, -9 ≤ h ≤ 14, -20 ≤ k ≤ 33, -16 ≤ l ≤ 16, 17346 reflections measured, 8684 unique (R_{int} = 0.0619) R1 = 0.0560 (I>2.0σ(I)), wR2 = 0.1518 (all data), GooF = 0.972, 397 parameters, 0 restraints. Due to non-resolvable residual electron density the data were treated using the Squeeze algorithm.[8]

PPA 8

Single crystals suitable for X-ray diffraction were obtained by slow evaporation of a solution of **8** in CH_2Cl_2/CH_3CN at room temperature. Compound **8** crystallizes in the triclinic space group *P-1* (No. 2) as a diacetonitrile solvate. $C_{43}H_{55}Au_2Cl_2N_3P_2$, M = 1140.67 g mol^{-1}, a = 9.9741(10) Å, b = 12.0843(13) Å, c = 19.712(3) Å, α = 97.751(9)°, β = 98.215(9)°, γ = 113.281(6)°, V = 2111.7(4) $Å^3$, Z = 2,

Θ_{max}=27.05°, ρ = 1.794 g*cm^{-1}, μ(MoK_α) = 7.176, F_{000} = 1112, -12 ≤ h ≤ 12, -15 ≤ k ≤ 15, -25 ≤ l ≤ 25, 23344 reflections measured, 8639 unique (R_{int} = 0.0983) R1 = 0.0877 (I>2.0σ(I)), wR2 = 0.3089 (all data), GooF = 1.078, 464 parameters, 0 restraints. Several sets of atoms have been refined with equal anisotropic displacement parameters. Rapid solvent loss even at low temperatures precluded a high resolution structure determination and an excellent structure solution.

1. M. van der Sluis, J. B. M. Wit and F. Bickelhaupt, *Organometallics*, 1996, **15**, 174-180.
2. C. Hay, M. Hissler, C. Fischmeister, J. Rault-Berthelot, L. Toupet, L. Nyulászi and R. Réau, *Chem. Eur. J.*, 2001, **7**, 4222-4236.
3. Bruker AXS Inc., Madison, Wisconsin, USA, 2007, p. SADABS.
4. Bruker AXS Inc., Madison, Wisconsin, USA, 2001, p. SAINT.
5. G. Sheldrick, *Acta Crystallogr., Sect. A: Found. Crystallogr.*, 2008, **64**, 112-122.
6. L. Farrugia, *J. Appl. Crystallogr.*, 1999, **32**, 837-838.
7. L. Farrugia, *J. Appl. Crystallogr.*, 1997, **30**, 565.
8. P. van der Sluis and A. L. Spek, *Acta Crystallogr., Sect. A: Found. Crystallogr.*, 1990, **46**, 194-201.

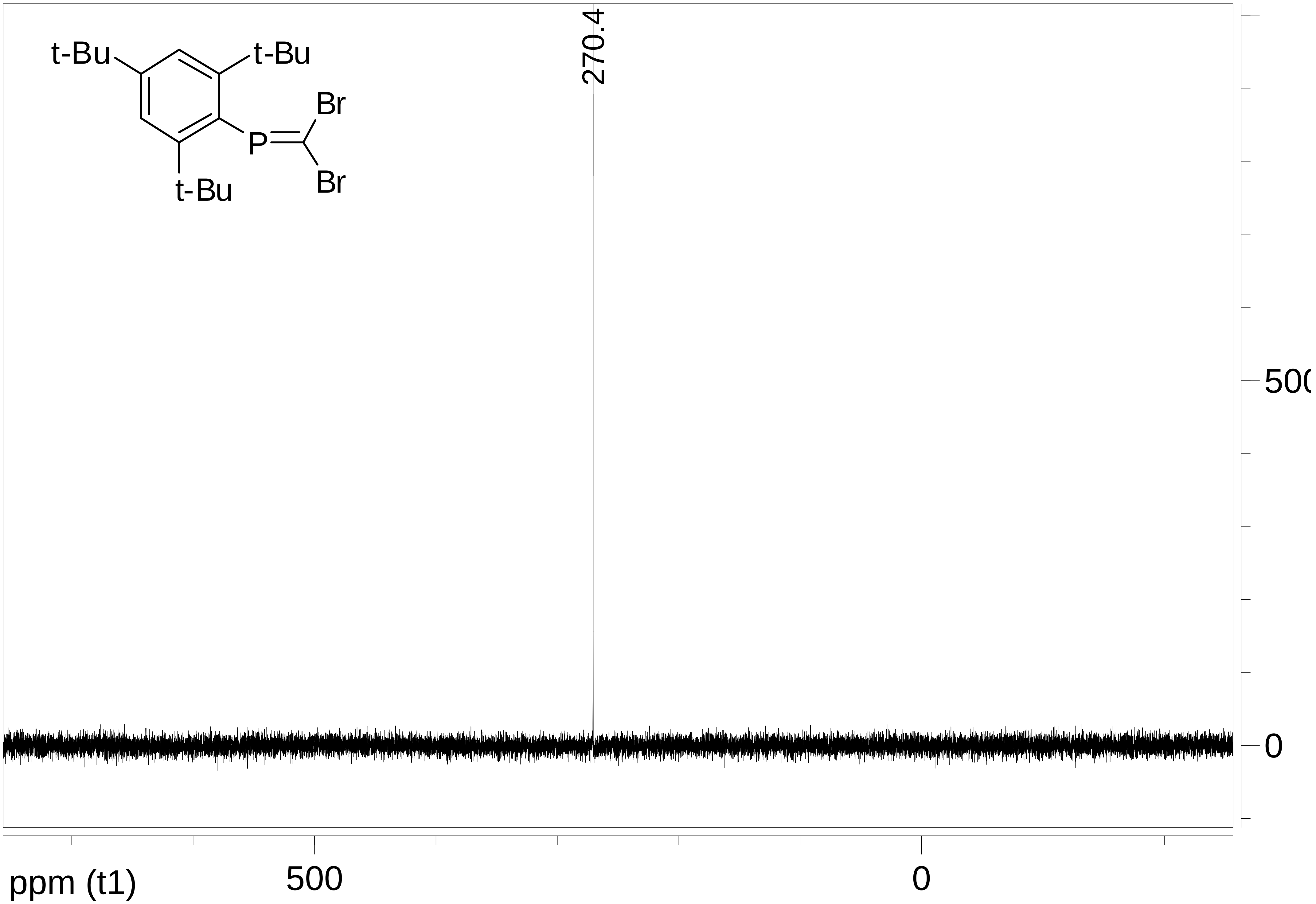
t-Bu
t-Bu
Br
P
Br
t-Bu
270.4
500
0
ppm (t1)
500
0

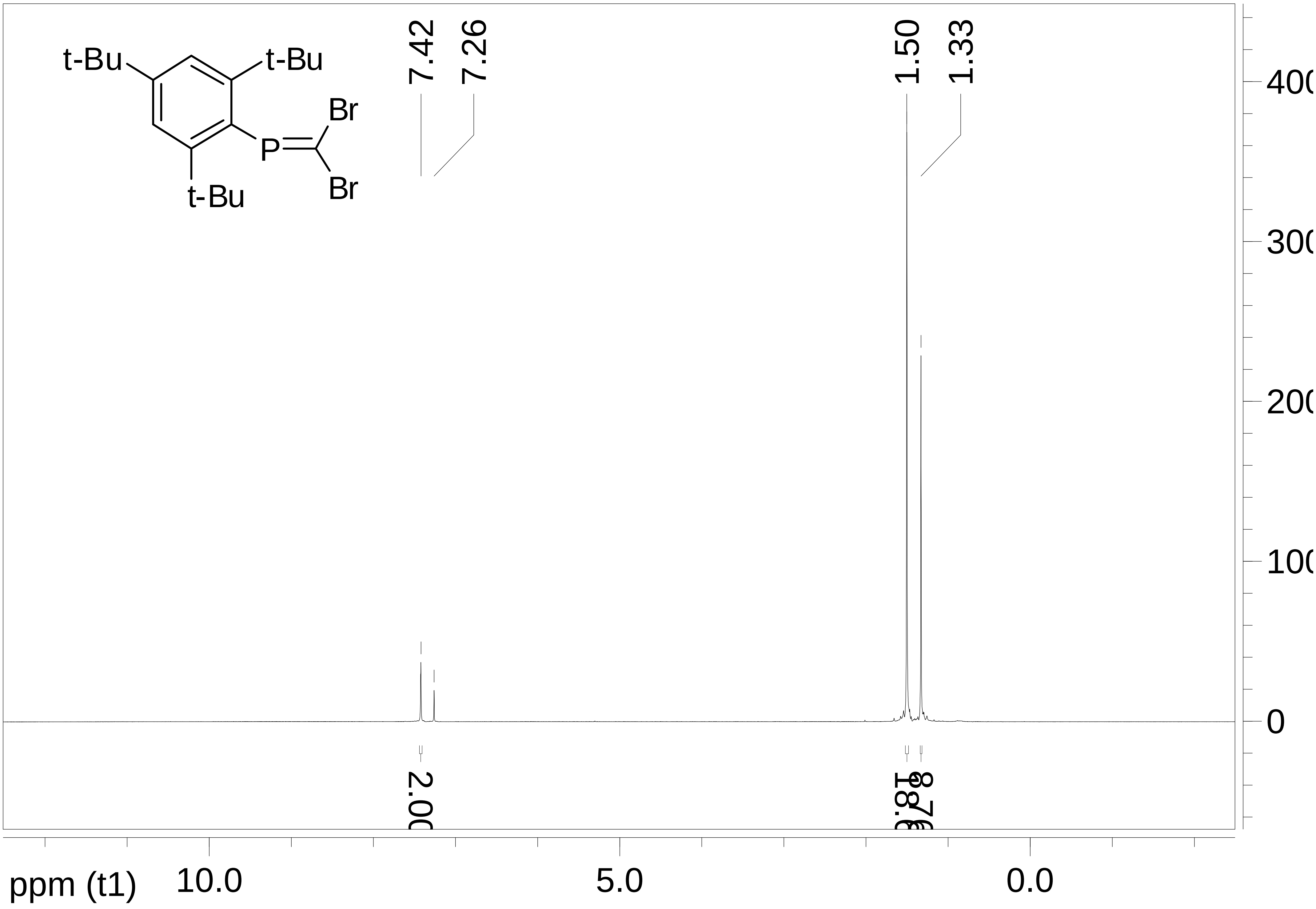

t-Bu
t-Bu
t-Bu
P
Br
Br
7.42
7.26
1.50
1.33
400
300
200
100
0
2.00
ppm (t1)
10.0
5.0
0.0

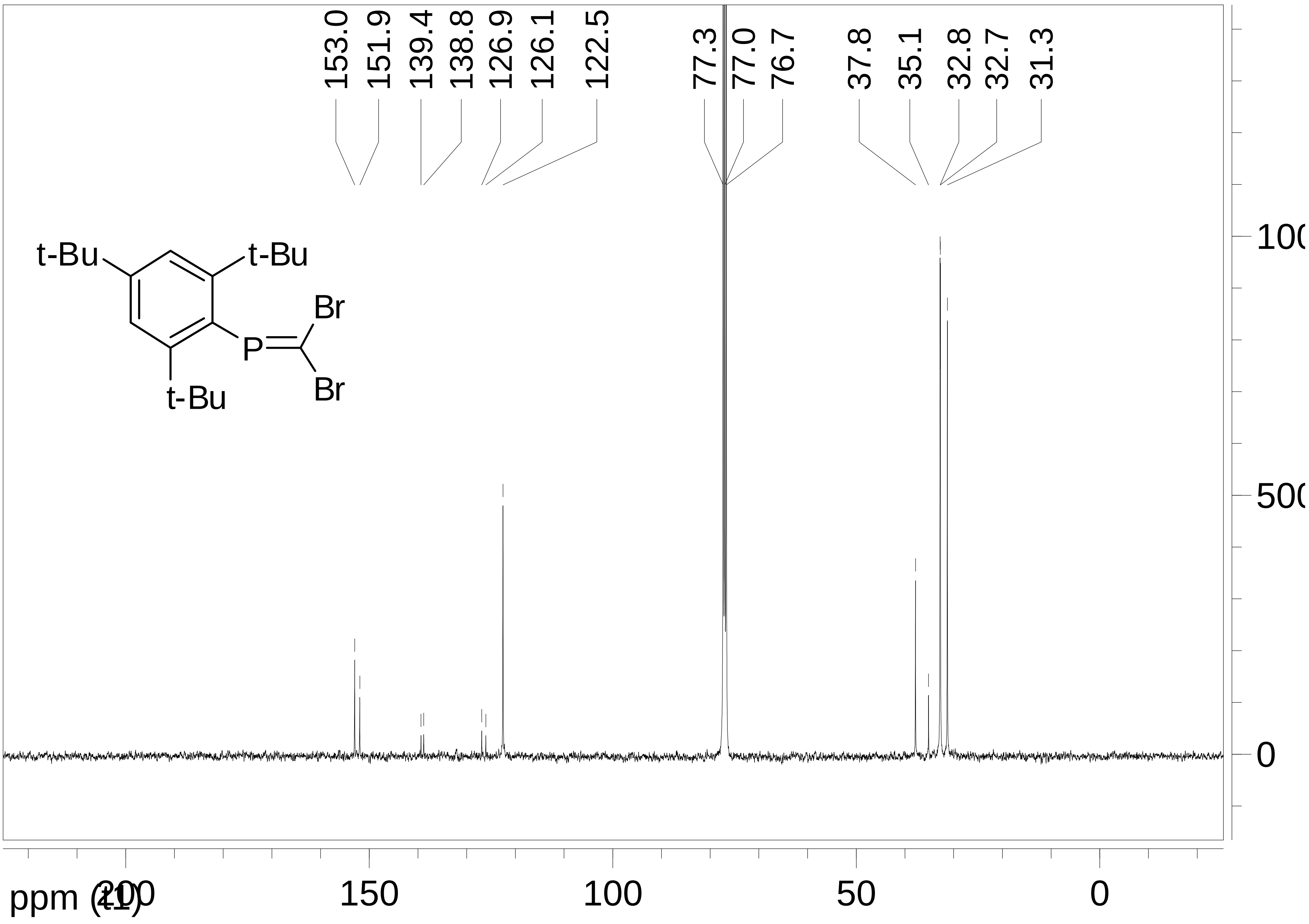

153.0
151.9
139.4
138.8
126.9
126.1
122.5
77.3
77.0
76.7
37.8
35.1
32.8
32.7
31.3
t-Bu
t-Bu
t-Bu
P
Br
Br
ppm (t1)
200
150
100
50
0

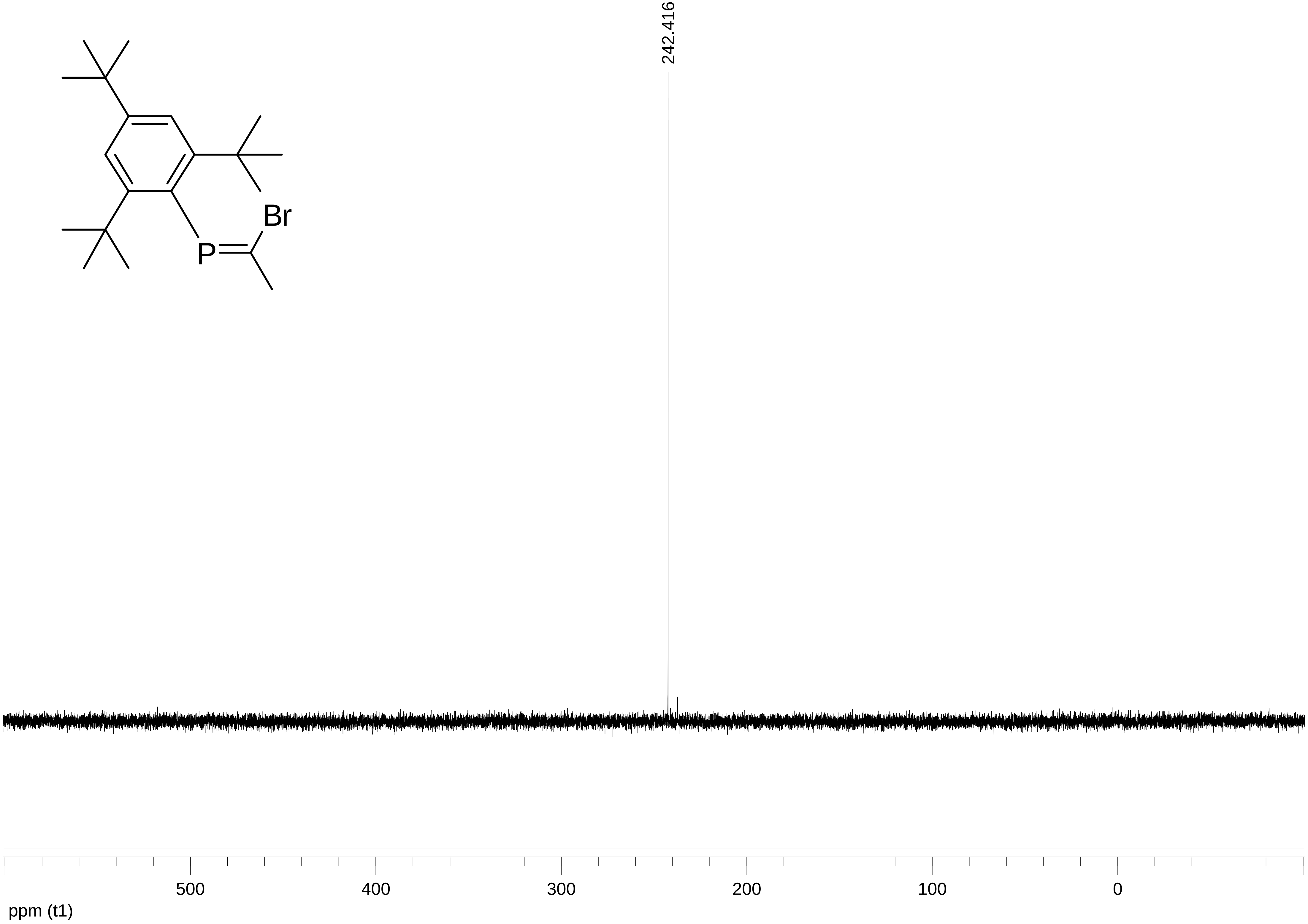
242.416
Br
P
500
400
300
200
100
0
ppm (t1)

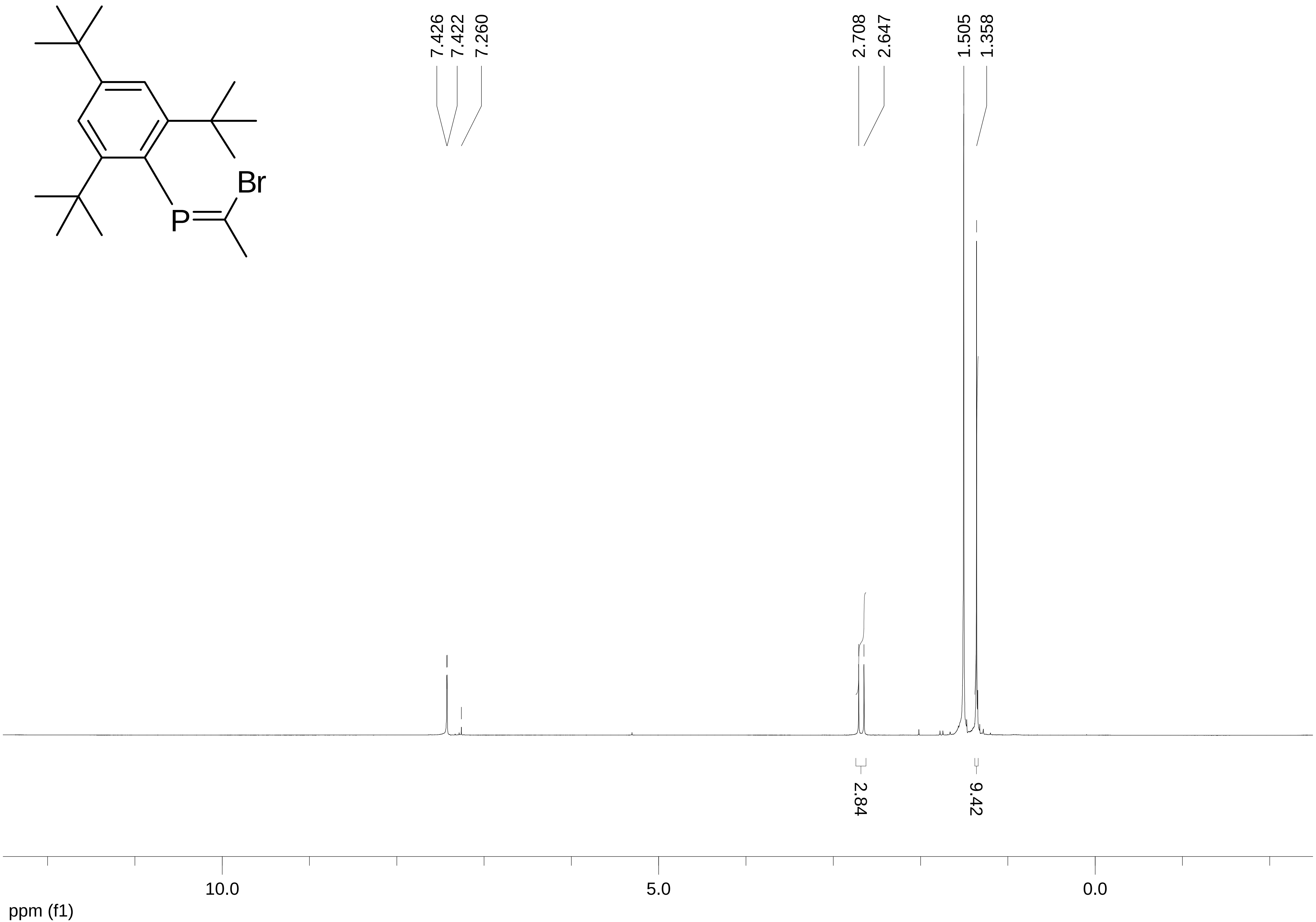
Br
P
7.426
7.422
7.260
2.708
2.647
1.505
1.358
2.84
9.42
10.0
5.0
0.0
ppm (f1)

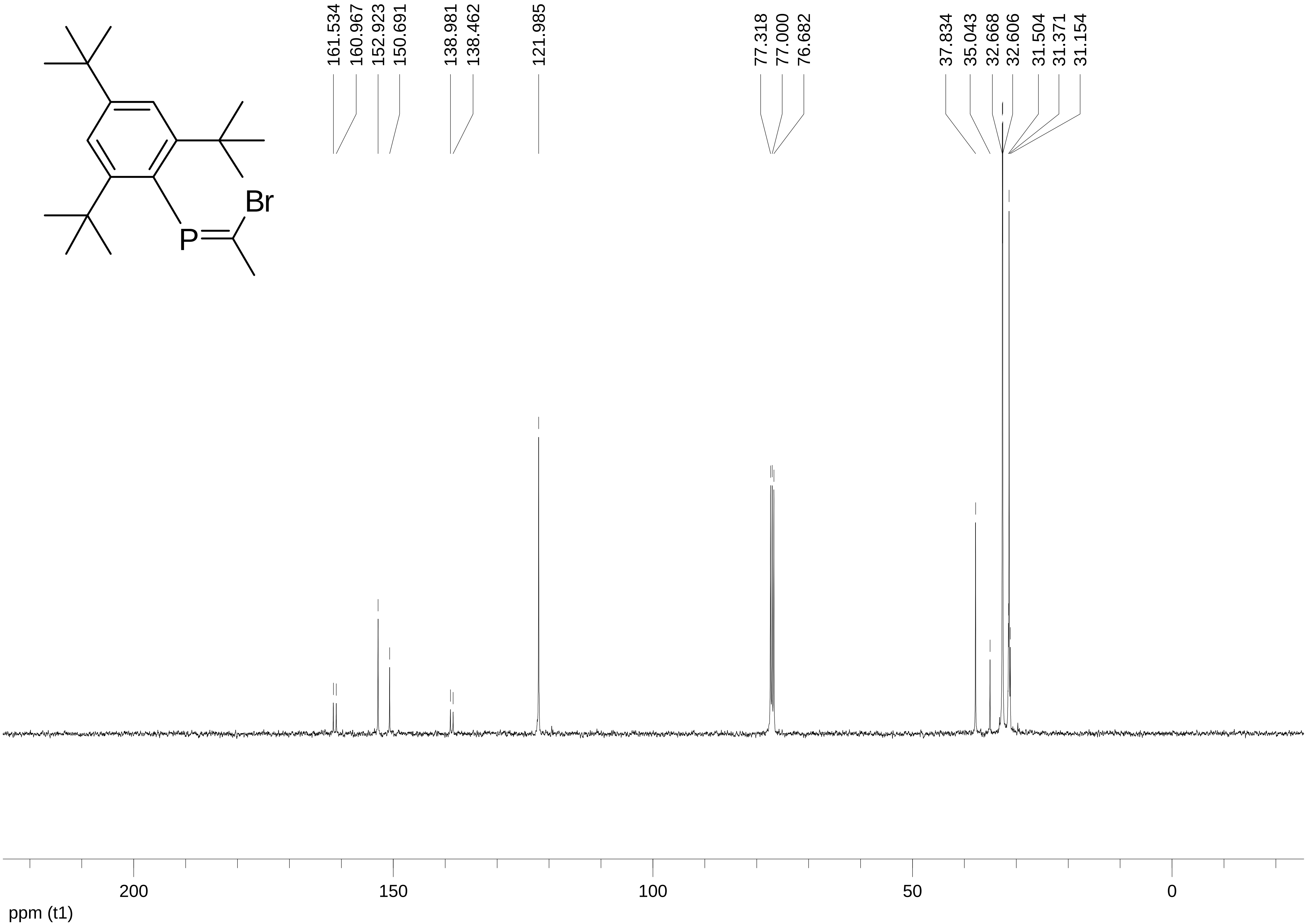
Br
P
161.534
160.967
152.923
150.691
138.981
138.462
121.985
77.318
77.000
76.682
37.834
35.043
32.668
32.606
31.504
31.371
31.154
200
150
100
50
0
ppm (t1)

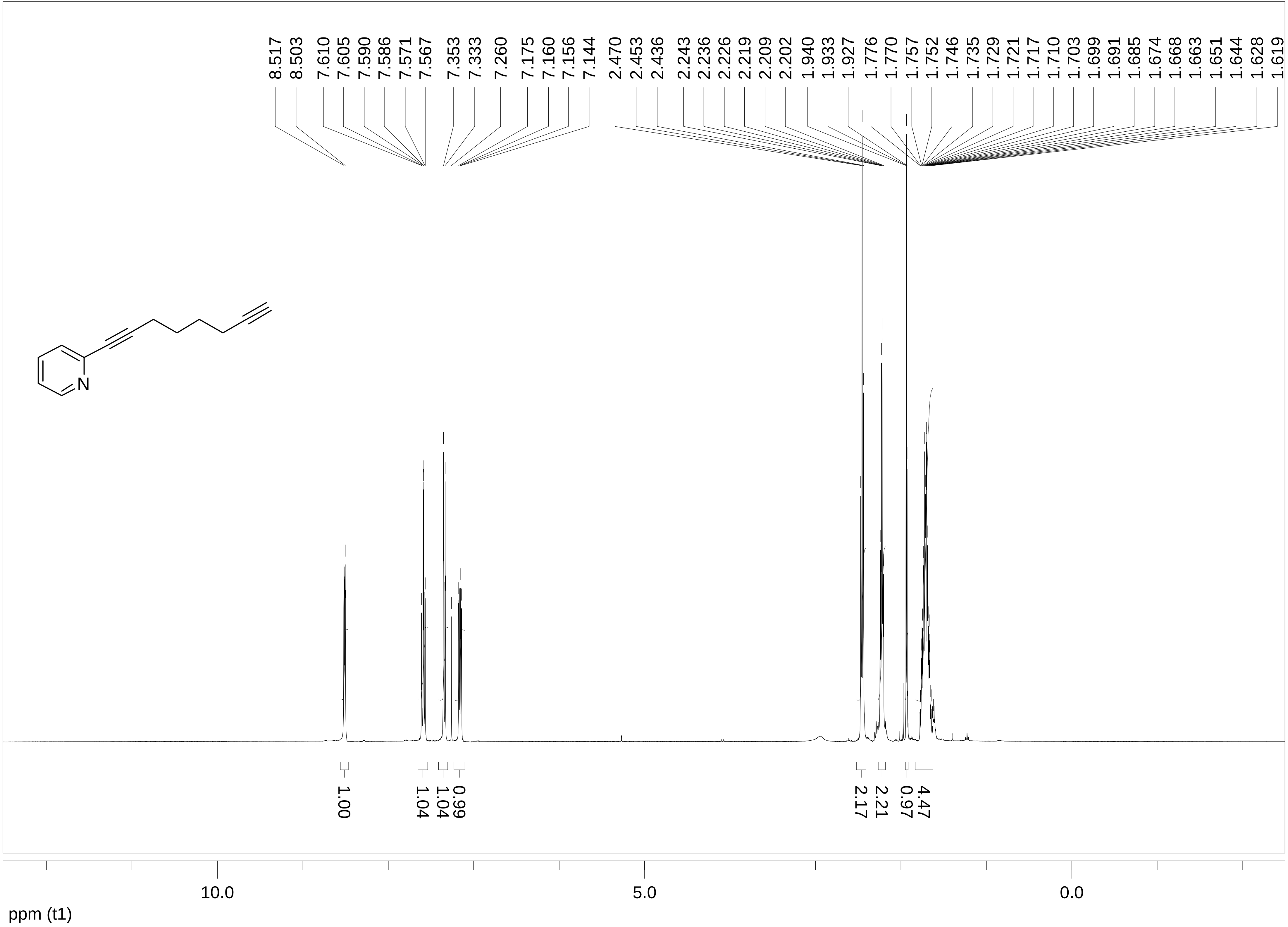

8.517
8.503
7.610
7.605
7.590
7.586
7.571
7.567
7.353
7.333
7.260
7.175
7.160
7.156
7.144
2.470
2.453
2.436
2.243
2.236
2.226
2.219
2.209
2.202
1.940
1.933
1.927
1.776
1.770
1.757
1.752
1.746
1.735
1.729
1.721
1.717
1.710
1.703
1.699
1.691
1.685
1.674
1.668
1.663
1.651
1.644
1.628
1.619
N
1.00
1.04
1.04
0.99
2.17
2.21
0.97
4.47
10.0
5.0
0.0
ppm (t1)

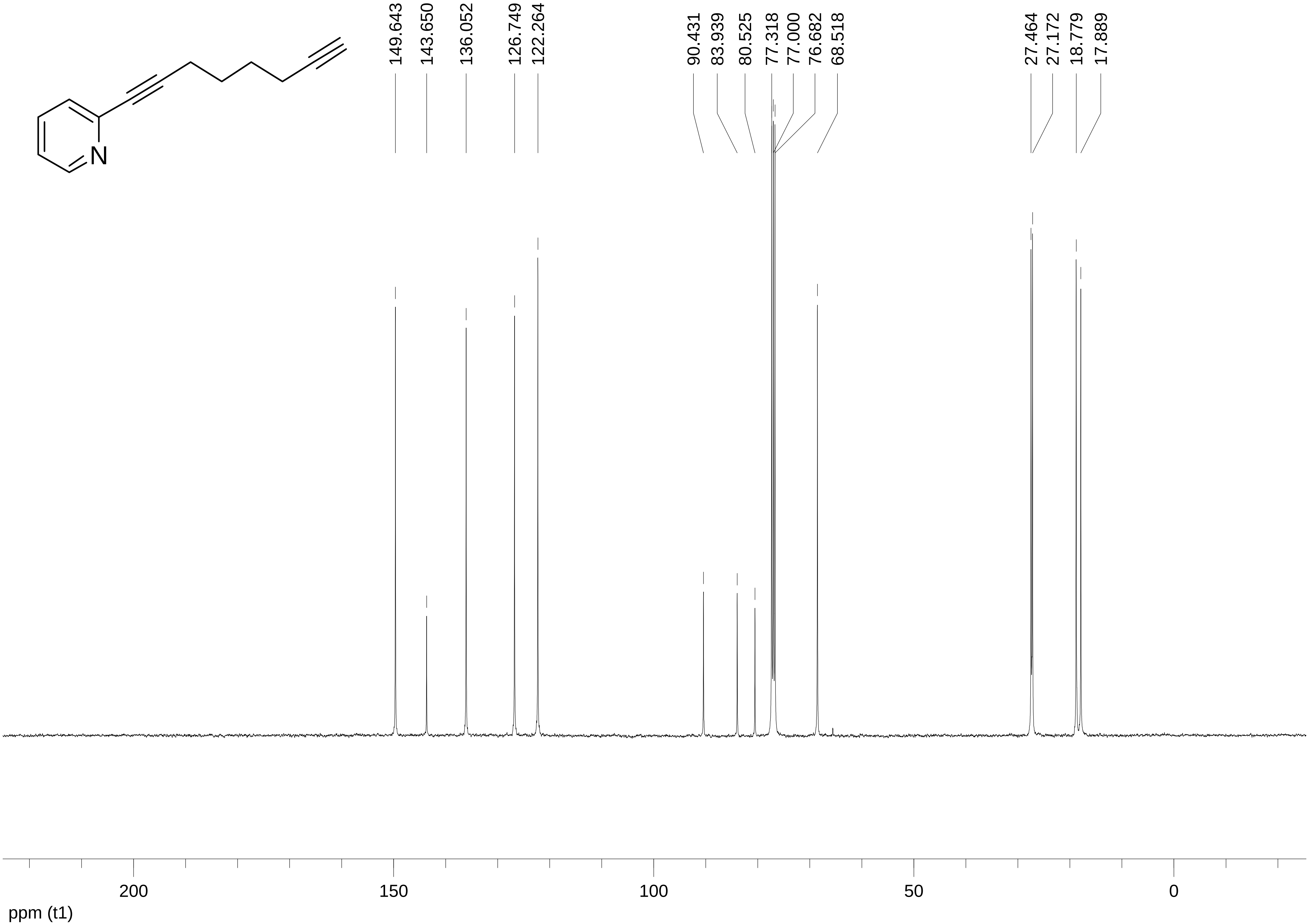
N
149.643
143.650
136.052
126.749
122.264
90.431
83.939
80.525
77.318
77.000
76.682
68.518
27.464
27.172
18.779
17.889
200
150
100
50
0
ppm (t1)

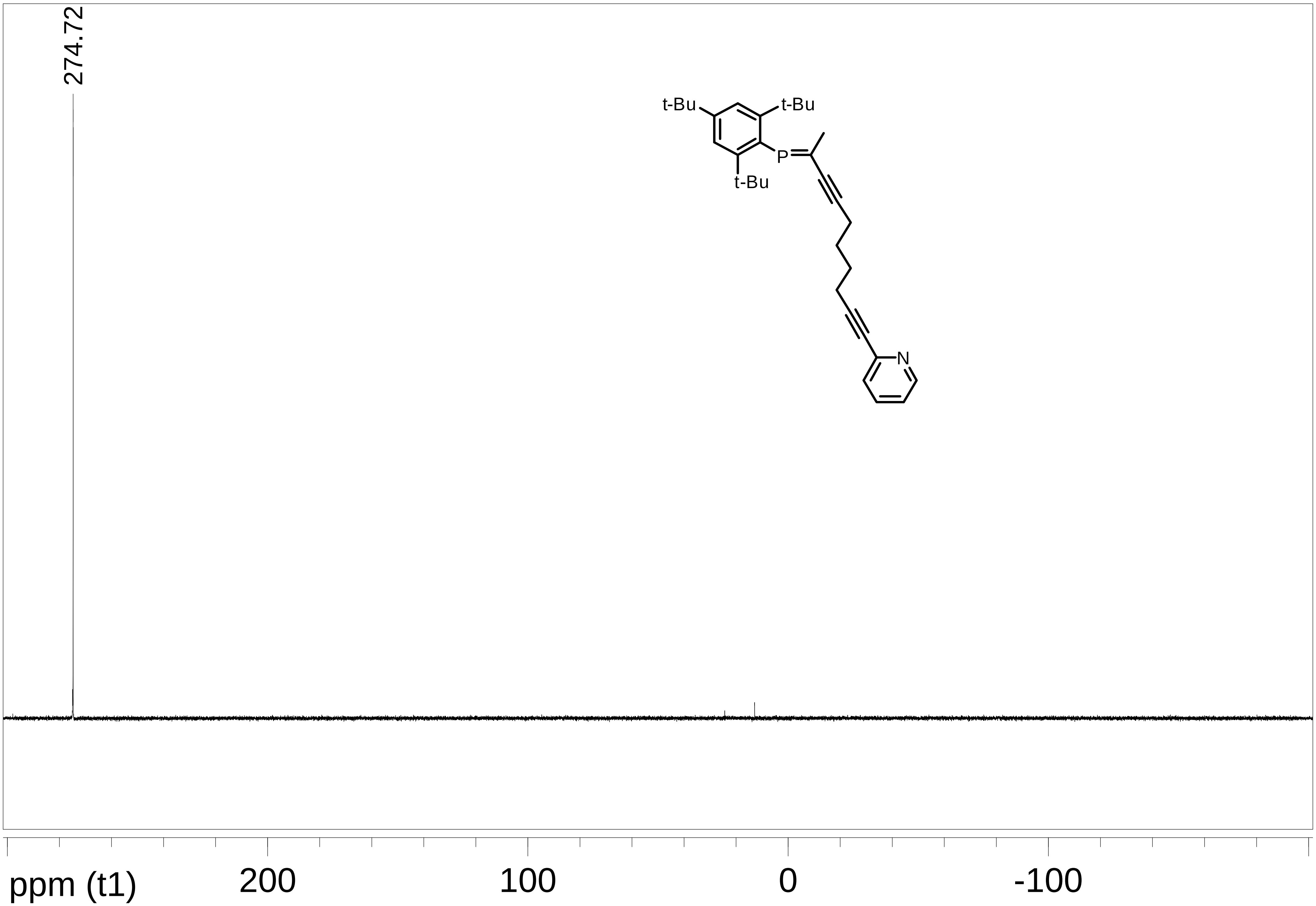
274.72
t-Bu
t-Bu
t-Bu
P
N
ppm (t1)
200
100
0
-100

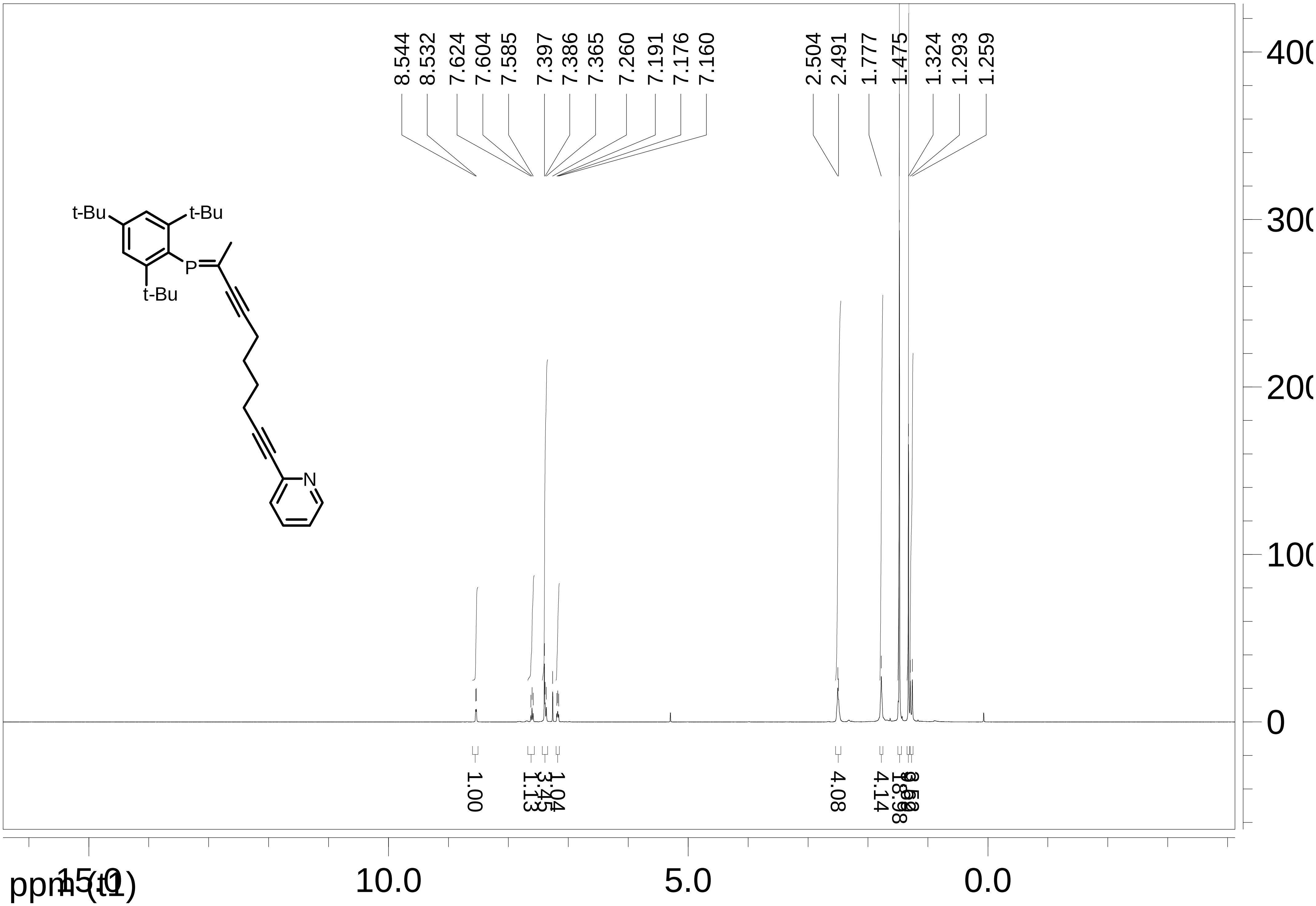
8.544
8.532
7.624
7.604
7.585
7.397
7.386
7.365
7.260
7.191
7.176
7.160
2.504
2.491
1.777
1.475
1.324
1.293
1.259
t-Bu
t-Bu
t-Bu
P
N
1.00
1.13
3.45
1.04
4.08
4.14
0
10.0
5.0
0.0
ppm (t1)

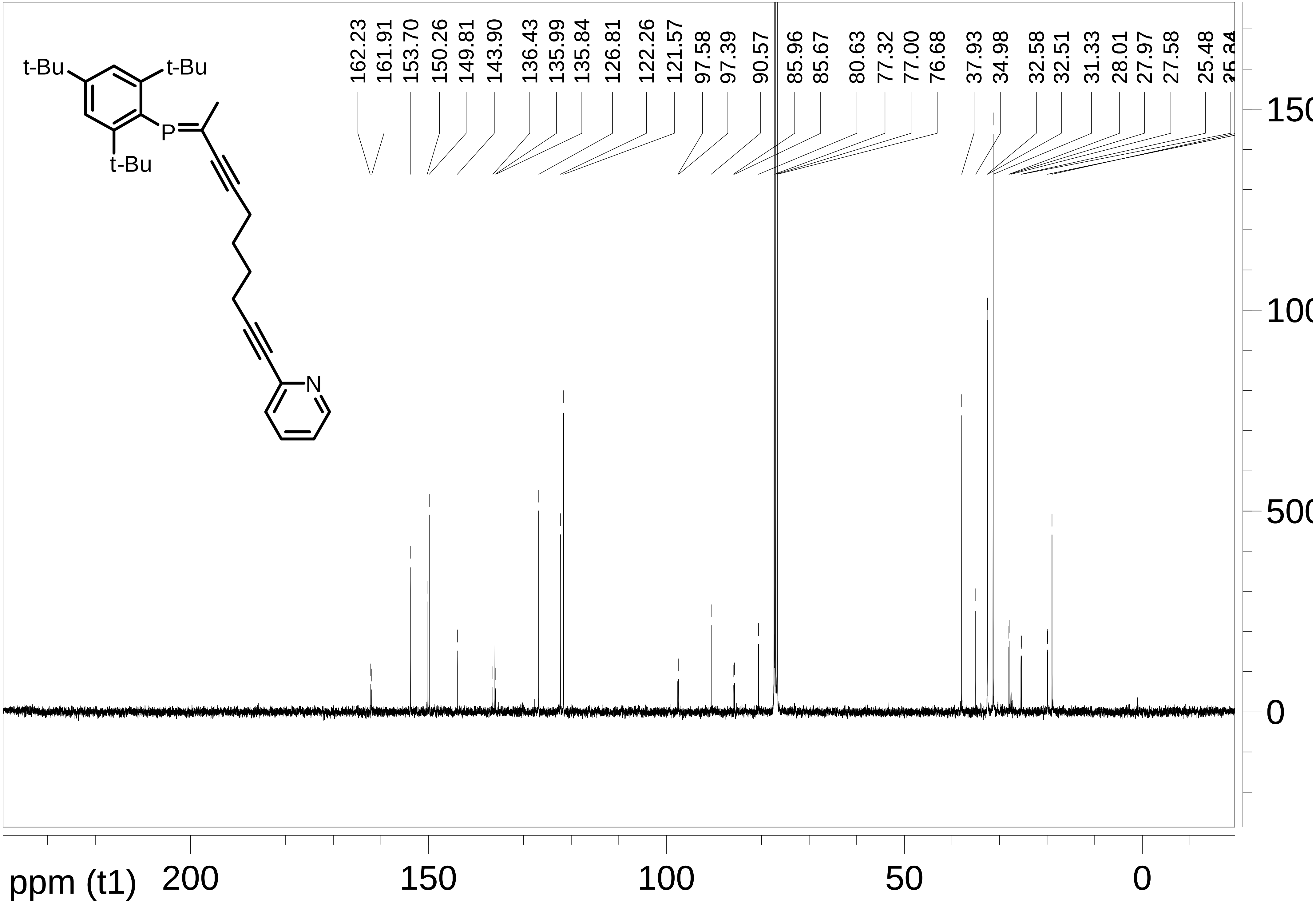
162.23
161.91
153.70
150.26
149.81
143.90
136.43
135.99
135.84
126.81
122.26
121.57
97.58
97.39
90.57
85.96
85.67
80.63
77.32
77.00
76.68
37.93
34.98
32.58
32.51
31.33
28.01
27.97
27.58
25.48
t-Bu
t-Bu
t-Bu
P
N
ppm (t1)
200
150
100
50
0

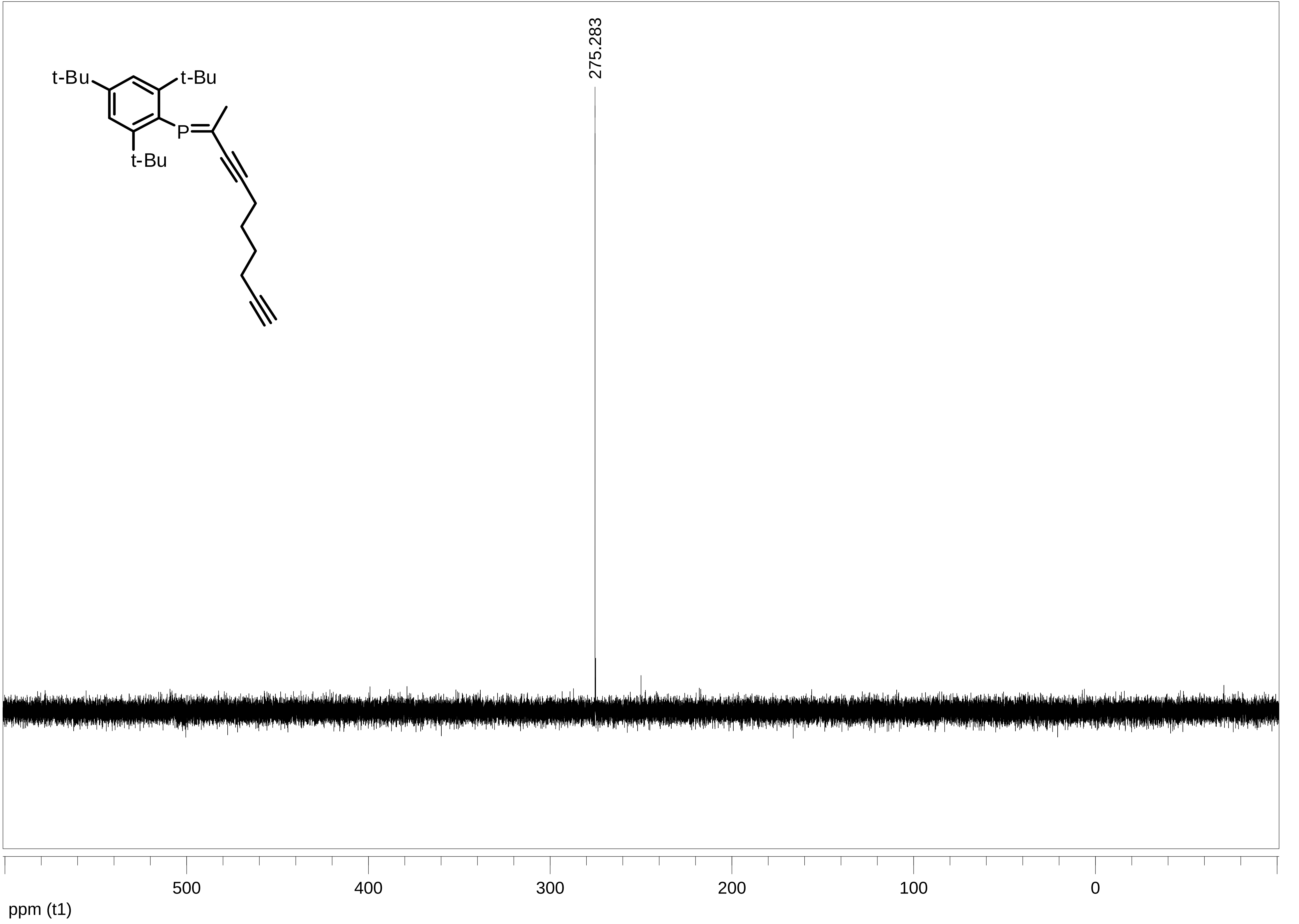

t-Bu
t-Bu
P
t-Bu
275.283
500
400
300
200
100
0
ppm (t1)

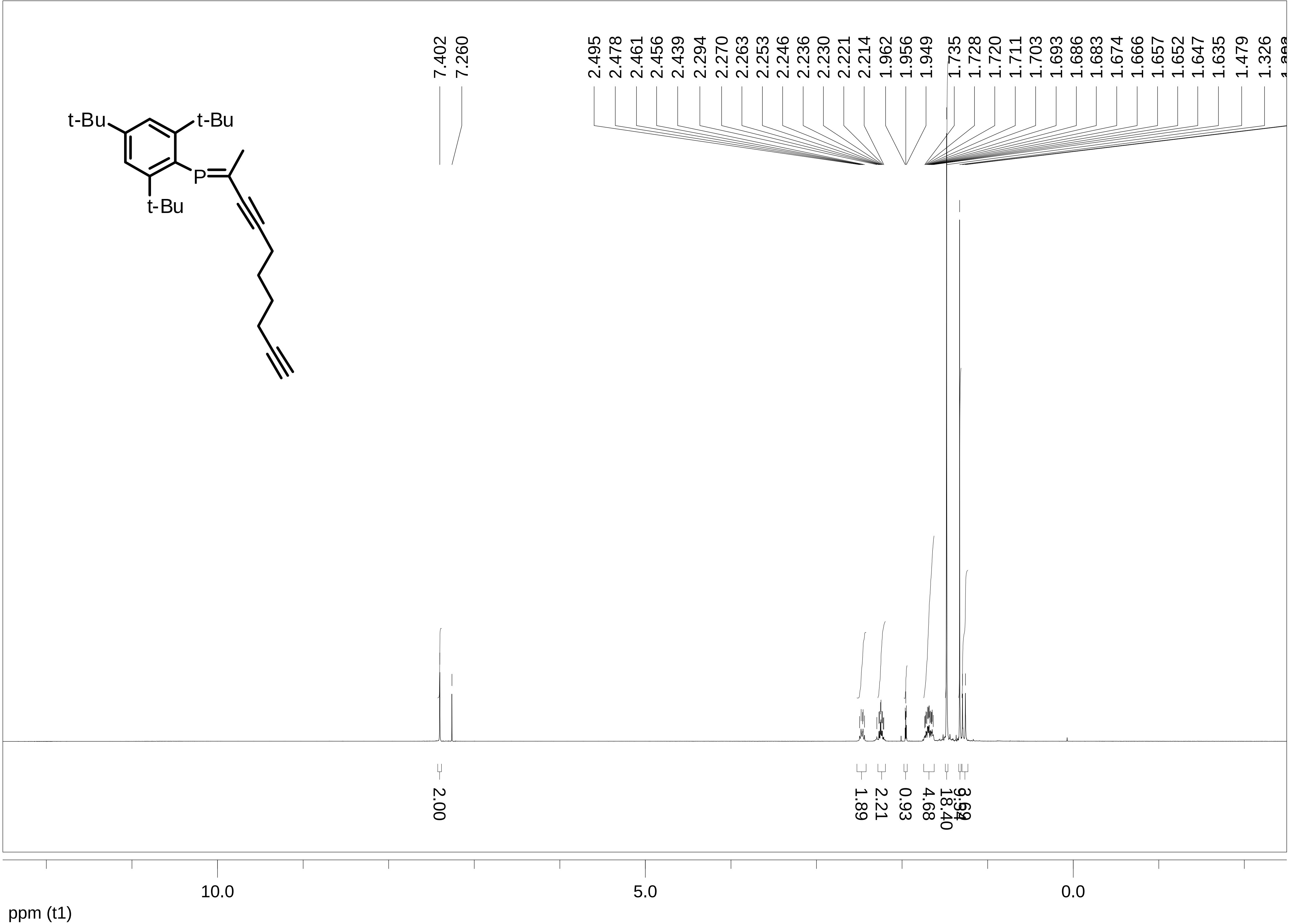
t-Bu
t-Bu
t-Bu
P
7.402
7.260
2.495
2.478
2.461
2.456
2.439
2.294
2.270
2.263
2.253
2.246
2.236
2.230
2.221
2.214
1.962
1.956
1.949
1.735
1.728
1.720
1.711
1.703
1.693
1.686
1.683
1.674
1.666
1.657
1.652
1.647
1.635
1.479
1.326
2.00
1.89
2.21
0.93
4.68
18.40
10.0
5.0
0.0
ppm (t1)

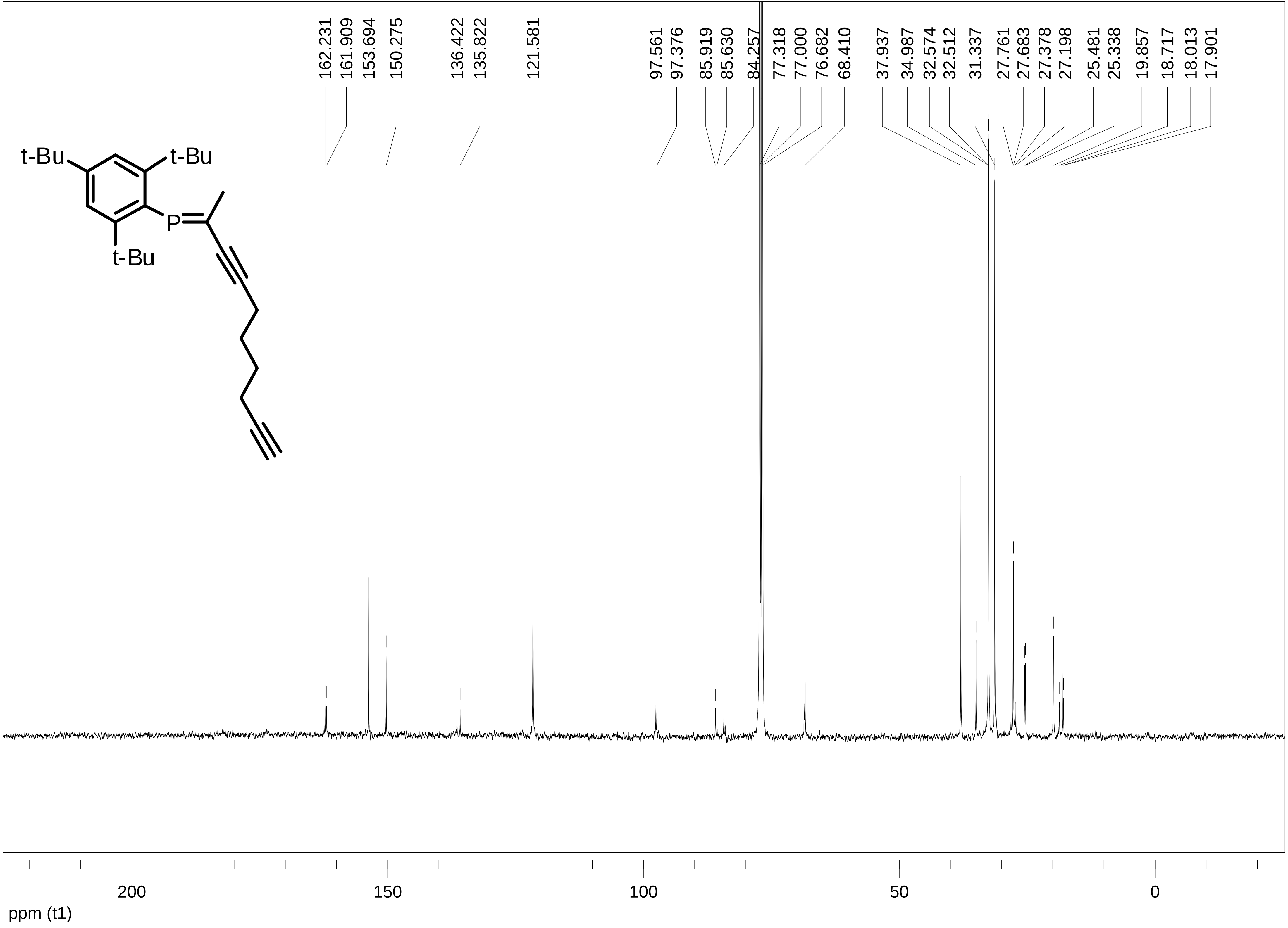
162.231
161.909
153.694
150.275
136.422
135.822
121.581
97.561
97.376
85.919
85.630
84.257
77.318
77.000
76.682
68.410
37.937
34.987
32.574
32.512
31.337
27.761
27.683
27.378
27.198
25.481
25.338
19.857
18.717
18.013
17.901
t-Bu
t-Bu
P
t-Bu
200
150
100
50
0
ppm (t1)

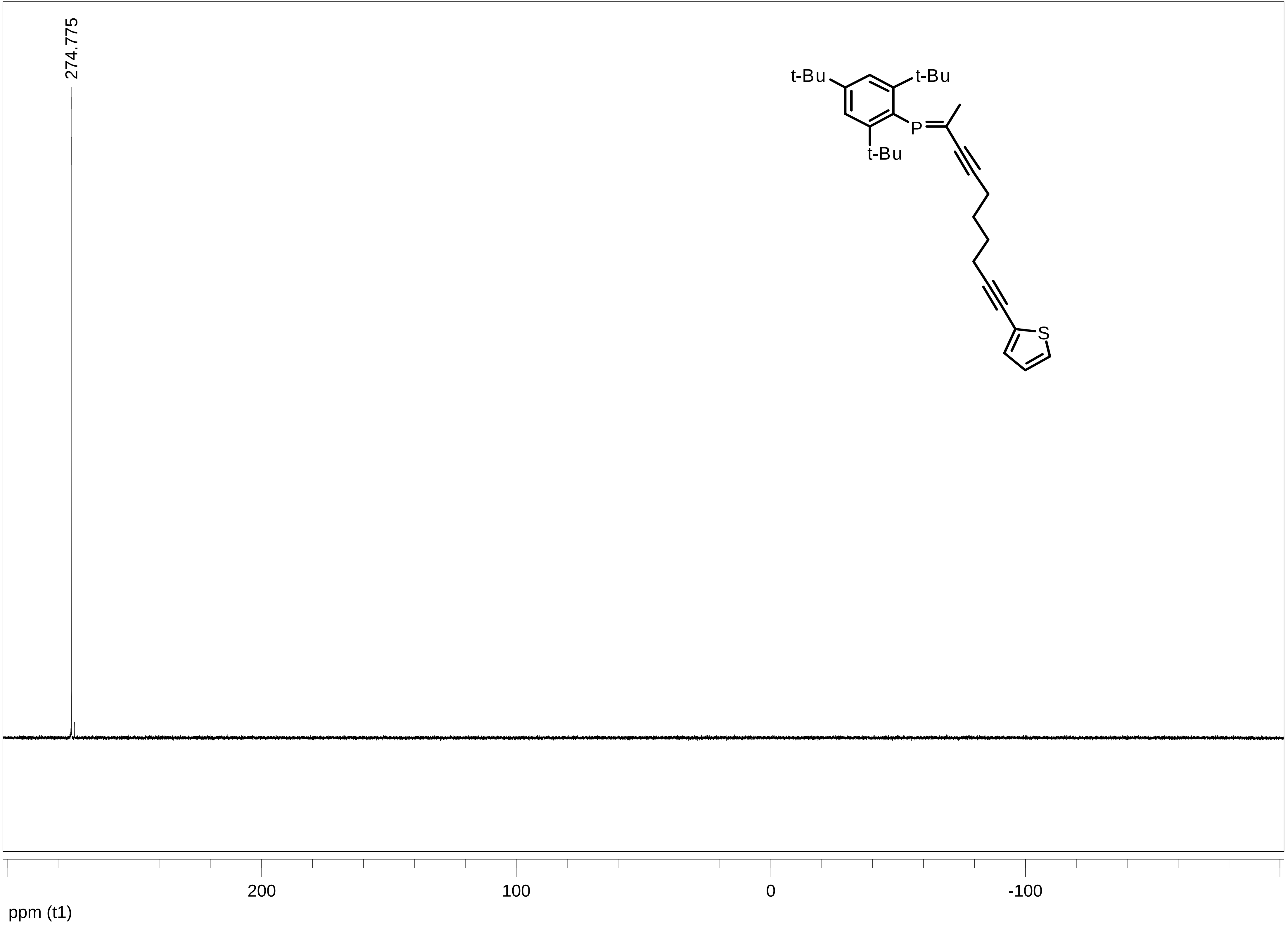
274.775
t-Bu
t-Bu
P
t-Bu
S
200
100
0
-100
ppm (t1)

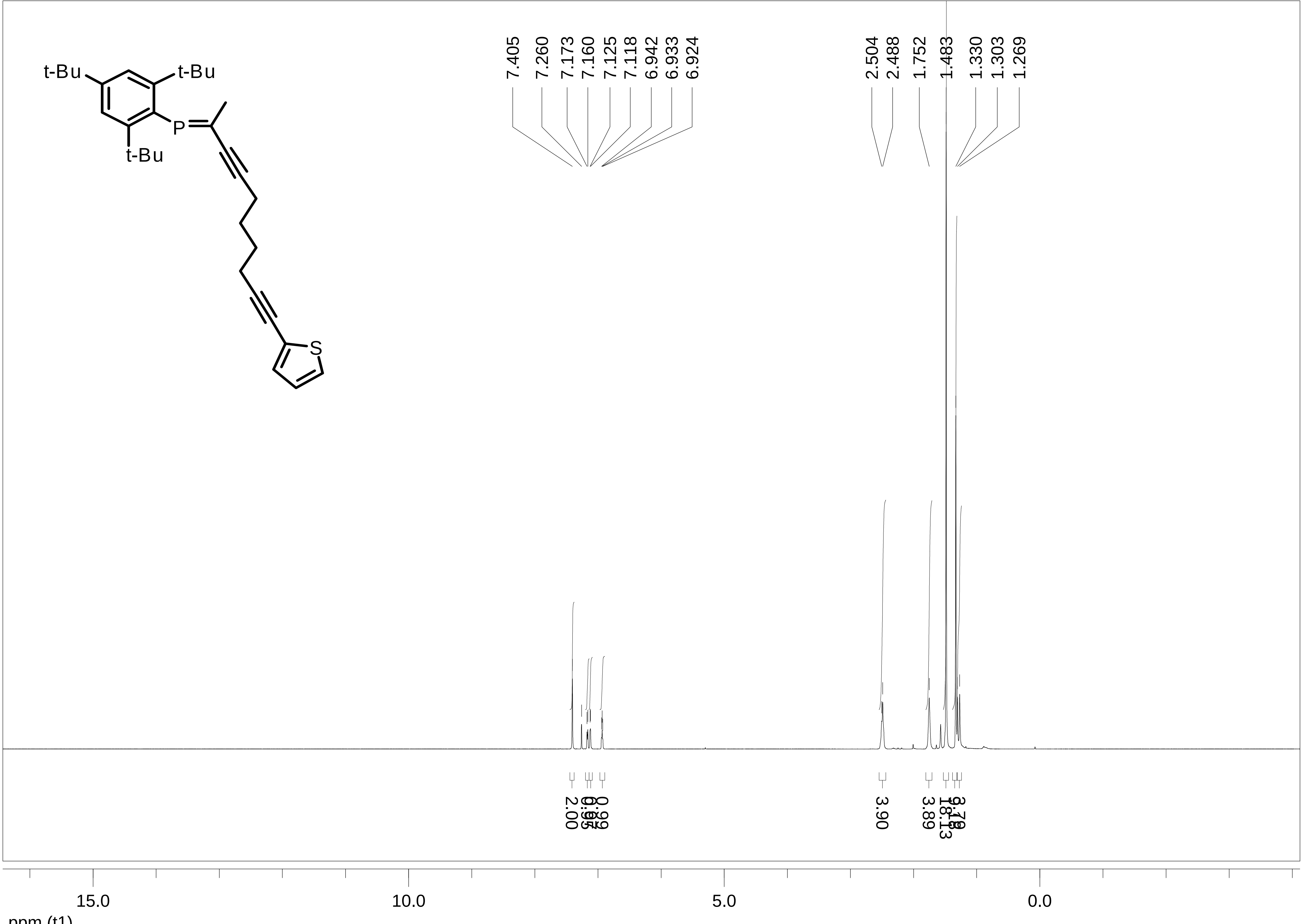

t-Bu
t-Bu
P
t-Bu
S
7.405
7.260
7.173
7.160
7.125
7.118
6.942
6.933
6.924
2.504
2.488
1.752
1.483
1.330
1.303
1.269
2.00
3.90
3.89
15.0
10.0
5.0
0.0
ppm (t1)

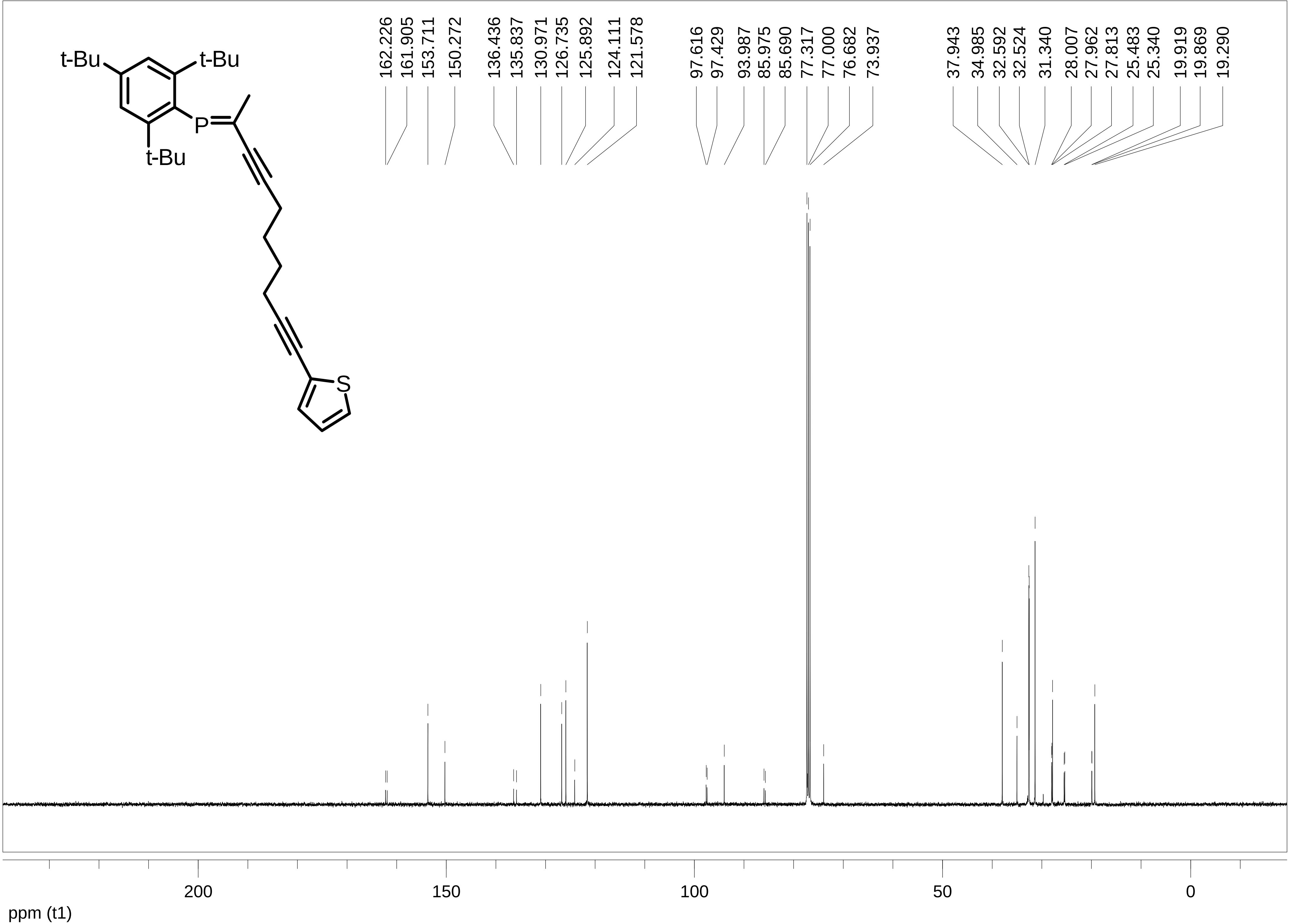
t-Bu
t-Bu
P
t-Bu
S
162.226
161.905
153.711
150.272
136.436
135.837
130.971
126.735
125.892
124.111
121.578
97.616
97.429
93.987
85.975
85.690
77.317
77.000
76.682
73.937
37.943
34.985
32.592
32.524
31.340
28.007
27.962
27.813
25.483
25.340
19.919
19.869
19.290
200
150
100
50
0
ppm (t1)

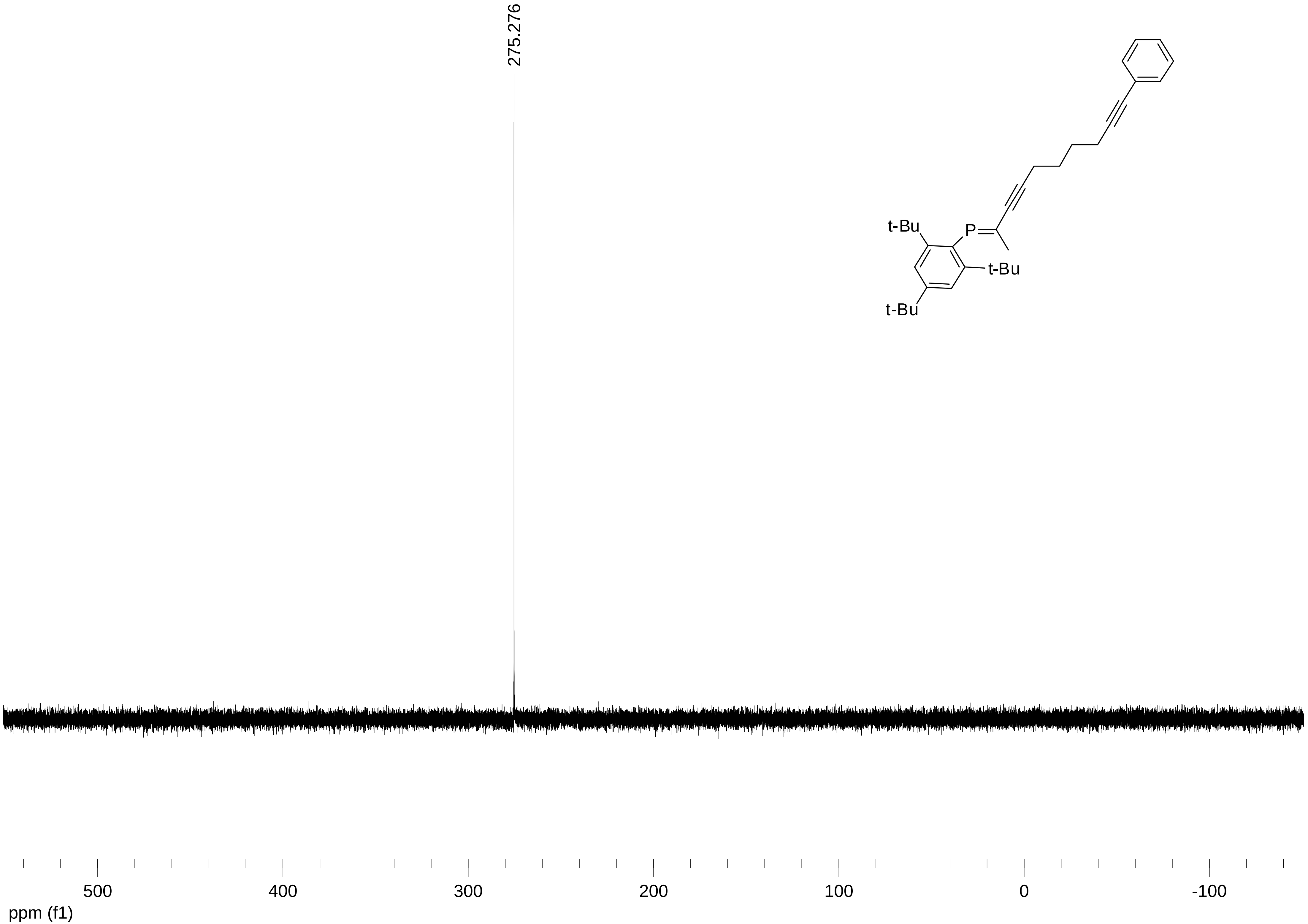
275.276
t-Bu
P
t-Bu
t-Bu
500
400
300
200
100
0
-100
ppm (f1)

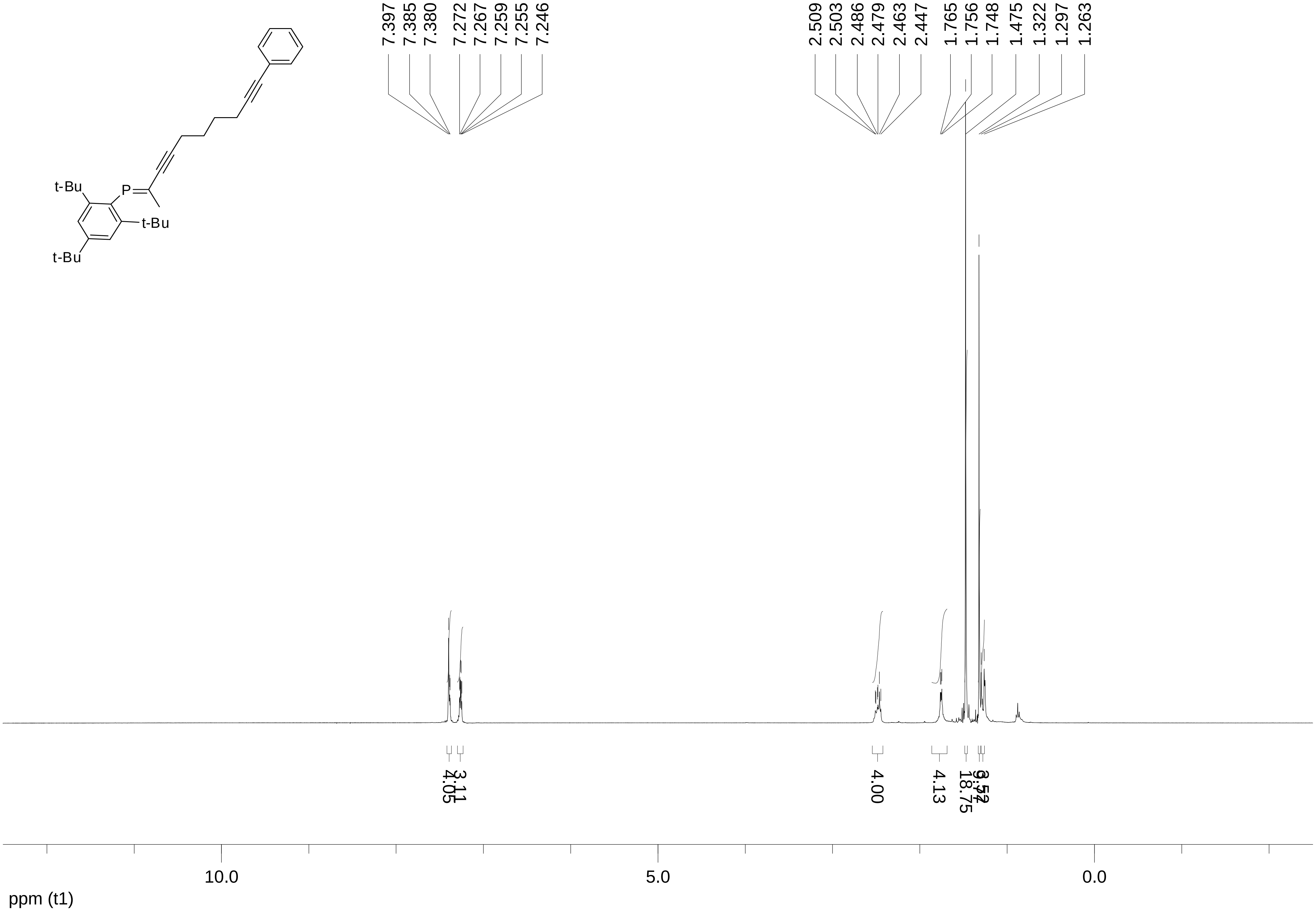

t-Bu
P
t-Bu
t-Bu
7.397
7.385
7.380
7.272
7.267
7.259
7.255
7.246
2.509
2.503
2.486
2.479
2.463
2.447
1.765
1.756
1.748
1.475
1.322
1.297
1.263
4.05
3.11
4.00
4.13
18.75
10.0
5.0
0.0
ppm (t1)

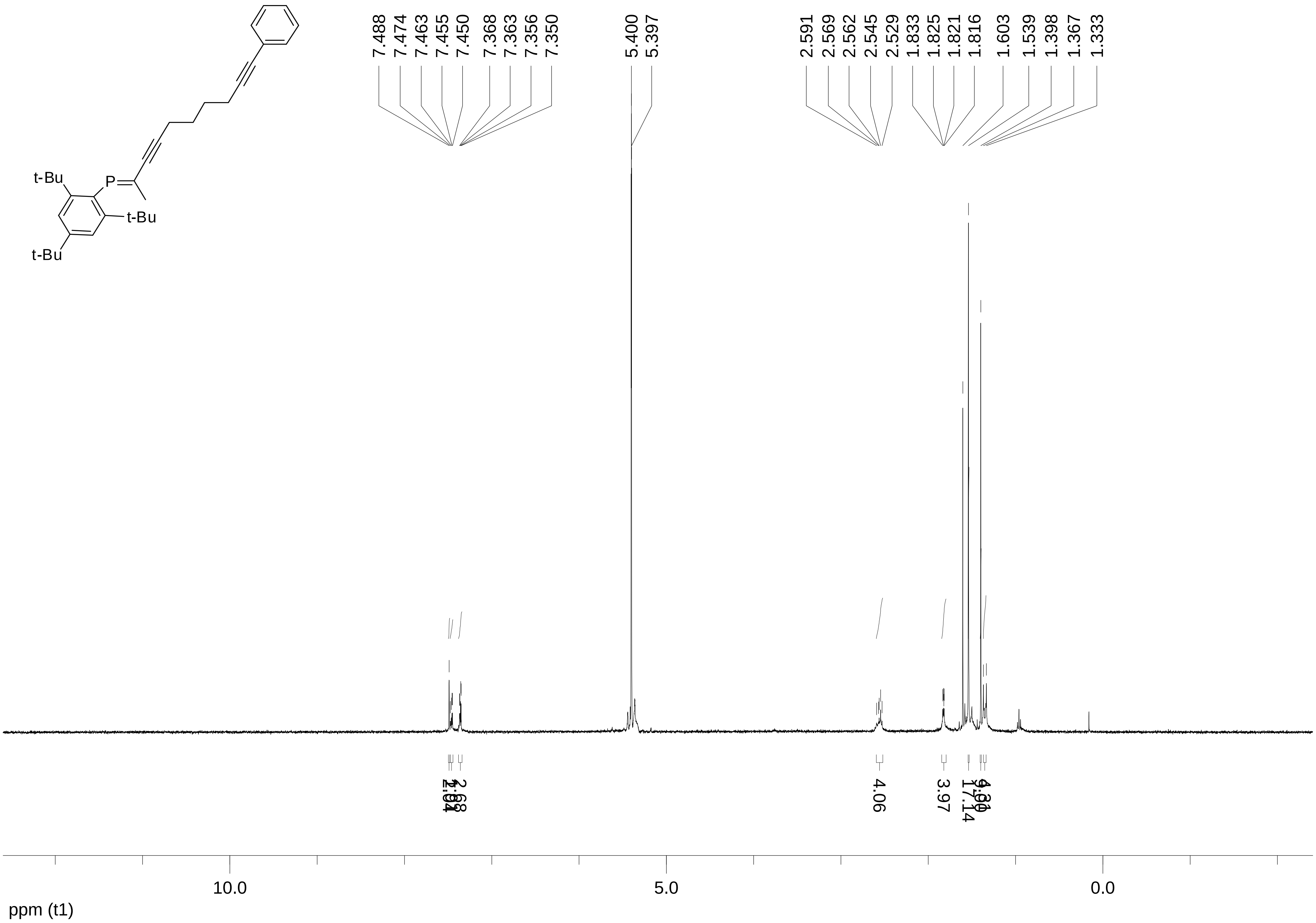

t-Bu
P
t-Bu
t-Bu
7.488
7.474
7.463
7.455
7.450
7.368
7.363
7.356
7.350
5.400
5.397
2.591
2.569
2.562
2.545
2.529
1.833
1.825
1.821
1.816
1.603
1.539
1.398
1.367
1.333
2.68
4.06
3.97
17.14
10.0
5.0
0.0
ppm (t1)

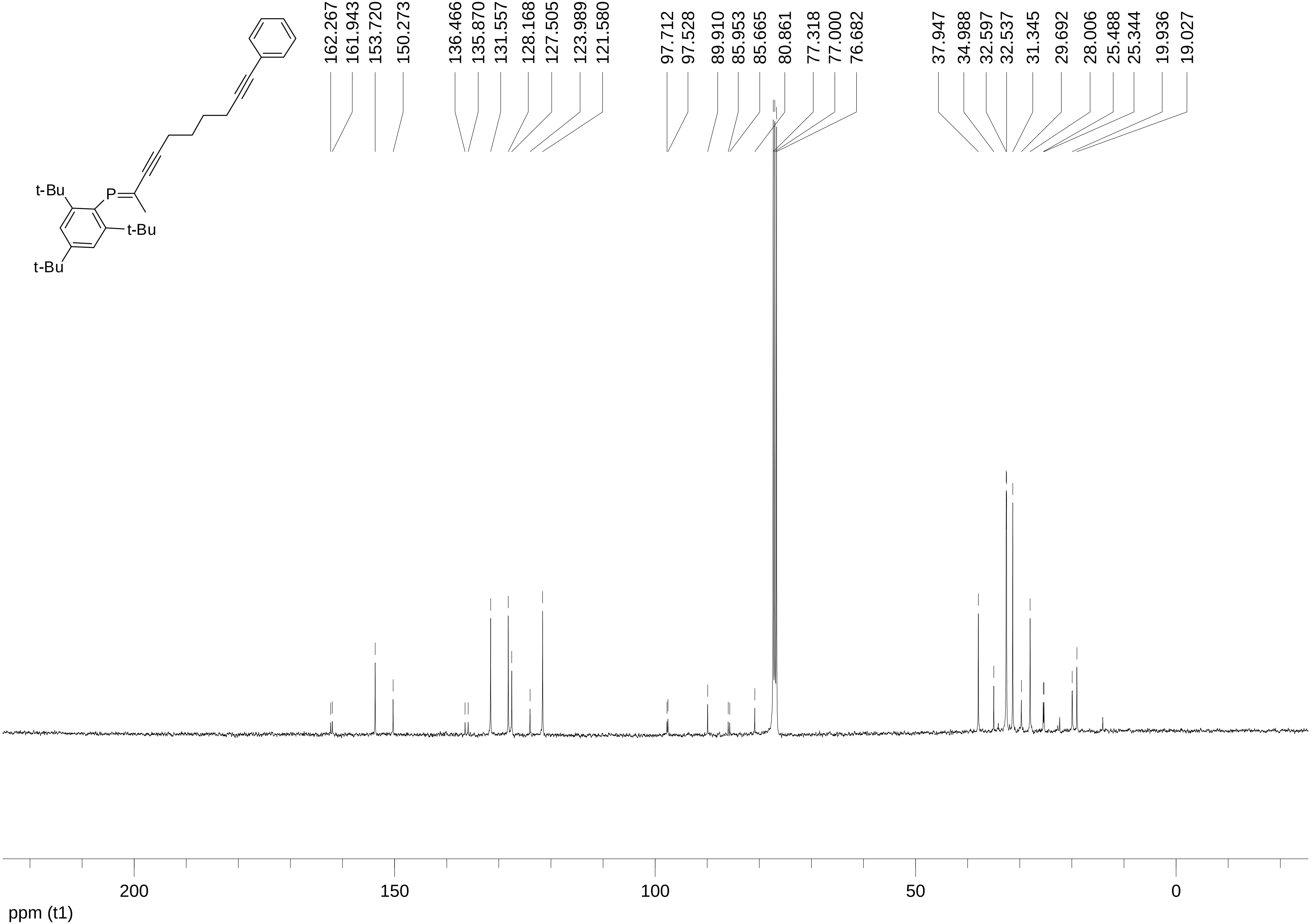
t-Bu
P
t-Bu
t-Bu
162.267
161.943
153.720
150.273
136.466
135.870
131.557
128.168
127.505
123.989
121.580
97.712
97.528
89.910
85.953
85.665
80.861
77.318
77.000
76.682
37.947
34.988
32.597
32.537
31.345
29.692
28.006
25.488
25.344
19.936
19.027
200
150
100
50
0
ppm (t1)

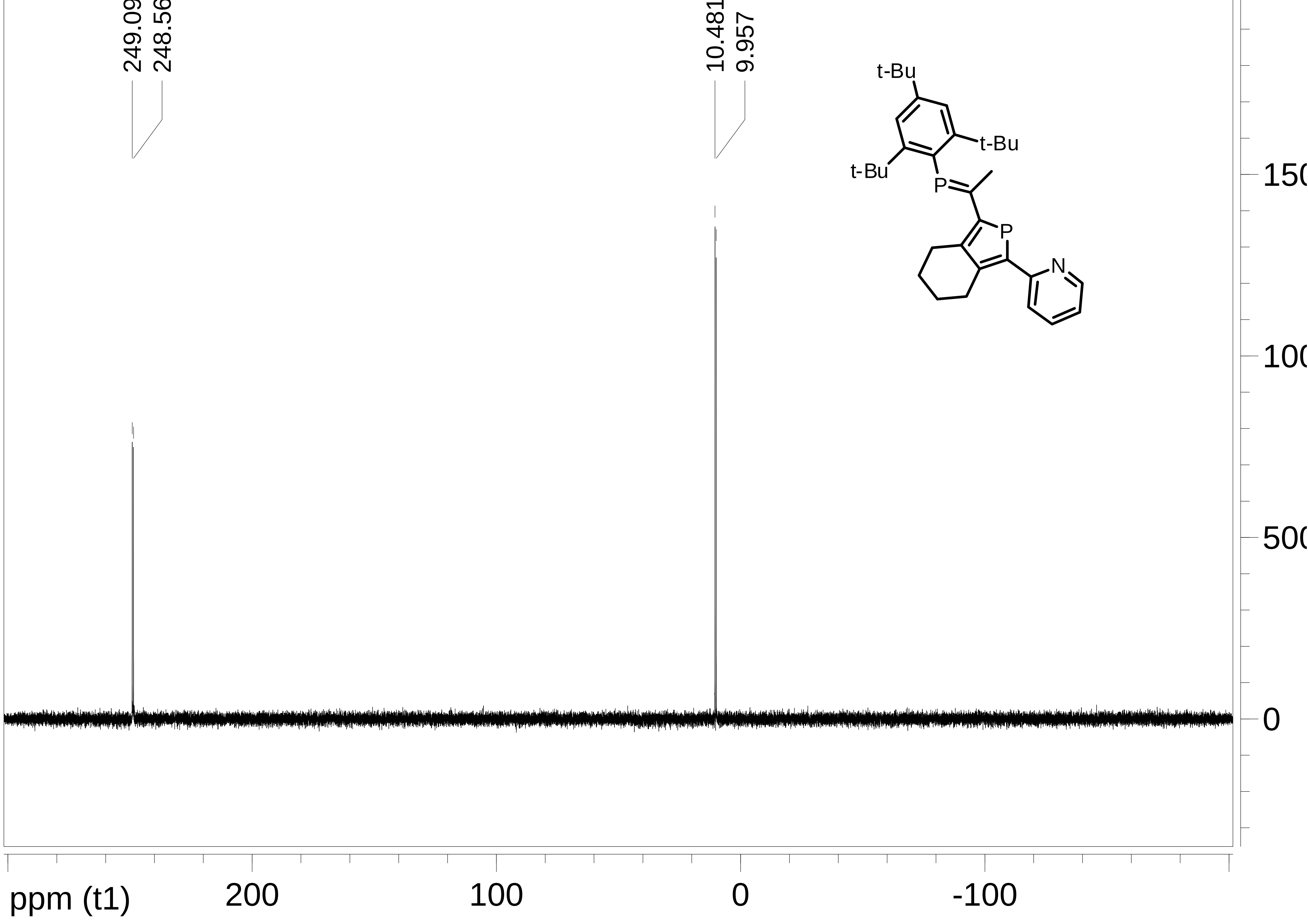

249.09
248.56
10.481
9.957
t-Bu
t-Bu
t-Bu
P
P
N
150
100
500
0
ppm (t1)
200
100
0
-100

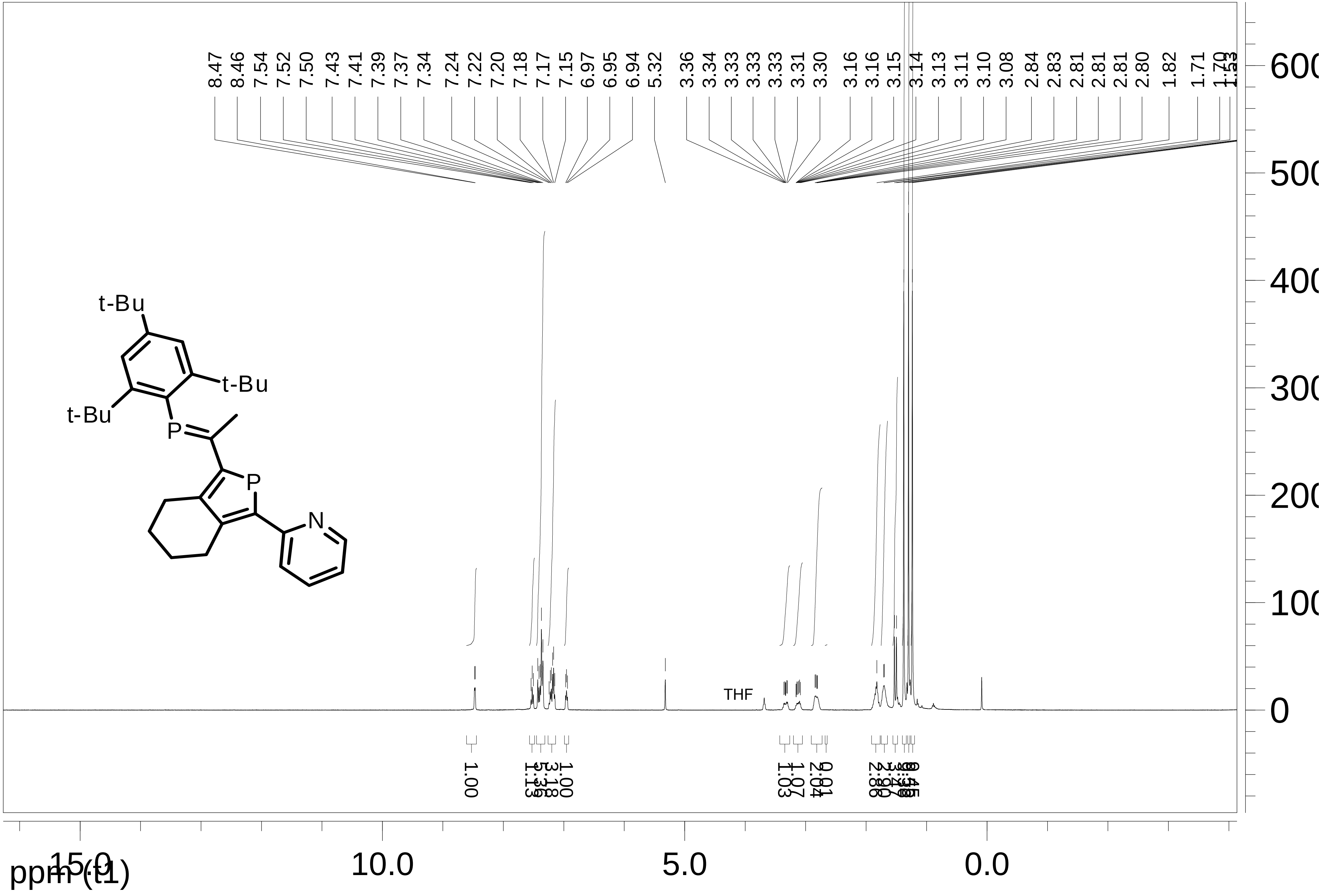

t-Bu
t-Bu
t-Bu
P
P
N
THF
ppm (t1)
15.0
10.0
5.0
0.0

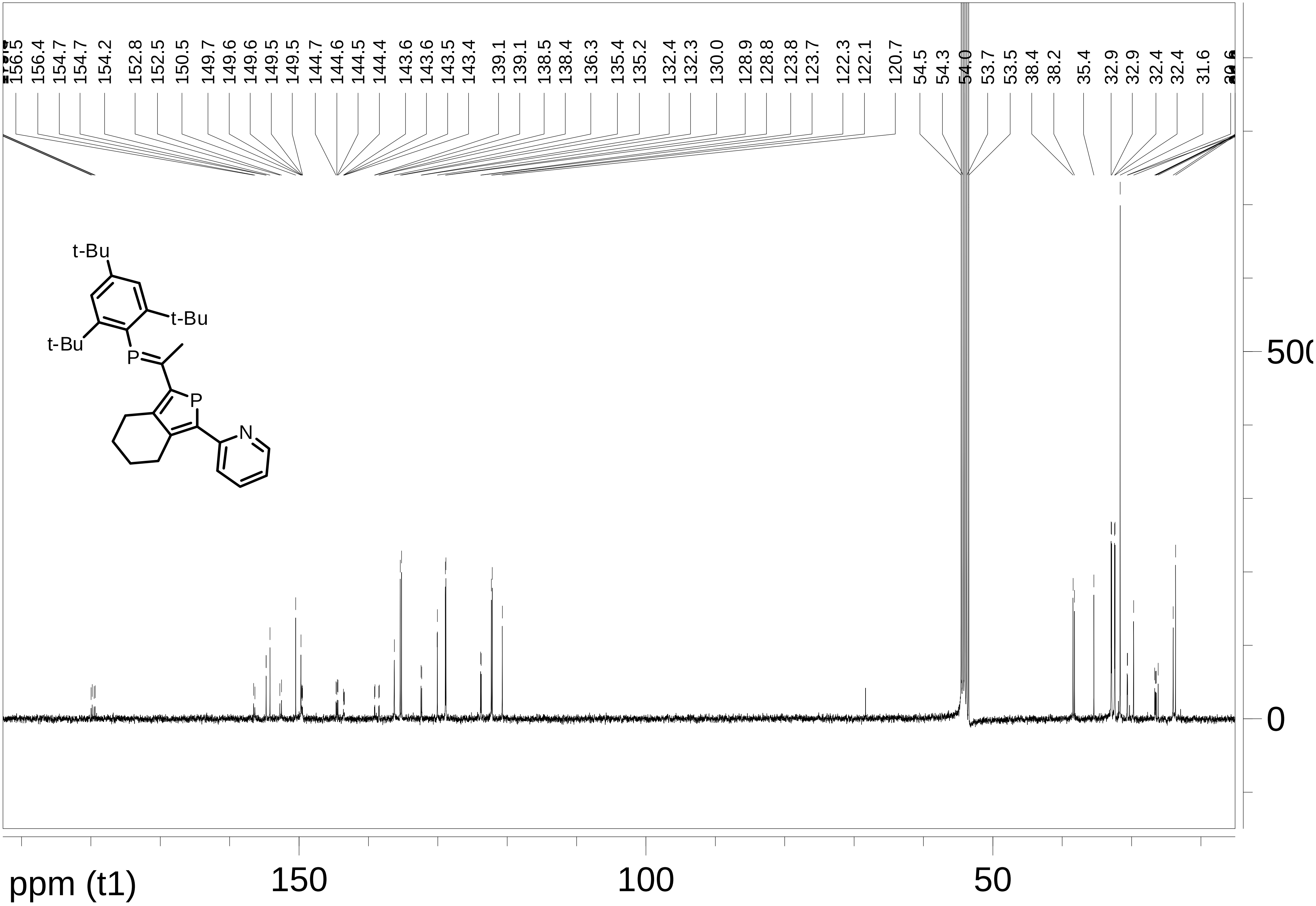

156.5
156.4
154.7
154.7
154.2
152.8
152.5
150.5
149.7
149.6
149.6
149.5
149.5
144.7
144.6
144.5
144.4
143.6
143.6
143.5
143.4
139.1
139.1
138.5
138.4
136.3
135.4
135.2
132.4
132.3
130.0
128.9
128.8
123.8
123.7
122.3
122.1
120.7
54.5
54.3
54.0
53.7
53.5
38.4
38.2
35.4
32.9
32.9
32.4
32.4
31.6
t-Bu
t-Bu
t-Bu
P
P
N
500
0
ppm (t1)
150
100
50

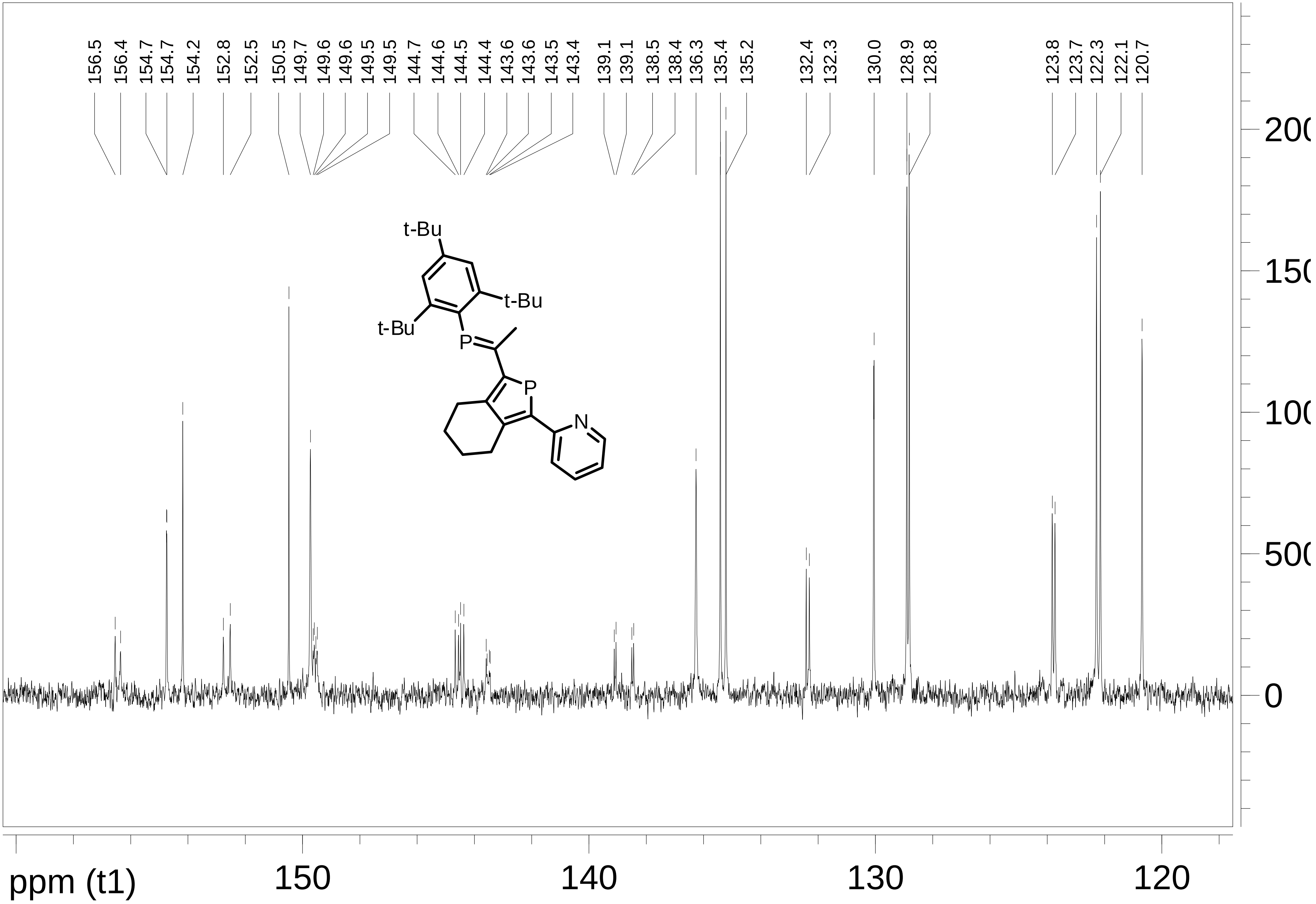
156.5
156.4
154.7
154.7
154.2
152.8
152.5
150.5
149.7
149.6
149.6
149.5
149.5
144.7
144.6
144.5
144.4
143.6
143.6
143.5
143.4
139.1
139.1
138.5
138.4
136.3
135.4
135.2
132.4
132.3
130.0
128.9
128.8
123.8
123.7
122.3
122.1
120.7
t-Bu
t-Bu
t-Bu
P
P
N
200
150
100
500
0
ppm (t1)
150
140
130
120

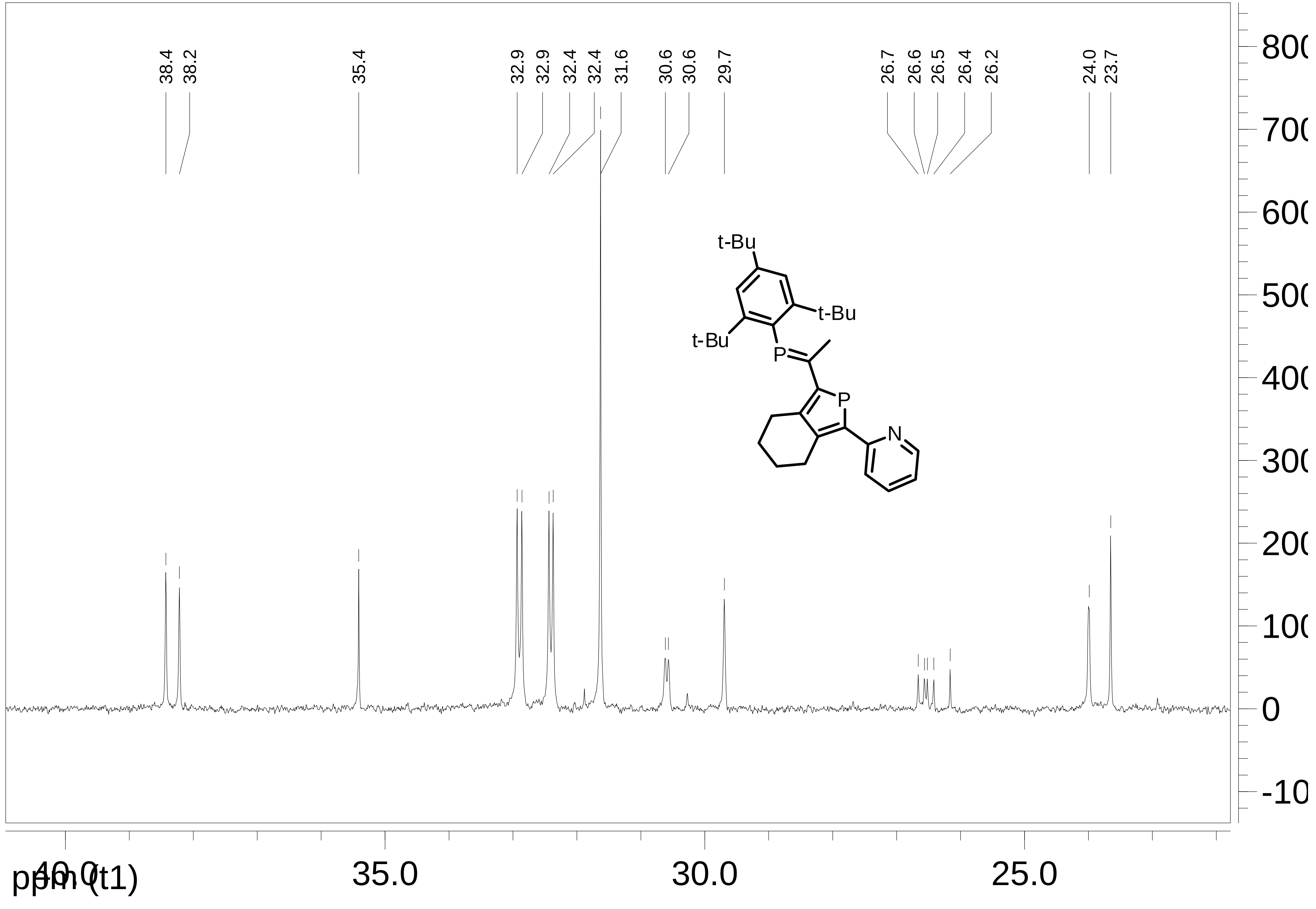

38.4
38.2
35.4
32.9
32.9
32.4
32.4
31.6
30.6
30.6
29.7
26.7
26.6
26.5
26.4
26.2
24.0
23.7
t-Bu
t-Bu
t-Bu
P
P
N
800
700
600
500
400
300
200
100
0
-10
40.0
35.0
30.0
25.0
ppm (t1)

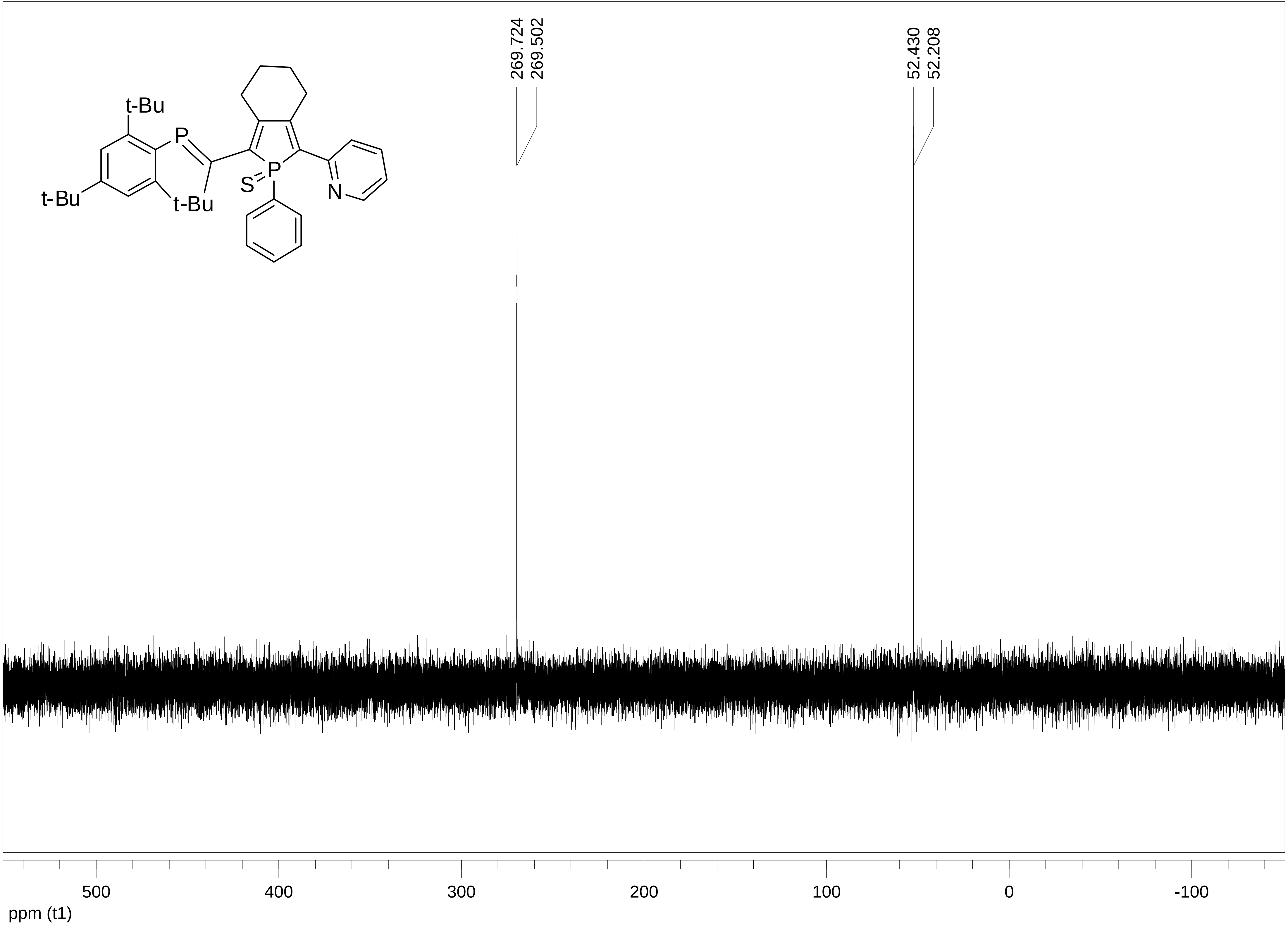

269.724
269.502
52.430
52.208
t-Bu
t-Bu
t-Bu
P
S
P
N
500
400
300
200
100
0
-100
ppm (t1)

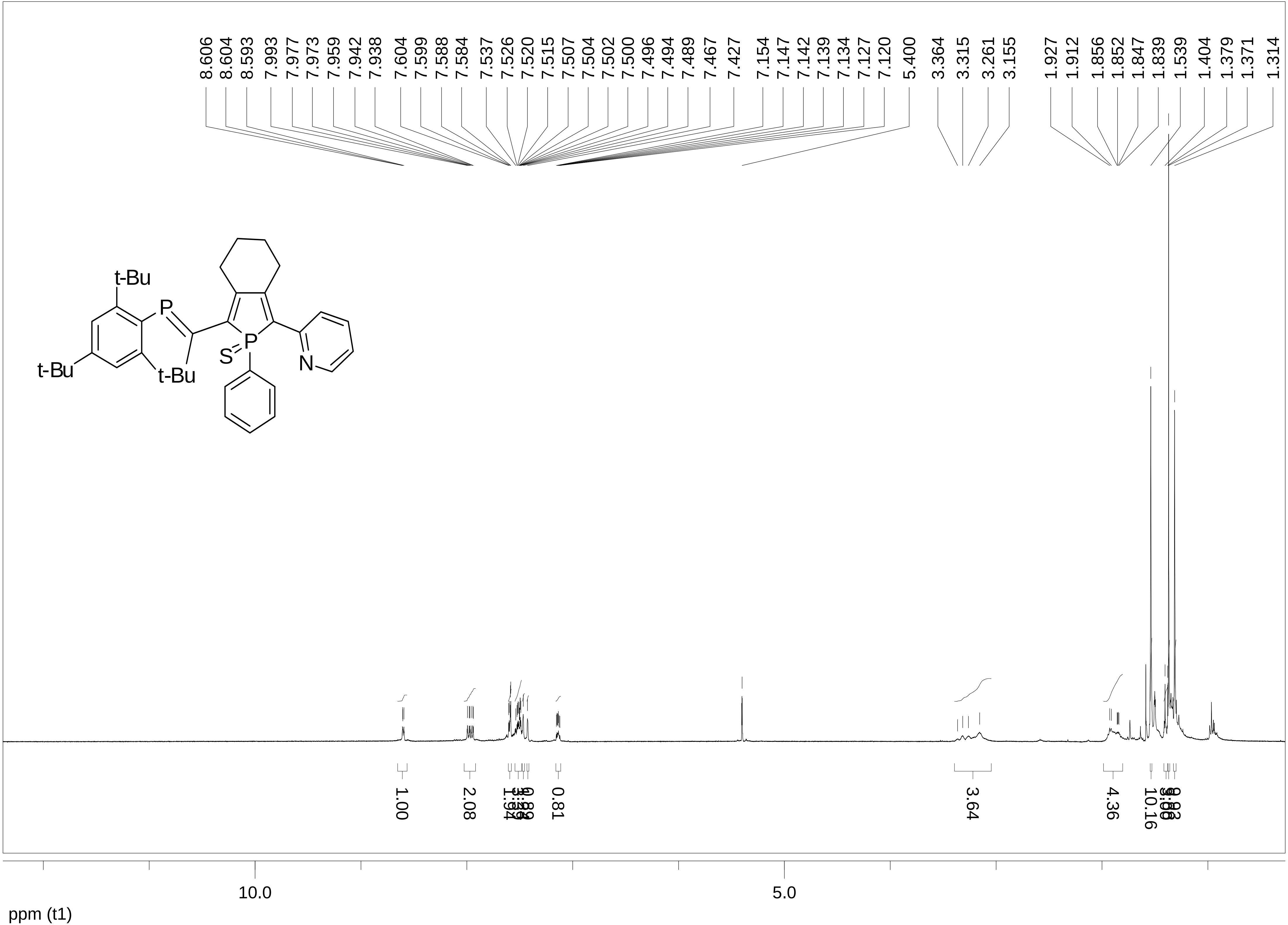
8.606
8.604
8.593
7.993
7.977
7.973
7.959
7.942
7.938
7.604
7.599
7.588
7.584
7.537
7.526
7.520
7.515
7.507
7.504
7.502
7.500
7.496
7.494
7.489
7.467
7.427
7.154
7.147
7.142
7.139
7.134
7.127
7.120
5.400
3.364
3.315
3.261
3.155
1.927
1.912
1.856
1.852
1.847
1.839
1.539
1.404
1.379
1.371
1.314
t-Bu
P
t-Bu
t-Bu
S
P
N
1.00
2.08
0.81
3.64
4.36
10.16
10.0
5.0
ppm (t1)

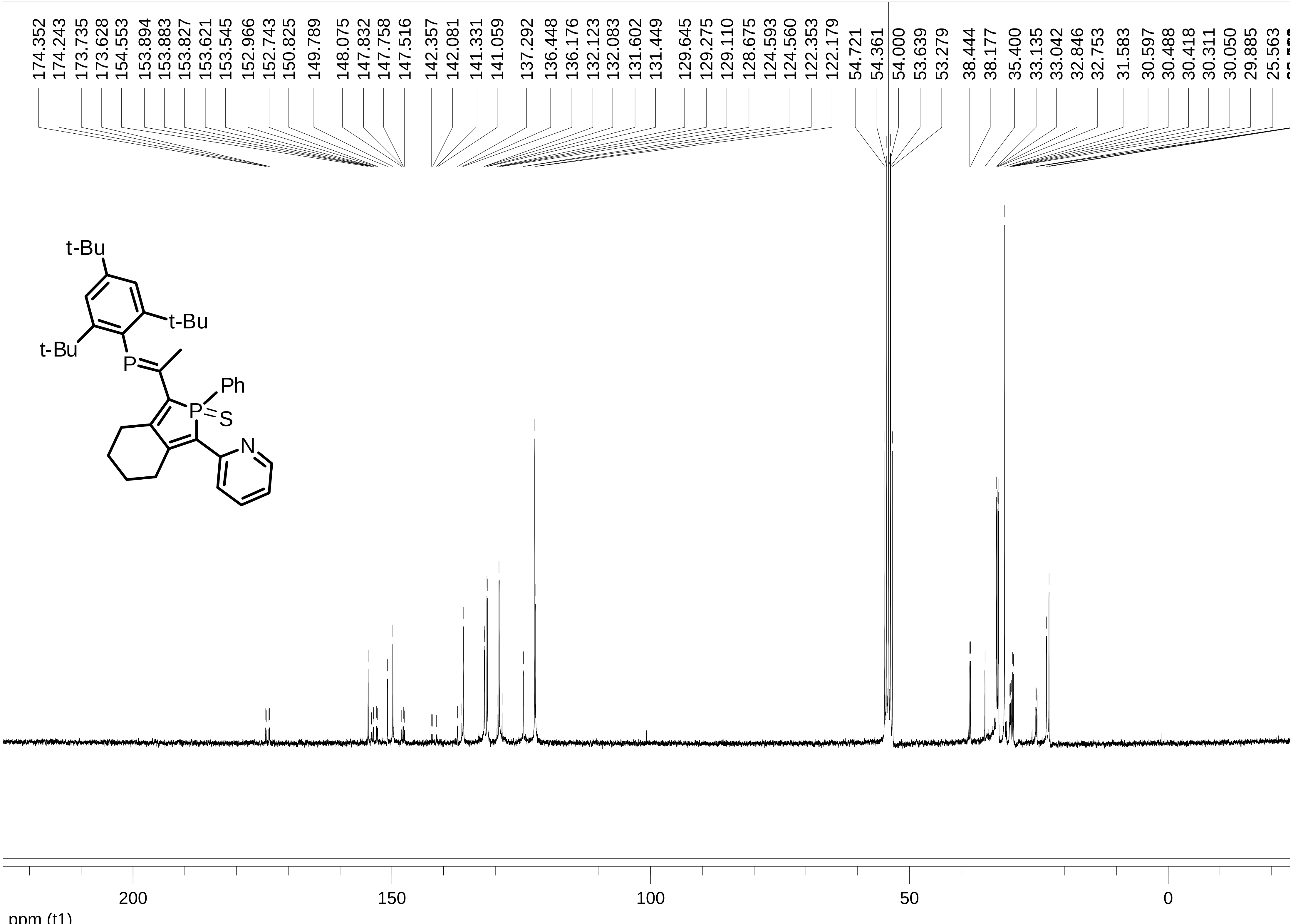
174.352
174.243
173.735
173.628
154.553
153.894
153.883
153.827
153.621
153.545
152.966
152.743
150.825
149.789
148.075
147.832
147.758
147.516
142.357
142.081
141.331
141.059
137.292
136.448
136.176
132.123
132.083
131.602
131.449
129.645
129.275
129.110
128.675
124.593
124.560
122.353
122.179
54.721
54.361
54.000
53.639
53.279
38.444
38.177
35.400
33.135
33.042
32.846
32.753
31.583
30.597
30.488
30.418
30.311
30.050
29.885
25.563
t-Bu
t-Bu
t-Bu
P
Ph
P
S
N
200
150
100
50
0
ppm (t1)

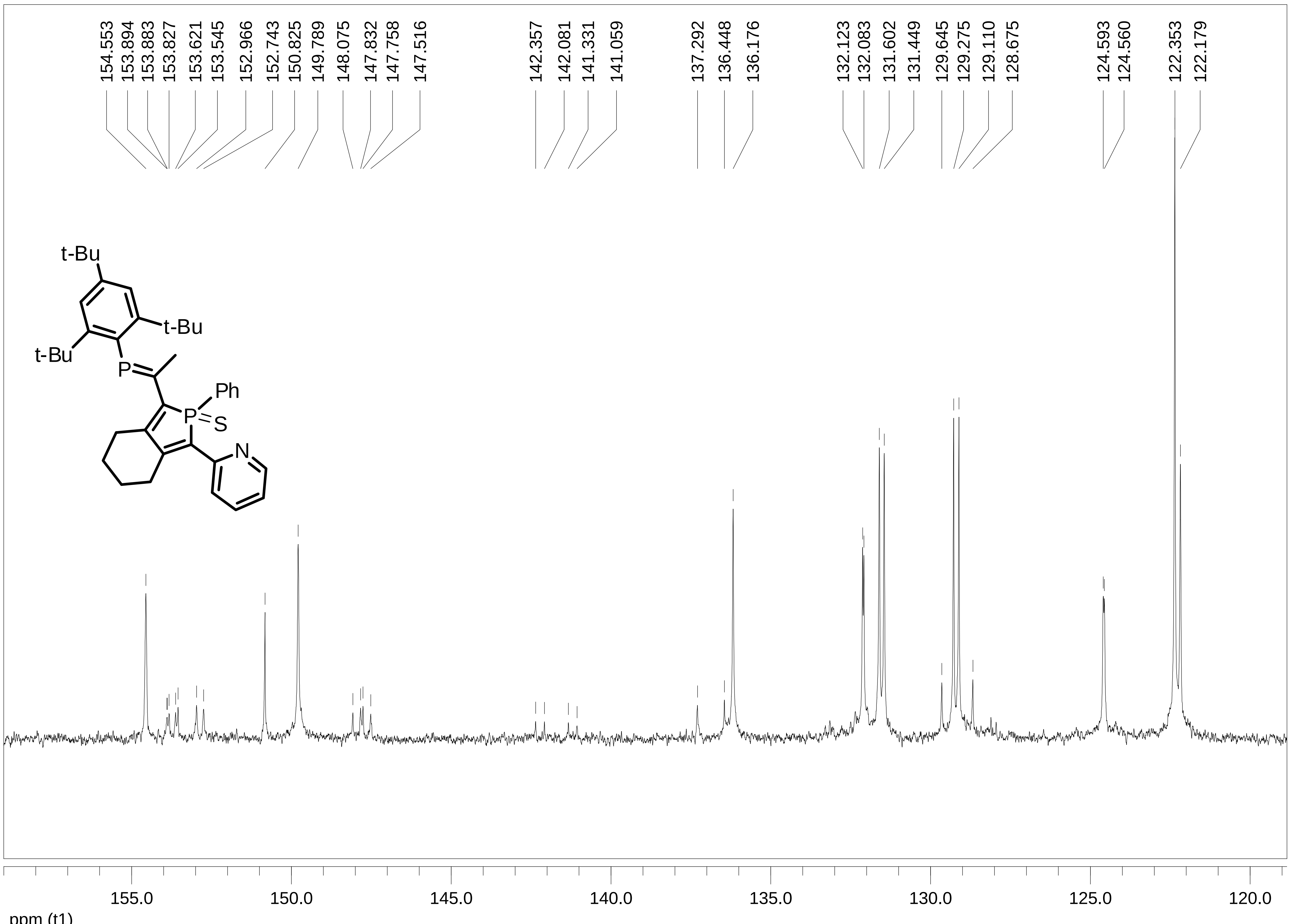
154.553
153.894
153.883
153.827
153.621
153.545
152.966
152.743
150.825
149.789
148.075
147.832
147.758
147.516
142.357
142.081
141.331
141.059
137.292
136.448
136.176
132.123
132.083
131.602
131.449
129.645
129.275
129.110
128.675
124.593
124.560
122.353
122.179
t-Bu
t-Bu
t-Bu
P
Ph
P
S
N
155.0
150.0
145.0
140.0
135.0
130.0
125.0
120.0
ppm (t1)

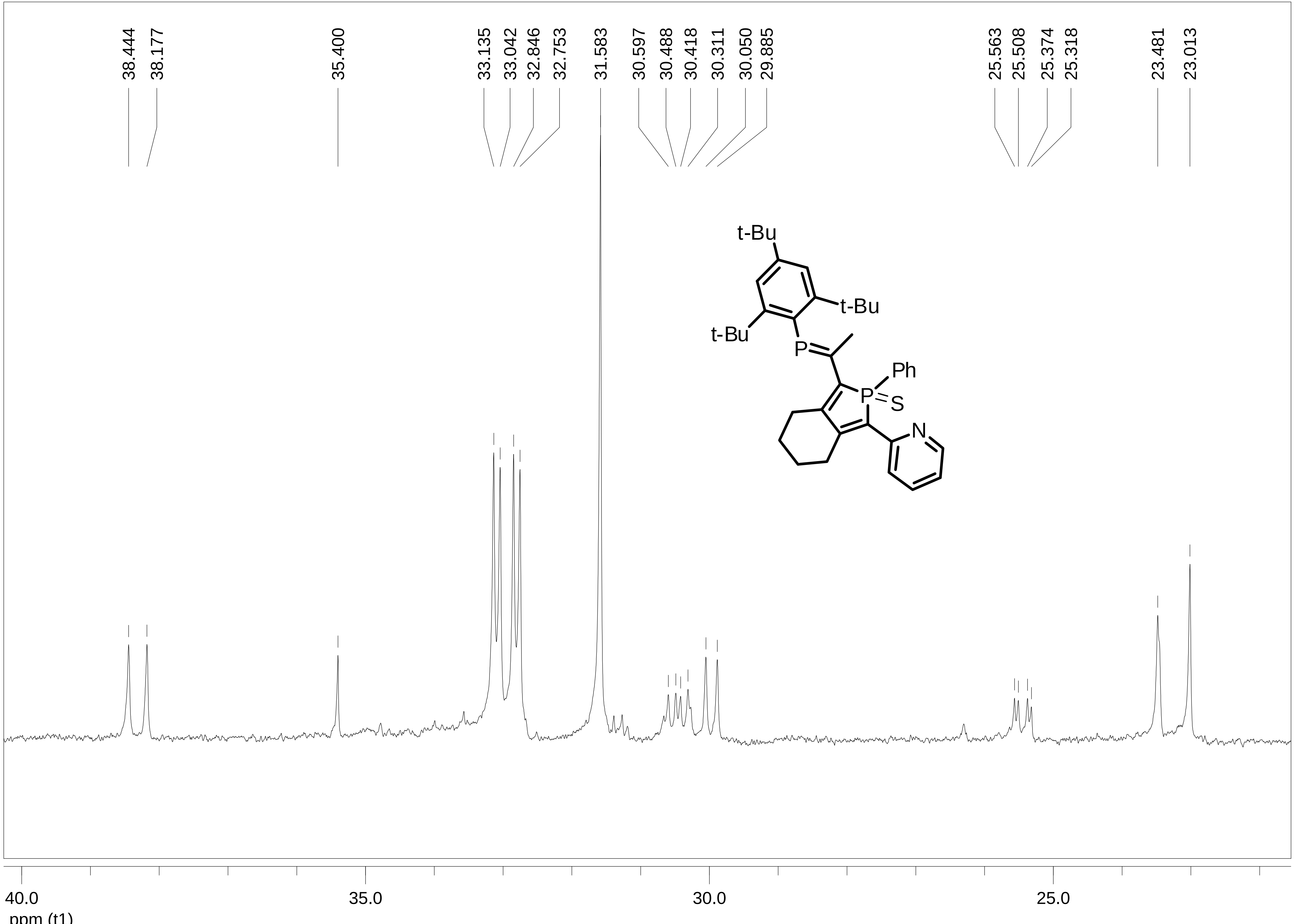

38.444
38.177
35.400
33.135
33.042
32.846
32.753
31.583
30.597
30.488
30.418
30.311
30.050
29.885
25.563
25.508
25.374
25.318
23.481
23.013
t-Bu
t-Bu
t-Bu
P
Ph
P
S
N
40.0
35.0
30.0
25.0
ppm (t1)

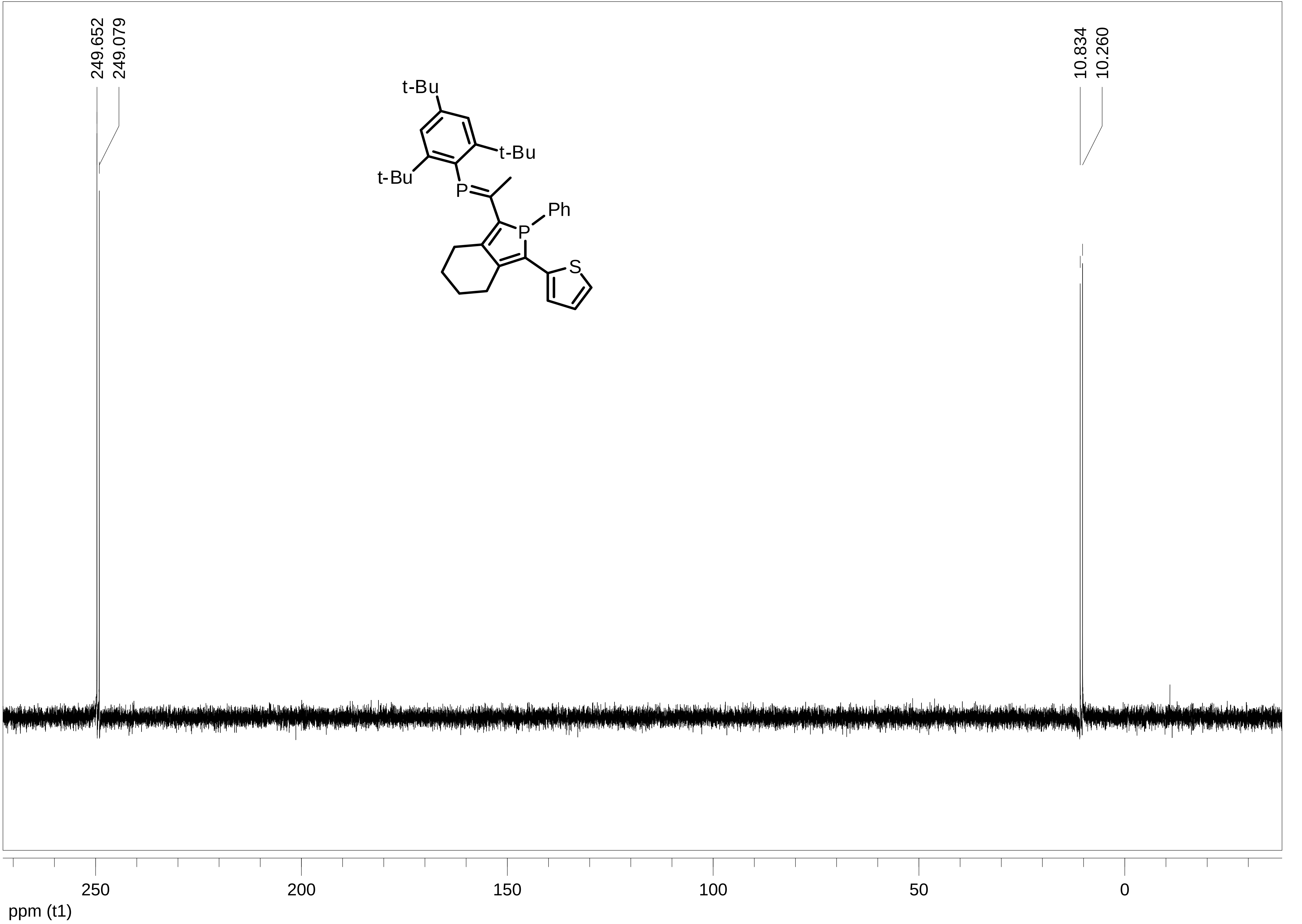
249.652
249.079
10.834
10.260
t-Bu
t-Bu
t-Bu
P
Ph
P
S
250
200
150
100
50
0
ppm (t1)

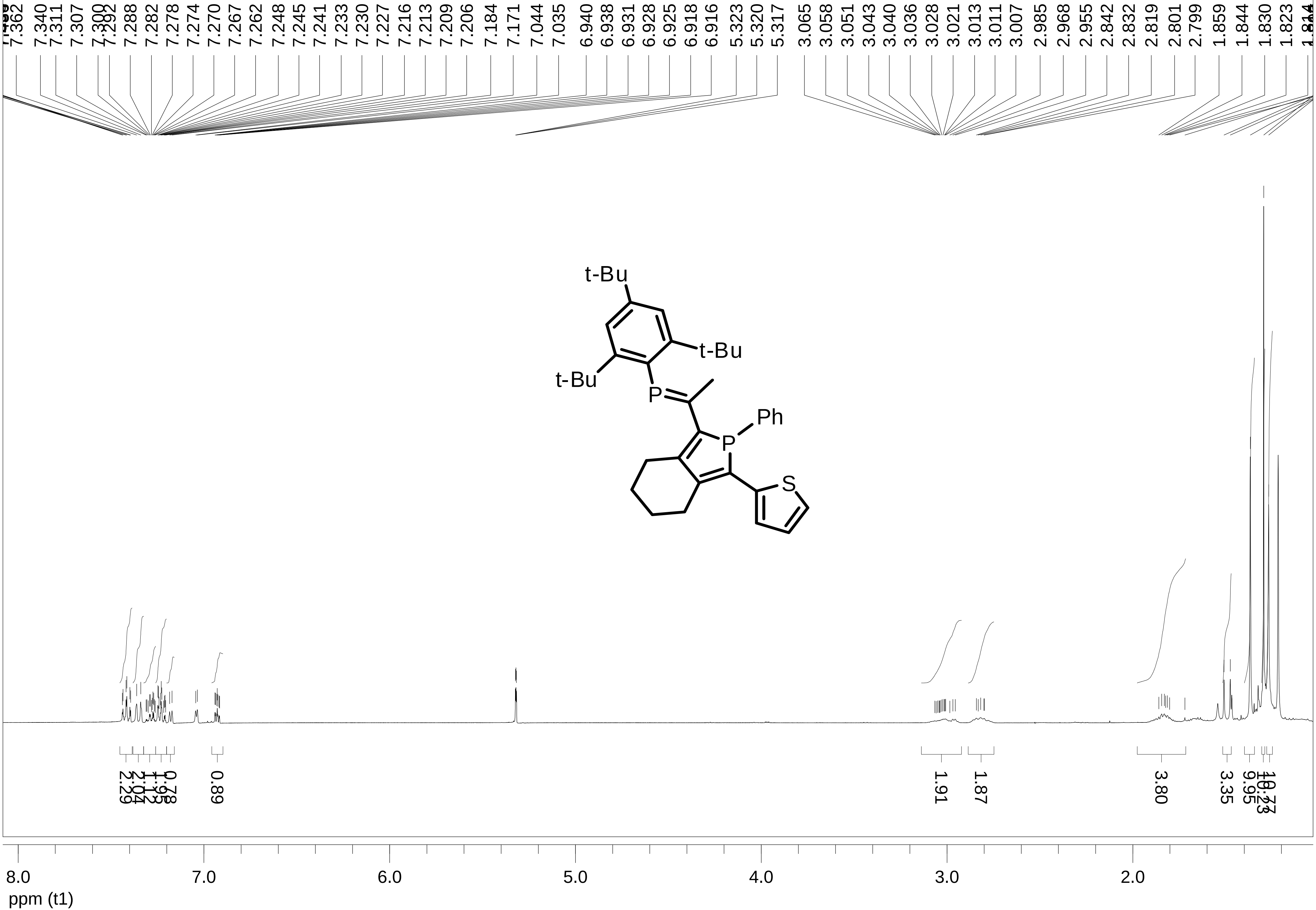

7.362
7.340
7.311
7.307
7.292
7.288
7.282
7.278
7.274
7.270
7.267
7.262
7.248
7.245
7.241
7.233
7.230
7.227
7.216
7.213
7.209
7.206
7.184
7.171
7.044
7.035
6.940
6.938
6.931
6.928
6.925
6.918
6.916
5.323
5.320
5.317
3.065
3.058
3.051
3.043
3.040
3.036
3.028
3.021
3.013
3.011
3.007
2.985
2.968
2.955
2.842
2.832
2.819
2.801
2.799
1.859
1.844
1.830
1.823
t-Bu
t-Bu
t-Bu
P
P
Ph
S
2.29
0.89
1.91
1.87
3.80
3.35
9.95
8.0
7.0
6.0
5.0
4.0
3.0
2.0
ppm (t1)

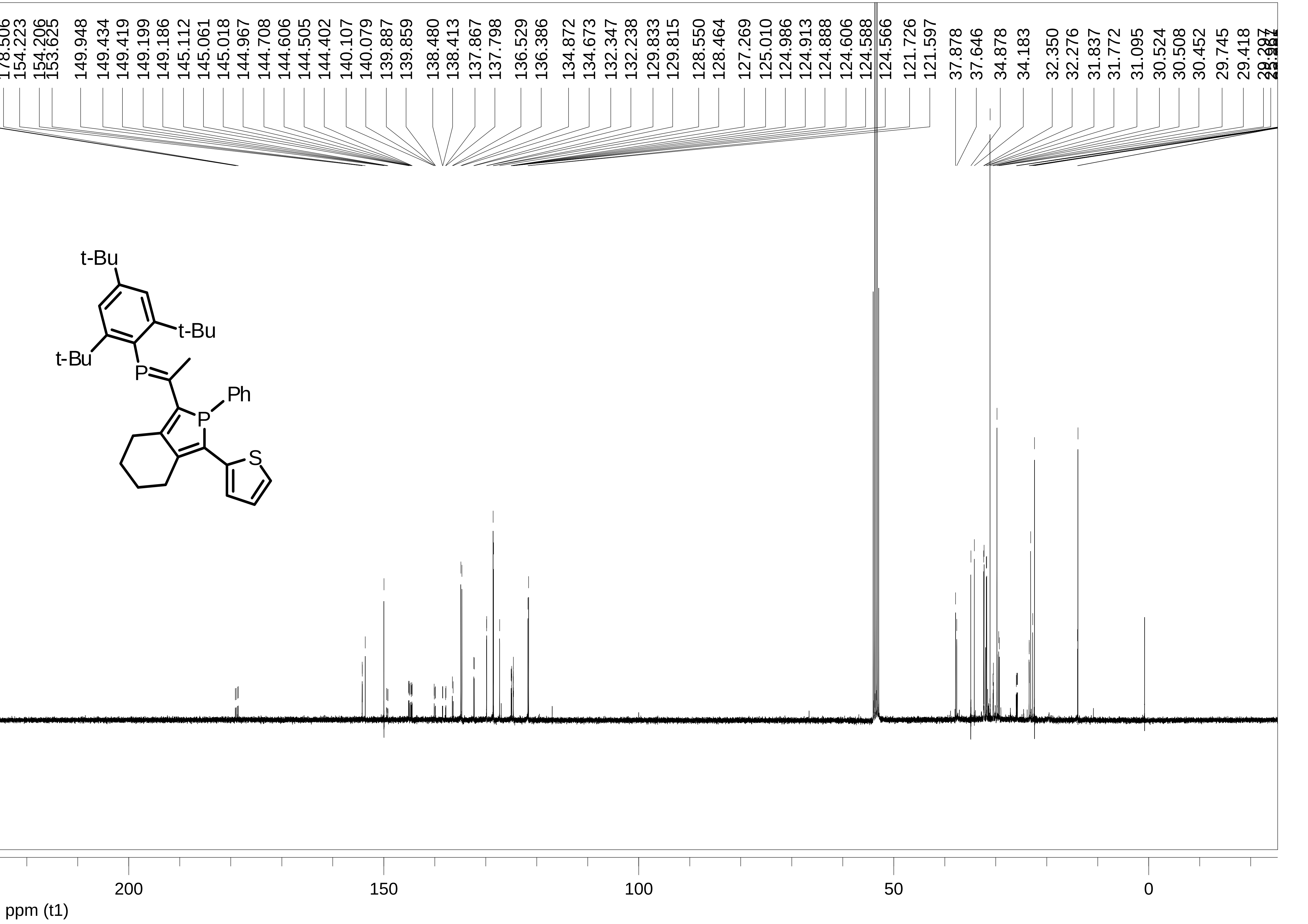

178.506
154.223
154.206
153.625
149.948
149.434
149.419
149.199
149.186
145.112
145.061
145.018
144.967
144.708
144.606
144.505
144.402
140.107
140.079
139.887
139.859
138.480
138.413
137.867
137.798
136.529
136.386
134.872
134.673
132.347
132.238
129.833
129.815
128.550
128.464
127.269
125.010
124.986
124.913
124.888
124.606
124.588
124.566
121.726
121.597
37.878
37.646
34.878
34.183
32.350
32.276
31.837
31.772
31.095
30.524
30.508
30.452
29.745
29.418
t-Bu
t-Bu
t-Bu
P
Ph
P
S
200
150
100
50
0
ppm (t1)

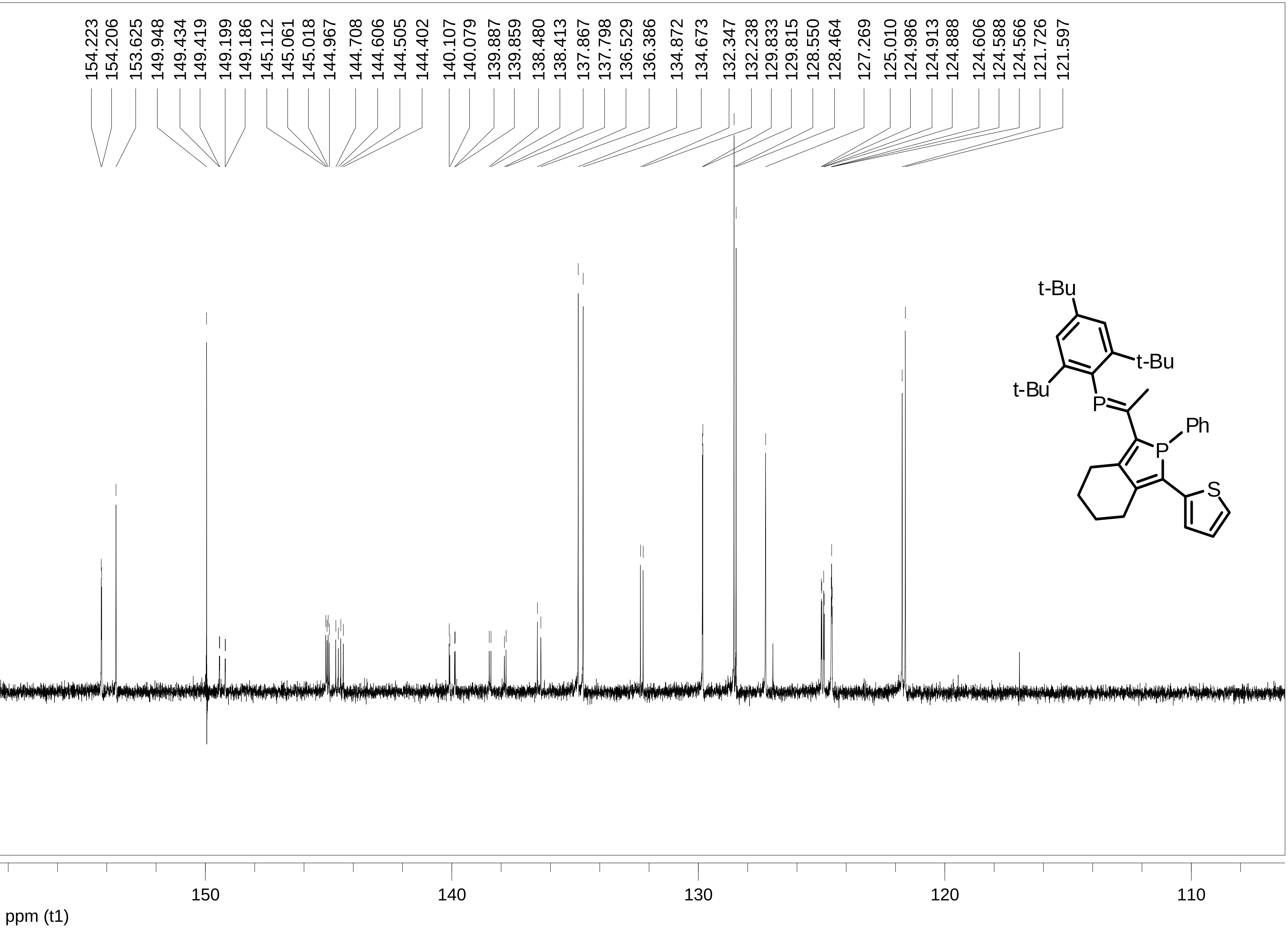
154.223
154.206
153.625
149.948
149.434
149.419
149.199
149.186
145.112
145.061
145.018
144.967
144.708
144.606
144.505
144.402
140.107
140.079
139.887
139.859
138.480
138.413
137.867
137.798
136.529
136.386
134.872
134.673
132.347
132.238
129.833
129.815
128.550
128.464
127.269
125.010
124.986
124.913
124.888
124.606
124.588
124.566
121.726
121.597
t-Bu
t-Bu
t-Bu
P
Ph
P
S
150
140
130
120
110
ppm (t1)

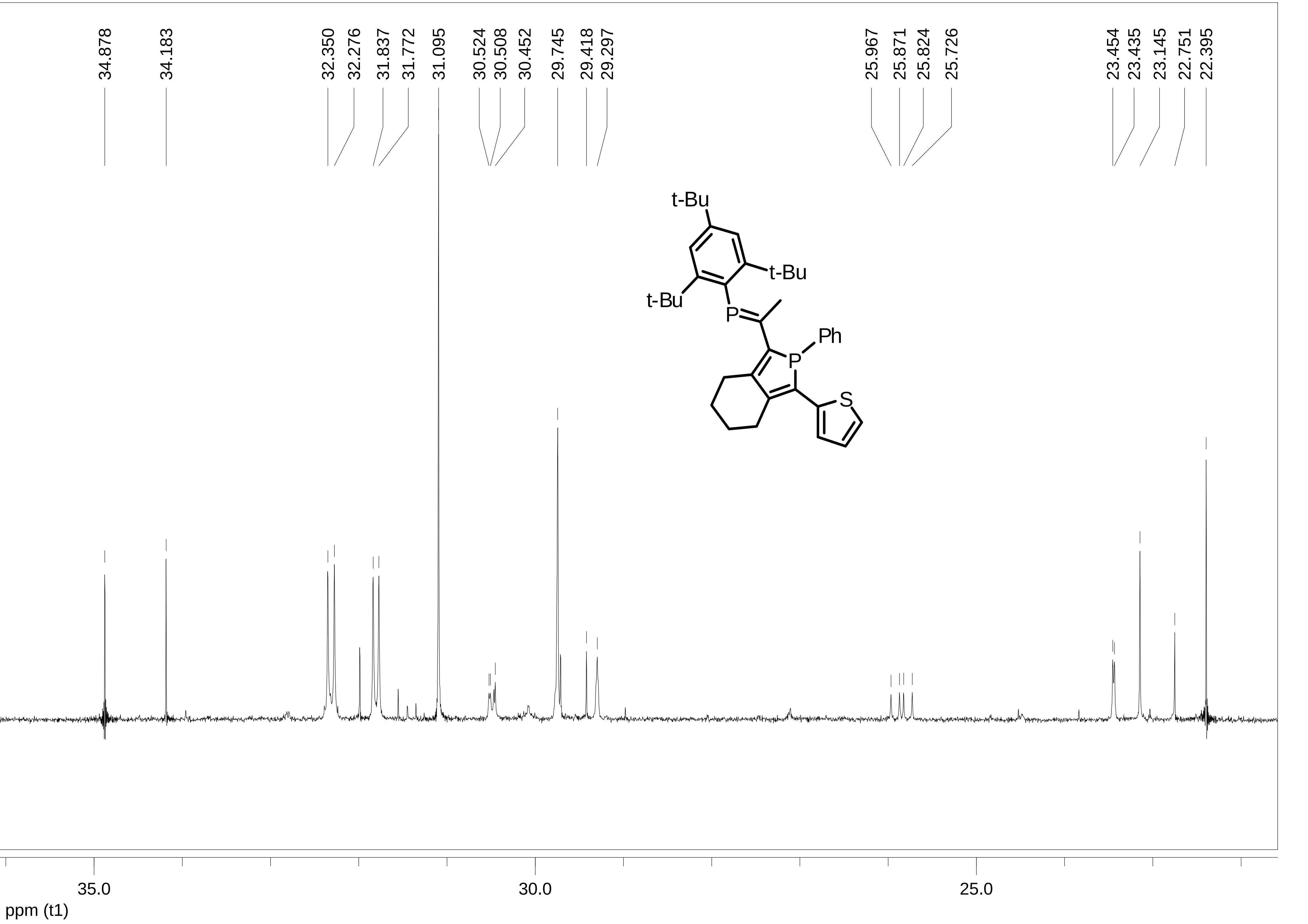

34.878
34.183
32.350
32.276
31.837
31.772
31.095
30.524
30.508
30.452
29.745
29.418
29.297
25.967
25.871
25.824
25.726
23.454
23.435
23.145
22.751
22.395
t-Bu
t-Bu
t-Bu
P
Ph
P
S
35.0
30.0
25.0
ppm (t1)

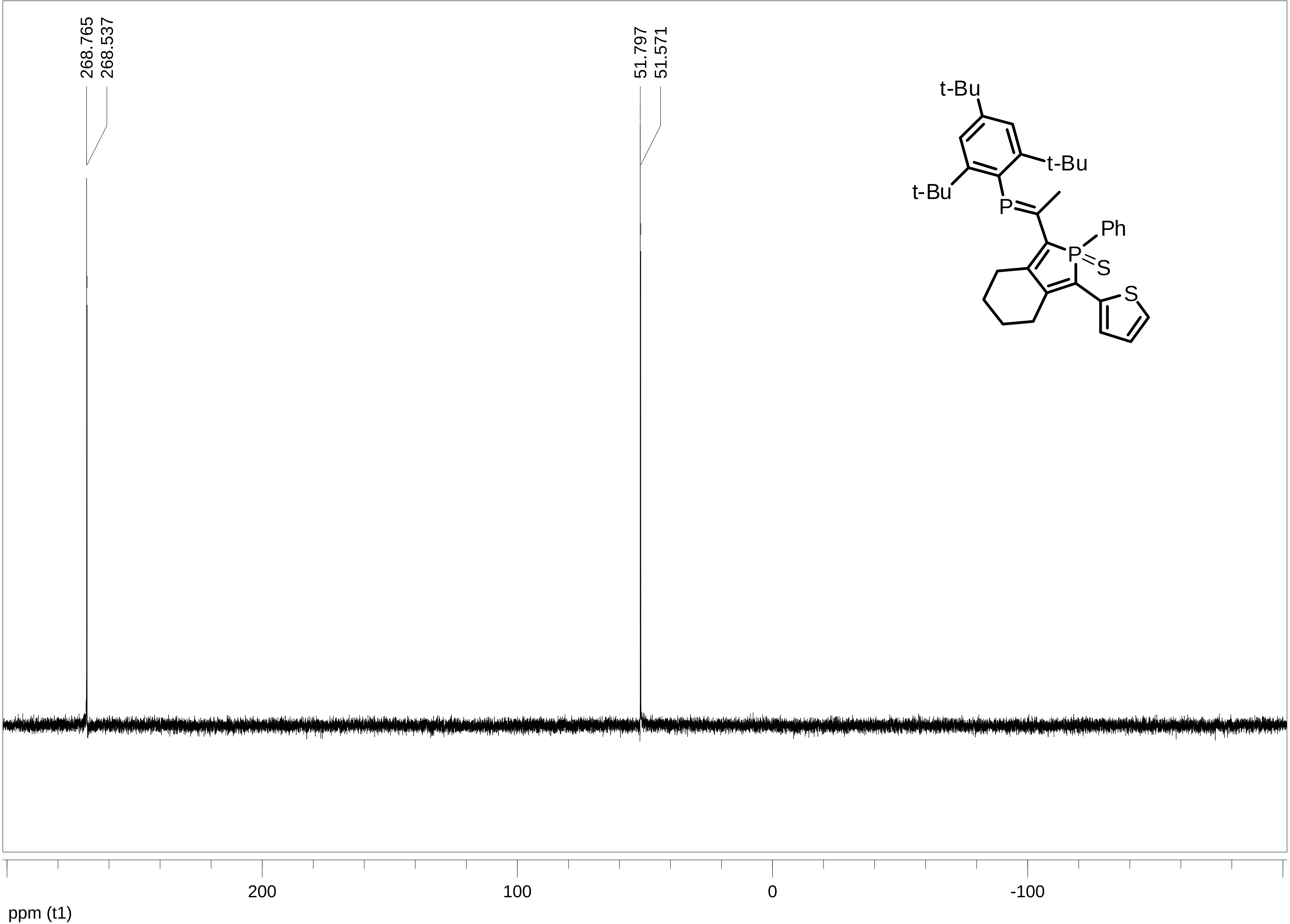
268.765
268.537
51.797
51.571
t-Bu
t-Bu
t-Bu
P
Ph
P
S
S
200
100
0
-100
ppm (t1)

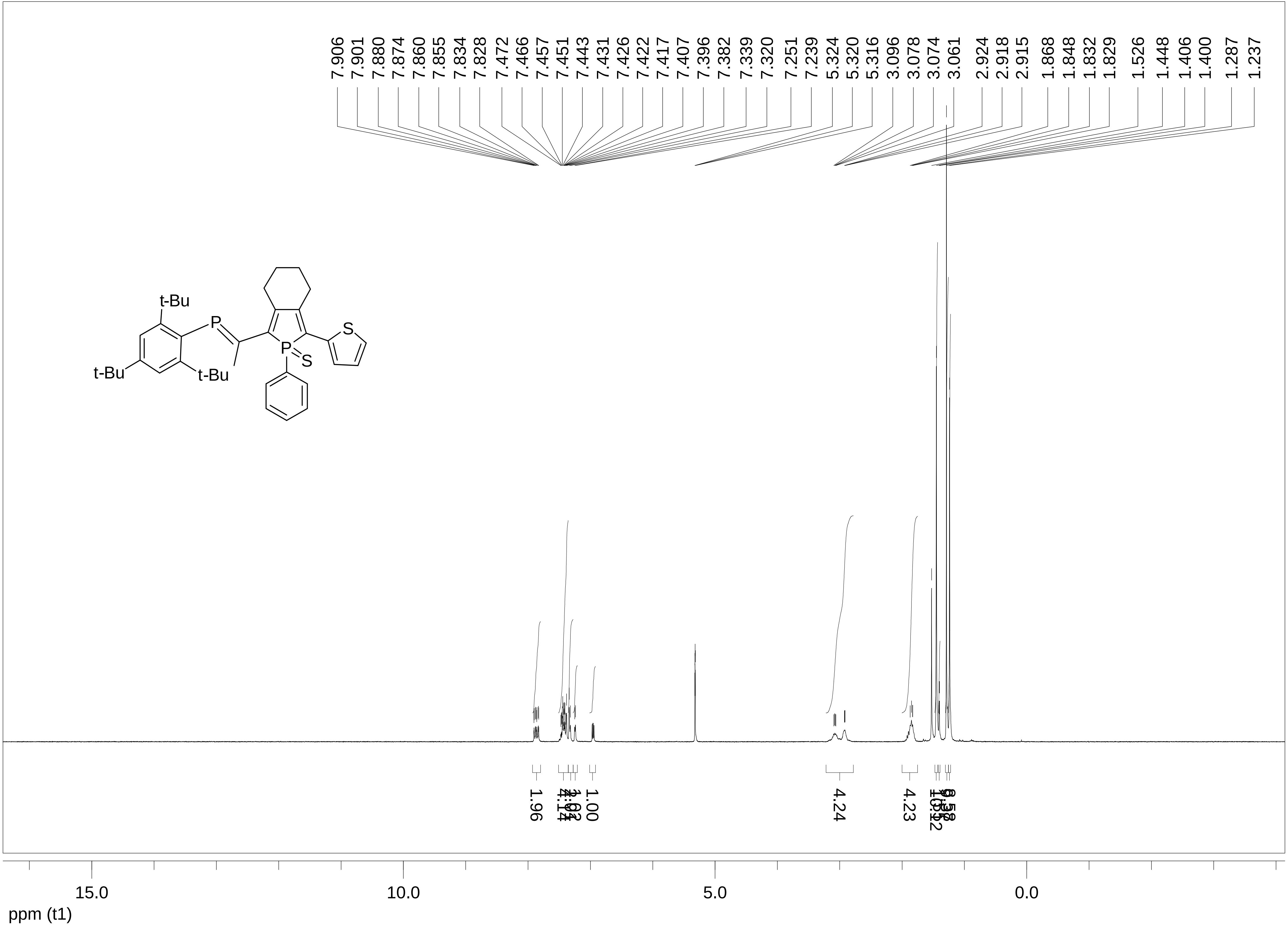

7.906
7.901
7.880
7.874
7.860
7.855
7.834
7.828
7.472
7.466
7.457
7.451
7.443
7.431
7.426
7.422
7.417
7.407
7.396
7.382
7.339
7.320
7.251
7.239
5.324
5.320
5.316
3.096
3.078
3.074
3.061
2.924
2.918
2.915
1.868
1.848
1.832
1.829
1.526
1.448
1.406
1.400
1.287
1.237
t-Bu
t-Bu
t-Bu
P
P
S
S
1.96
1.00
4.24
4.23
15.0
10.0
5.0
0.0
ppm (t1)

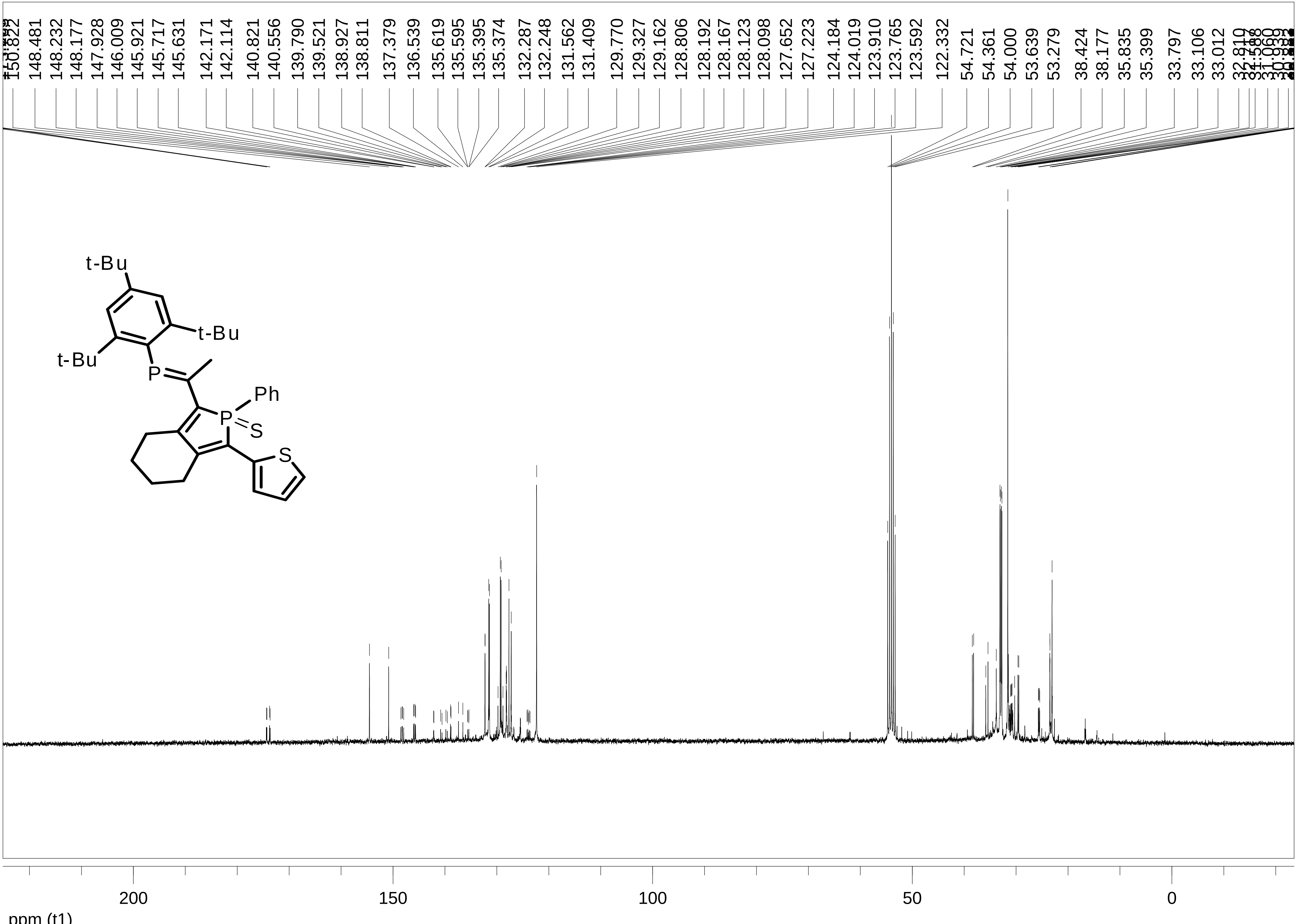

150.822
148.481
148.232
148.177
147.928
146.009
145.921
145.717
145.631
142.171
142.114
140.821
140.556
139.790
139.521
138.927
138.811
137.379
136.539
135.619
135.595
135.395
135.374
132.287
132.248
131.562
131.409
129.770
129.327
129.162
128.806
128.192
128.167
128.123
128.098
127.652
127.223
124.184
124.019
123.910
123.765
123.592
122.332
54.721
54.361
54.000
53.639
53.279
38.424
38.177
35.835
35.399
33.797
33.106
33.012
t-Bu
t-Bu
t-Bu
P
Ph
P
S
S
200
150
100
50
0
ppm (t1)

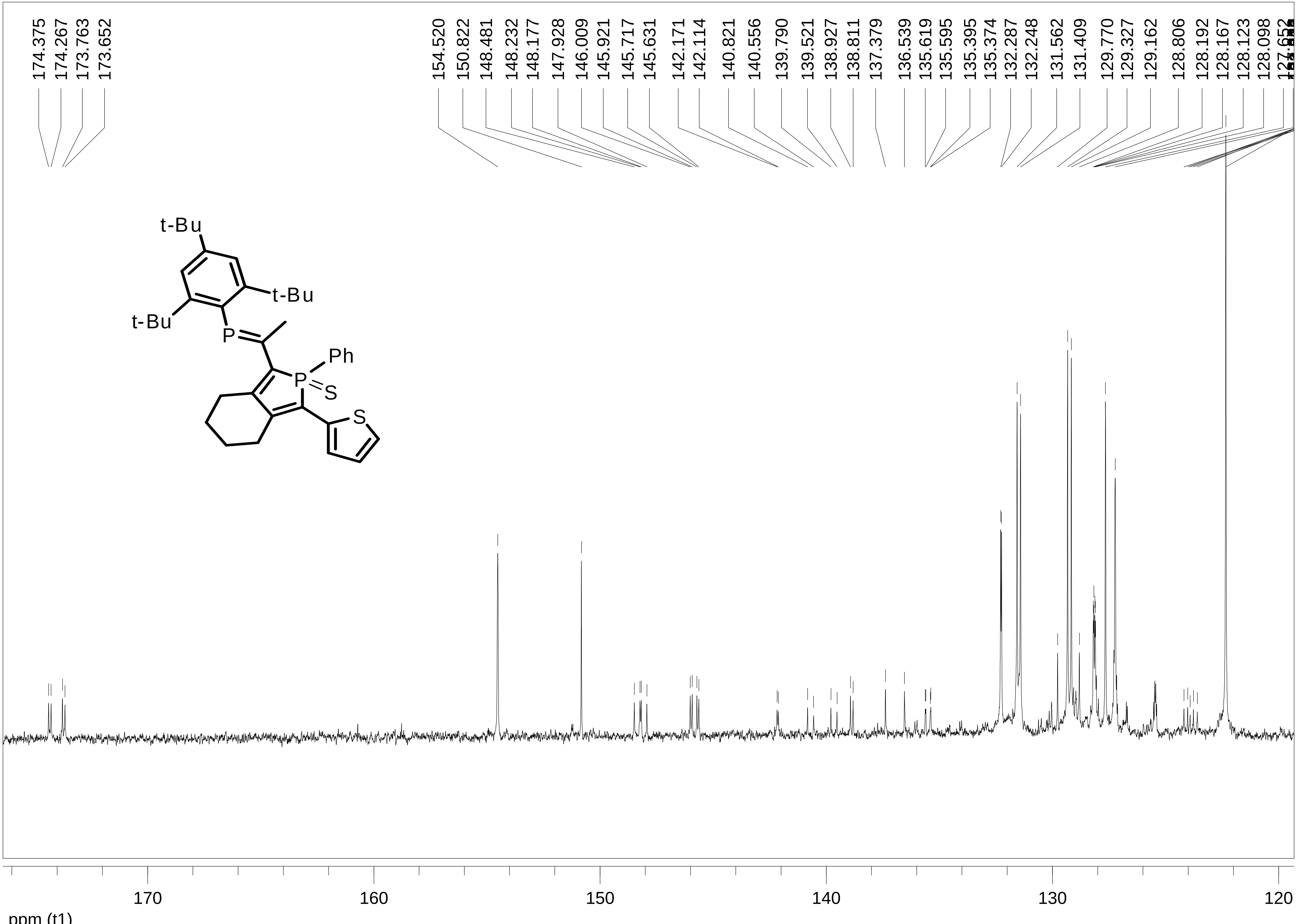

174.375
174.267
173.763
173.652
154.520
150.822
148.481
148.232
148.177
147.928
146.009
145.921
145.717
145.631
142.171
142.114
140.821
140.556
139.790
139.521
138.927
138.811
137.379
136.539
135.619
135.595
135.395
135.374
132.287
132.248
131.562
131.409
129.770
129.327
129.162
128.806
128.192
128.167
128.123
128.098
127.652
t-Bu
t-Bu
t-Bu
P
Ph
P
S
S
170
160
150
140
130
120
ppm (t1)

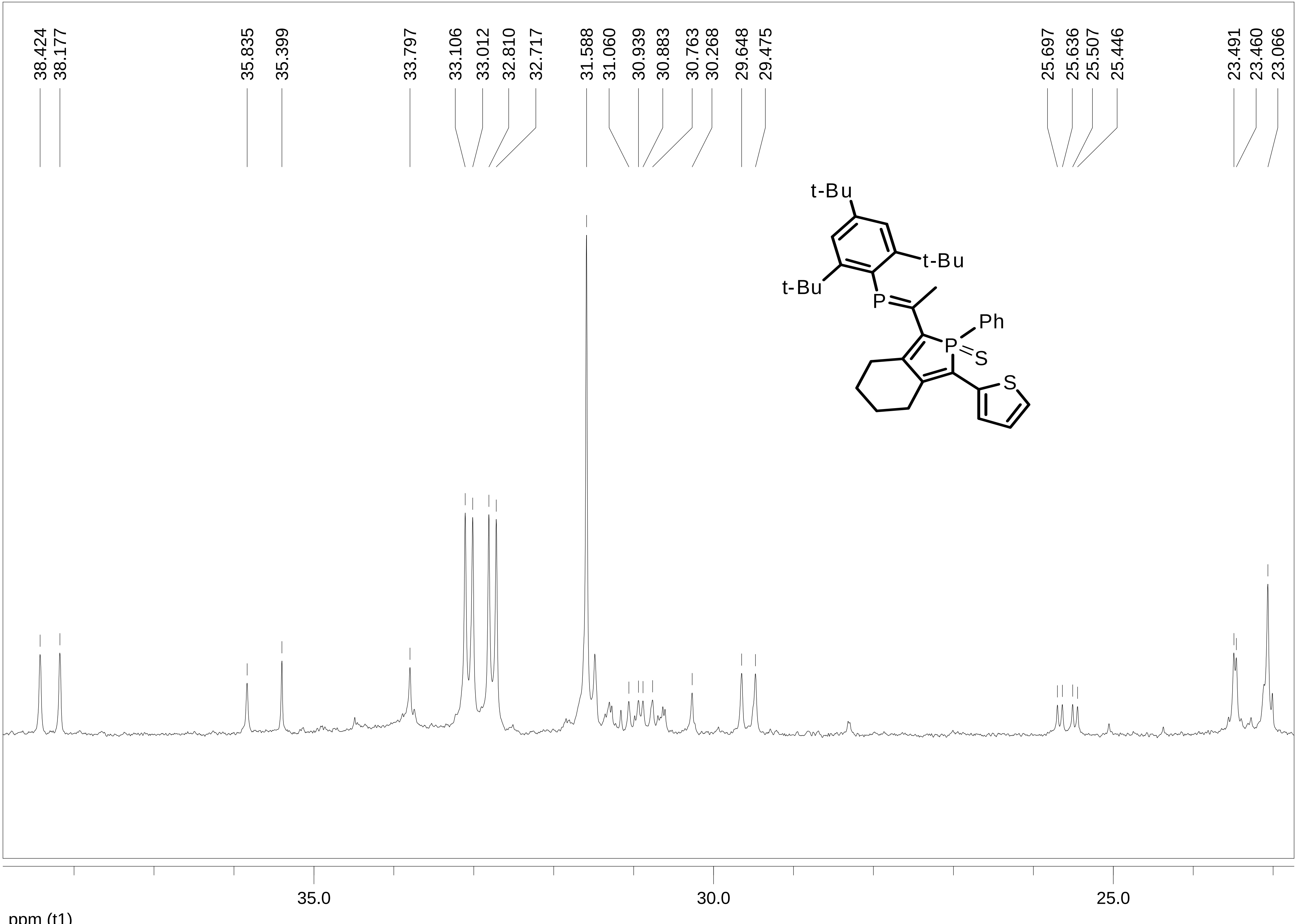
38.424
38.177
35.835
35.399
33.797
33.106
33.012
32.810
32.717
31.588
31.060
30.939
30.883
30.763
30.268
29.648
29.475
25.697
25.636
25.507
25.446
23.491
23.460
23.066
t-Bu
t-Bu
t-Bu
P
Ph
P
S
S
35.0
30.0
25.0
ppm (t1)

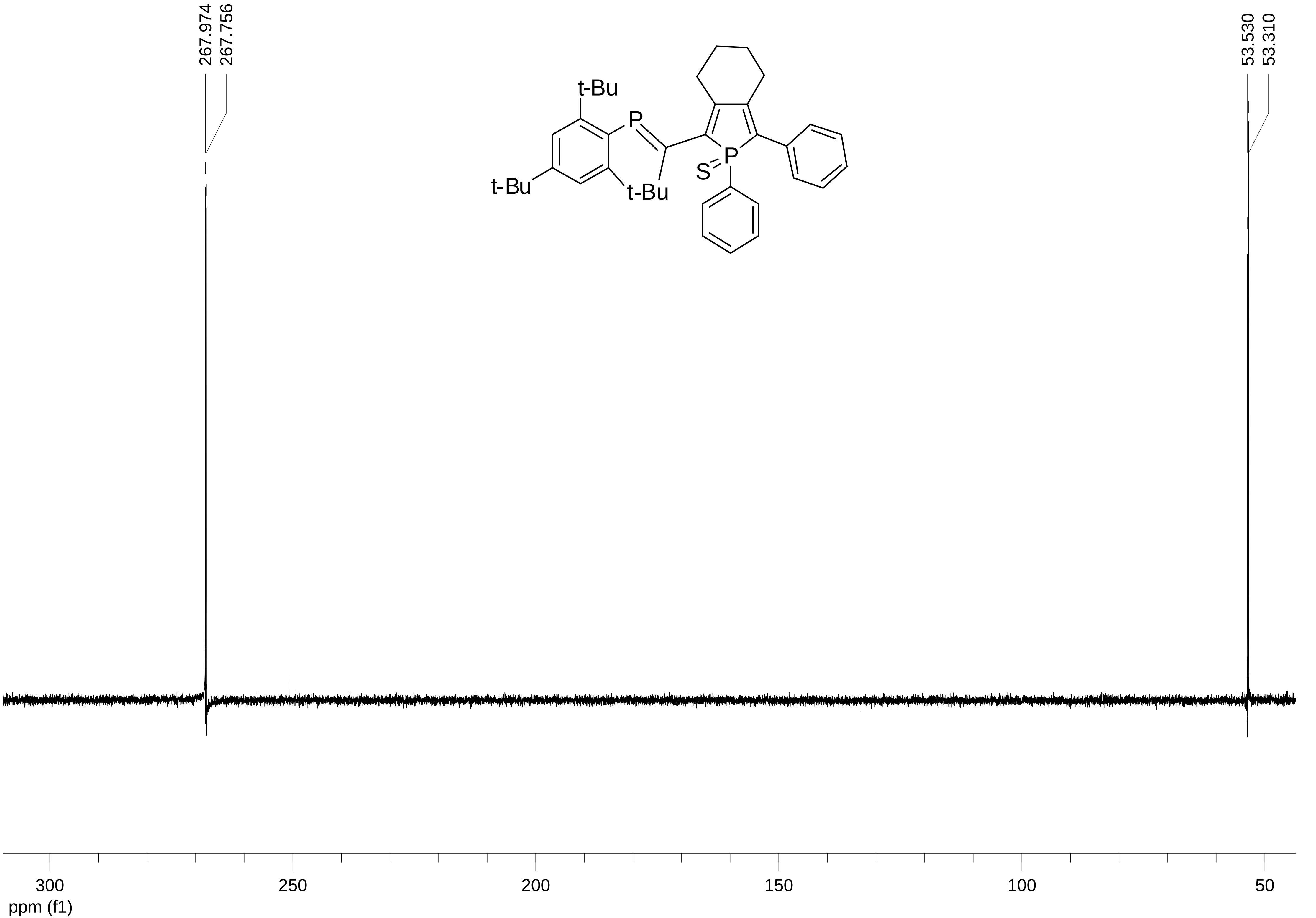
267.974
267.756
53.530
53.310
t-Bu
t-Bu
t-Bu
P
P
S
300
250
200
150
100
50
ppm (f1)

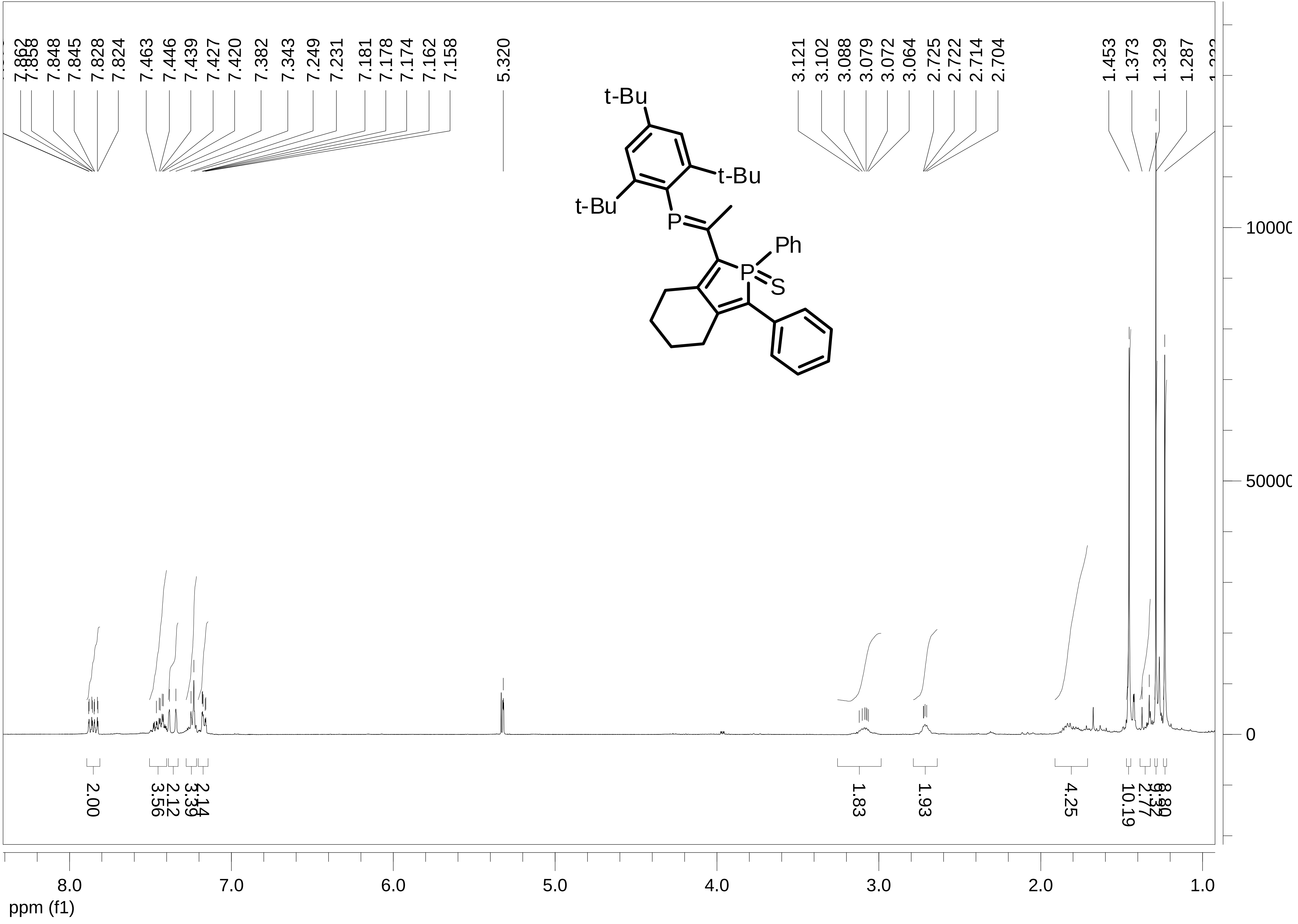
7.858
7.848
7.845
7.828
7.824
7.463
7.446
7.439
7.427
7.420
7.382
7.343
7.249
7.231
7.181
7.178
7.174
7.162
7.158
5.320
3.121
3.102
3.088
3.079
3.072
3.064
2.725
2.722
2.714
2.704
1.453
1.373
1.329
1.287
t-Bu
t-Bu
t-Bu
P
Ph
P
S
2.00
3.56
2.12
3.39
1.83
1.93
4.25
10.19
2.77
0
50000
8.0
7.0
6.0
5.0
4.0
3.0
2.0
1.0
ppm (f1)

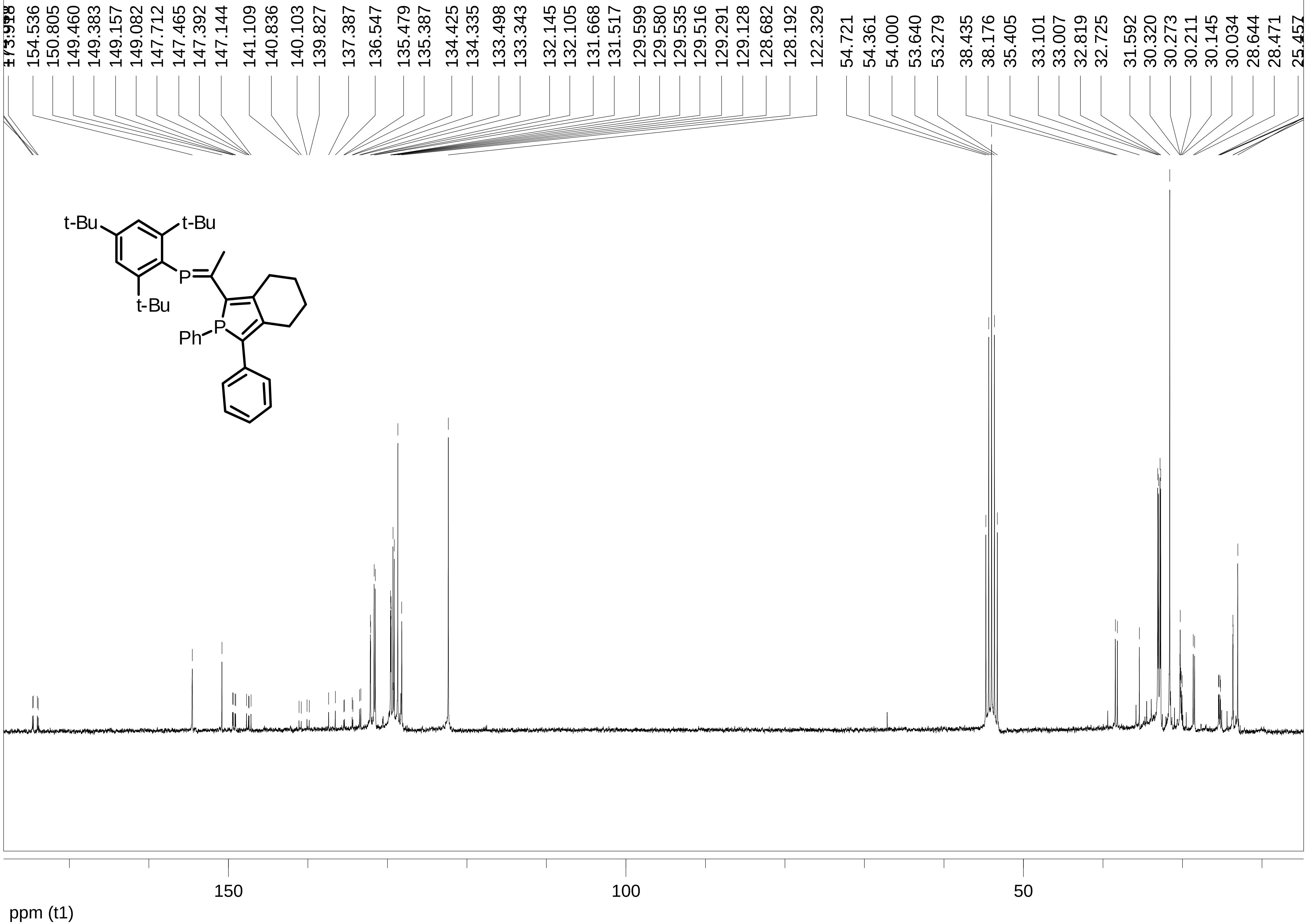

154.536
150.805
149.460
149.383
149.157
149.082
147.712
147.465
147.392
147.144
141.109
140.836
140.103
139.827
137.387
136.547
135.479
135.387
134.425
134.335
133.498
133.343
132.145
132.105
131.668
131.517
129.599
129.580
129.535
129.516
129.291
129.128
128.682
128.192
122.329
54.721
54.361
54.000
53.640
53.279
38.435
38.176
35.405
33.101
33.007
32.819
32.725
31.592
30.320
30.273
30.211
30.145
30.034
28.644
28.471
25.457
t-Bu
t-Bu
P
t-Bu
Ph
P
150
100
50
ppm (t1)

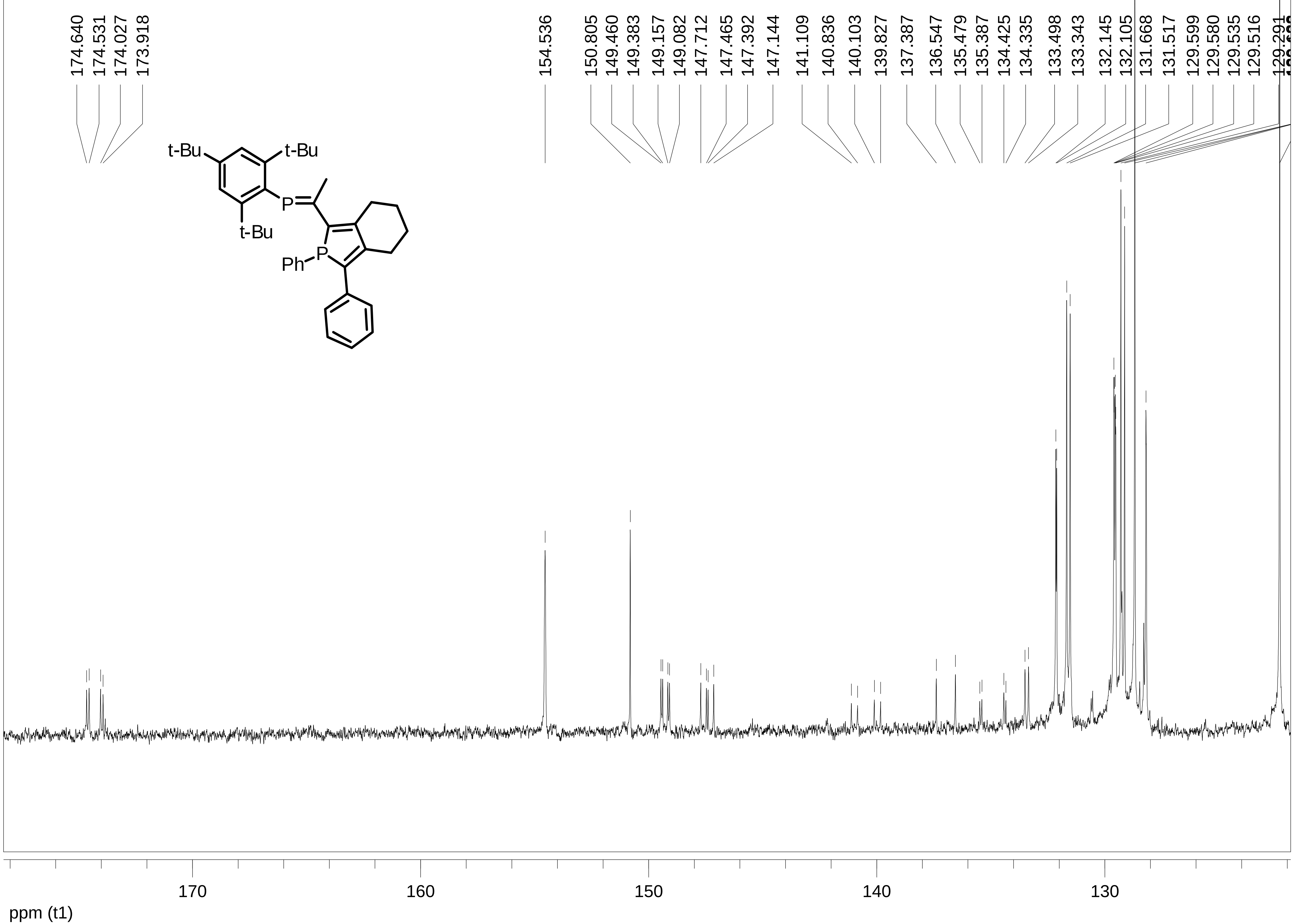

174.640
174.531
174.027
173.918
154.536
150.805
149.460
149.383
149.157
149.082
147.712
147.465
147.392
147.144
141.109
140.836
140.103
139.827
137.387
136.547
135.479
135.387
134.425
134.335
133.498
133.343
132.145
132.105
131.668
131.517
129.599
129.580
129.535
129.516
129.291
t-Bu
t-Bu
P
t-Bu
Ph
P
170
160
150
140
130
ppm (t1)

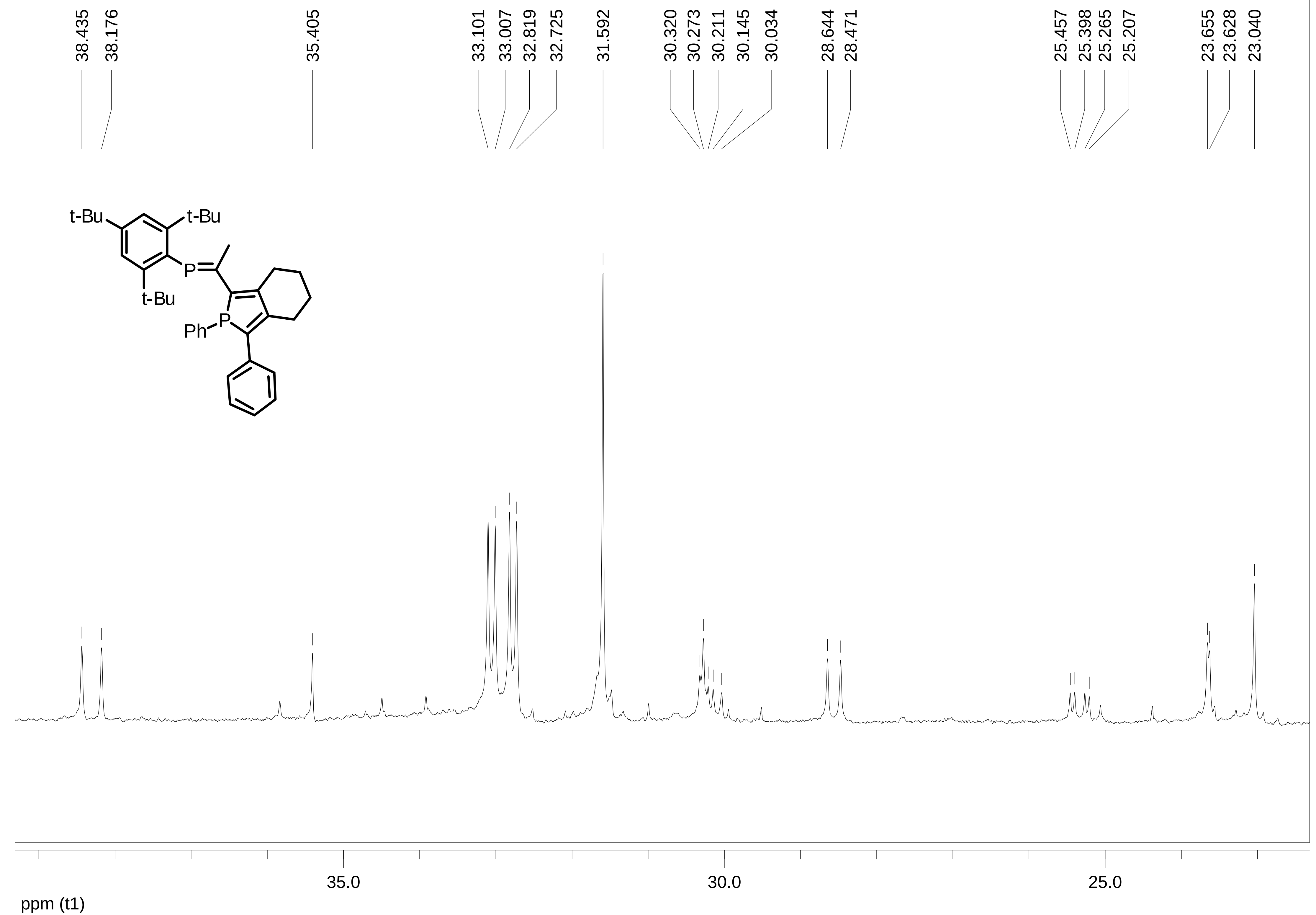
38.435
38.176
35.405
33.101
33.007
32.819
32.725
31.592
30.320
30.273
30.211
30.145
30.034
28.644
28.471
25.457
25.398
25.265
25.207
23.655
23.628
23.040
t-Bu
t-Bu
t-Bu
P
Ph
P
35.0
30.0
25.0
ppm (t1)

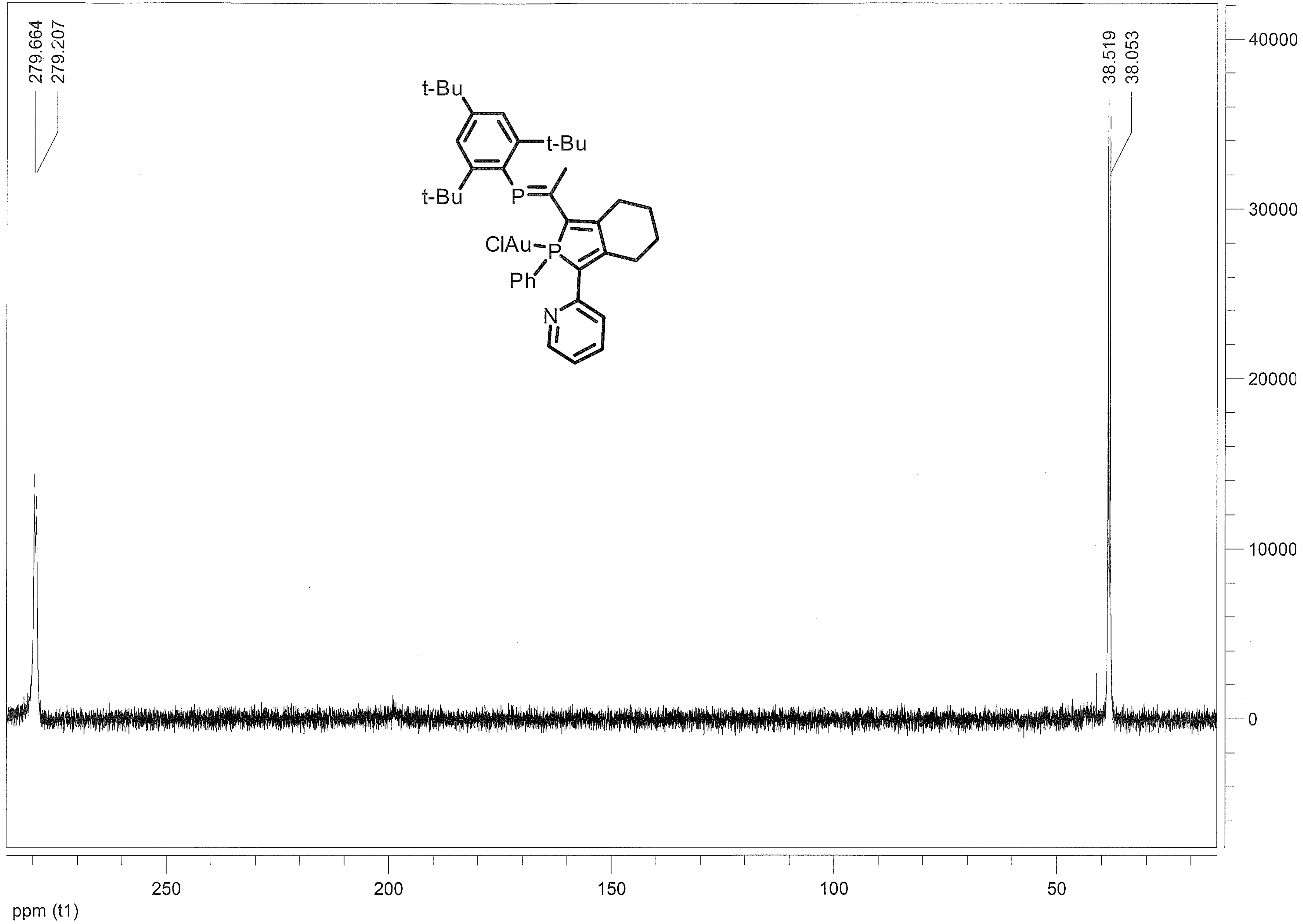
279.664
279.207
38.519
38.053
t-Bu
t-Bu
t-Bu
P
ClAu
P
Ph
N
40000
30000
20000
10000
0
250
200
150
100
50
ppm (t1)

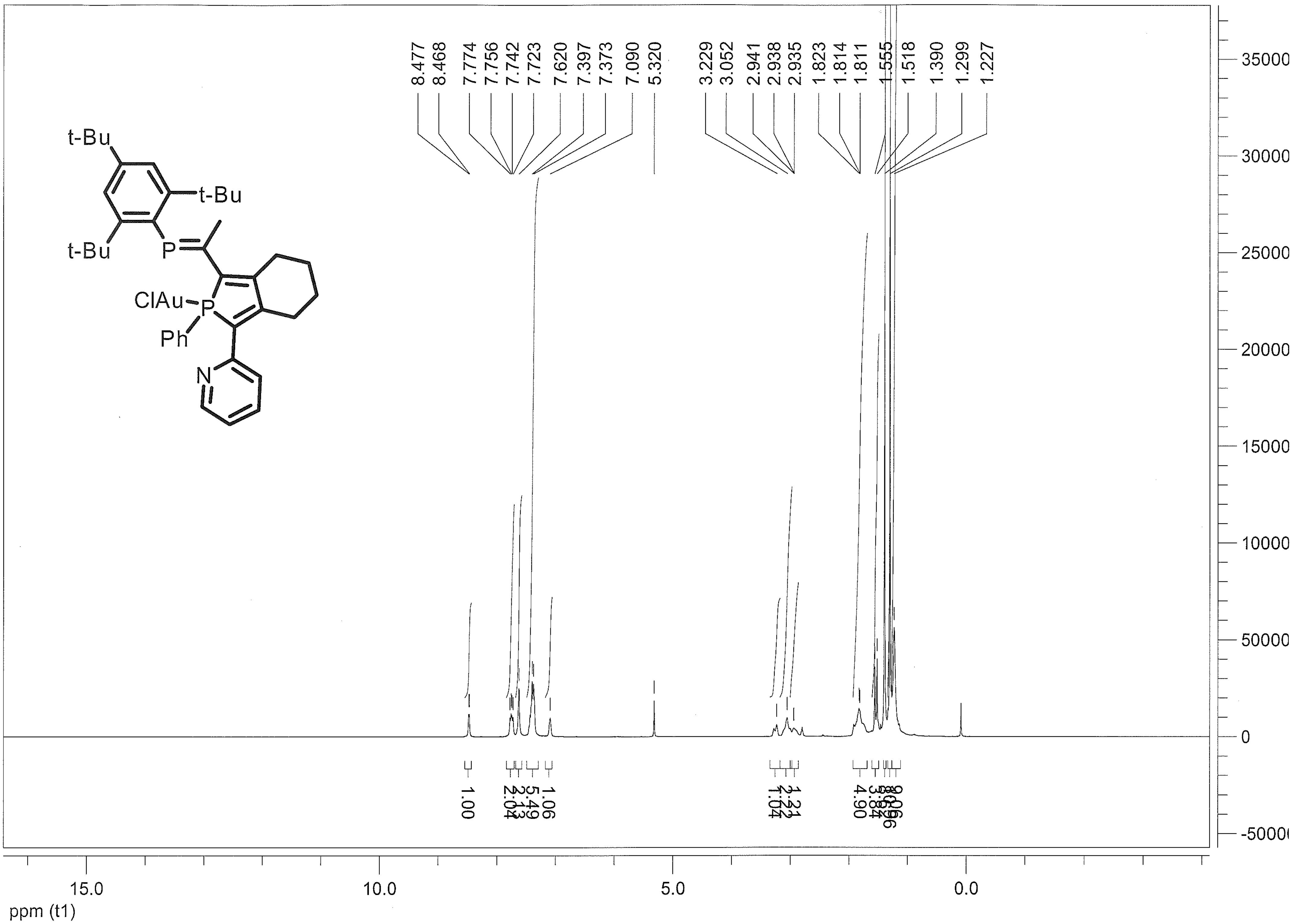

t-Bu
t-Bu
t-Bu
P
ClAu
P
Ph
N
8.477
8.468
7.774
7.756
7.742
7.723
7.620
7.397
7.373
7.090
5.320
3.229
3.052
2.941
2.938
2.935
1.823
1.814
1.811
1.555
1.518
1.390
1.299
1.227
1.00
2.04
2.13
5.49
1.06
1.04
1.22
1.21
4.90
3.84
ppm (t1)
15.0
10.0
5.0
0.0
35000
30000
25000
20000
15000
10000
50000
0

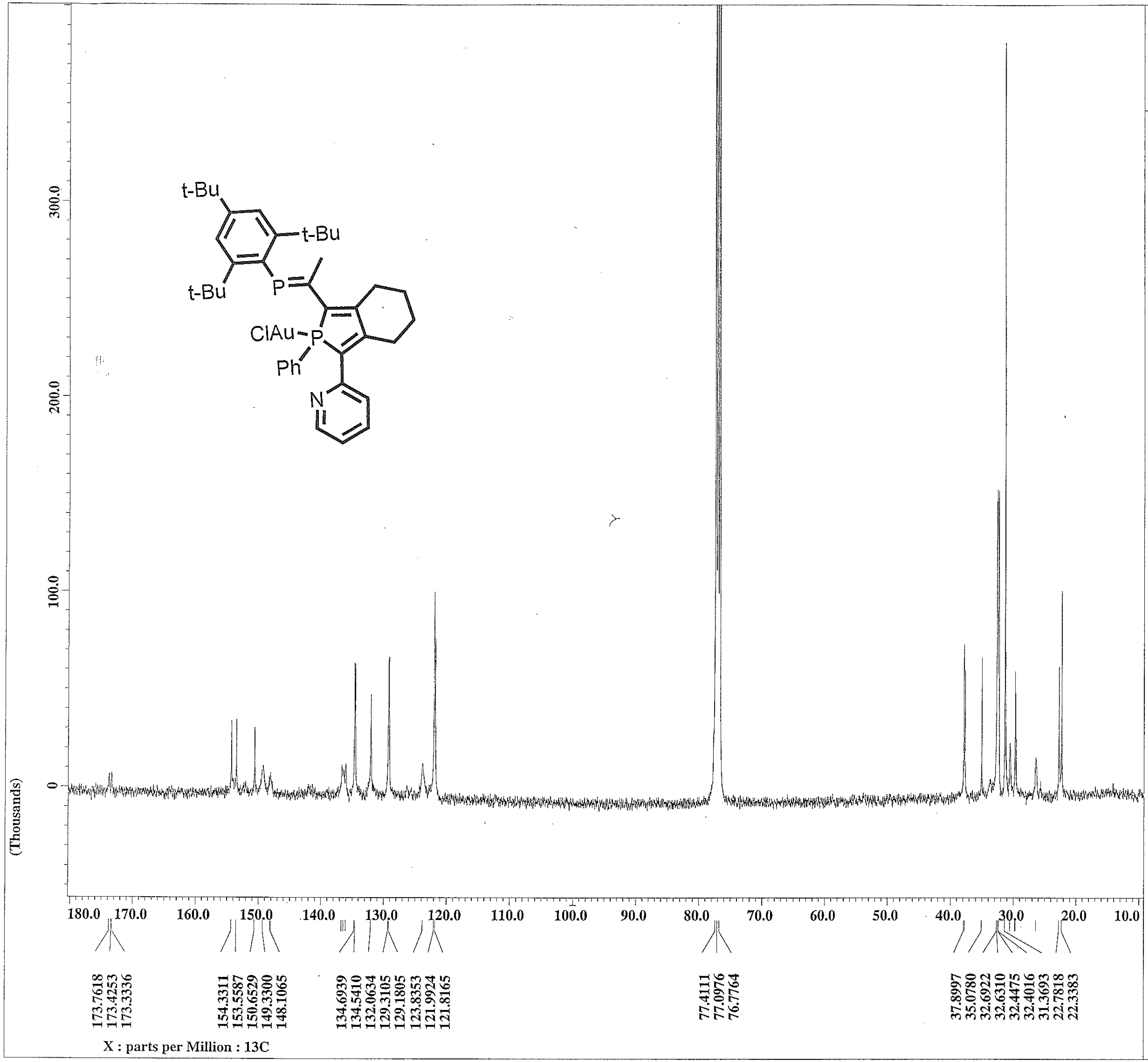

Filename	= EO_1_AuCl-4.jdf
Author	= fotomol
Experiment	= single_pulse_dec
Sample_id	= S#637595
Solvent	= CHLOROFORM-D
Creation_time	= 19-NOV-2012 09:30:19
Revision_time	= 26-NOV-2012 08:26:47
Current_time	= 26-NOV-2012 08:27:13
Content	= Single Pulse with Bro
Data_format	= 1D COMPLEX
Dim_size	= 32768
Dim_title	= 13C
Dim_units	= [ppm]
Dimensions	= X
Site	= Eclipse+ 400
Spectrometer	= DELTA_NMR
Field_strength	= 9.389766[T] (400[MHz]
X_acq_duration	= 1.3008896[s]
X_domain	= 13C
X_freq	= 100.52530333[MHz]
X_offset	= 100[ppm]
X_points	= 32768
X_prescans	= 4
X_resolution	= 0.76870474[Hz]
X_sweep	= 25.18891688[kHz]
Irr_domain	= 1H
Irr_freq	= 399.78219838[MHz]
Irr_offset	= 5[ppm]
Clipped	= TRUE
Mod_return	= 1
Scans	= 100000
Total_scans	= 100000
X_90_width	= 9.7[us]
X_acq_time	= 1.3008896[s]
X_angle	= 30[deg]
X_pulse	= 3.23333333[us]
Initial_wait	= 1[s]
Phase_preset	= 3[us]
Recvr_gain	= 29
Relaxation_delay	= 1[s]
Temp_get	= 24.5[dC]
Unblank_time	= 2[us]

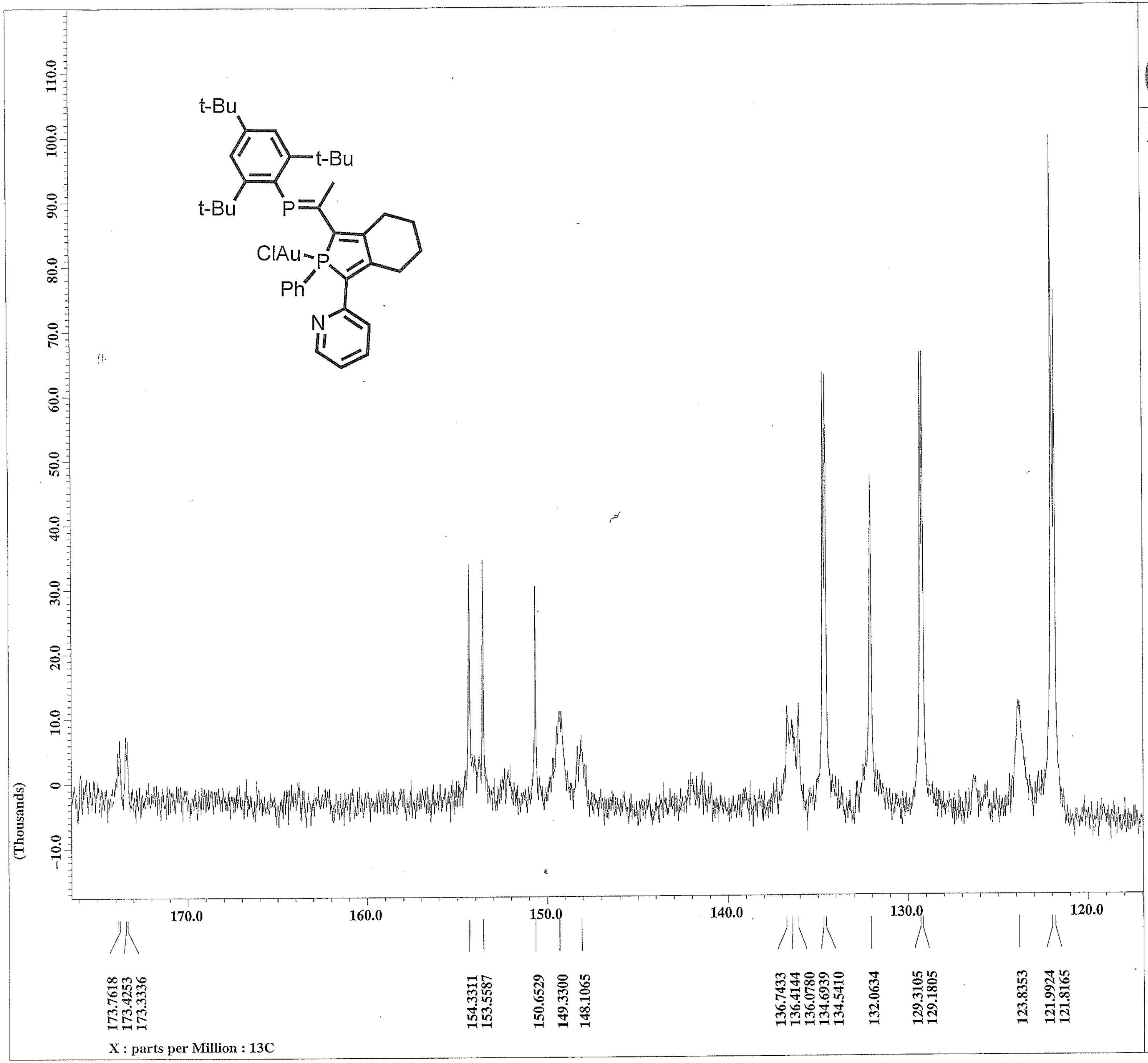

Filename	=	EO_1_AuCl-4.jdf
Author	=	fotomol
Experiment	=	single_pulse_dec
Sample_id	=	S#637595
Solvent	=	CHLOROFORM-D
Creation_time	=	19-NOV-2012 09:30:19
Revision_time	=	26-NOV-2012 08:26:47
Current_time	=	26-NOV-2012 08:27:32
Content	=	Single Pulse with Bro
Data_format	=	1D COMPLEX
Dim_size	=	32768
Dim_title	=	13C
Dim_units	=	[ppm]
Dimensions	=	X
Site	=	Eclipse+ 400
Spectrometer	=	DELTA_NMR
Field_strength	=	9.389766[T] (400[MHz]
X_acq_duration	=	1.3008896[s]
X_domain	=	13C
X_freq	=	100.52530333[MHz]
X_offset	=	100[ppm]
X_points	=	32768
X_prescans	=	4
X_resolution	=	0.76870474[Hz]
X_sweep	=	25.18891688[kHz]
Irr_domain	=	1H
Irr_freq	=	399.78219838[MHz]
Irr_offset	=	5[ppm]
Clipped	=	TRUE
Mod_return	=	1
Scans	=	100000
Total_scans	=	100000
X_90_width	=	9.7[us]
X_acq_time	=	1.3008896[s]
X_angle	=	30[deg]
X_pulse	=	3.23333333[us]
Initial_wait	=	1[s]
Phase_preset	=	3[us]
Recvr_gain	=	29
Relaxation_delay	=	1[s]
Temp_get	=	24.5[dC]
Unblank_time	=	2[us]

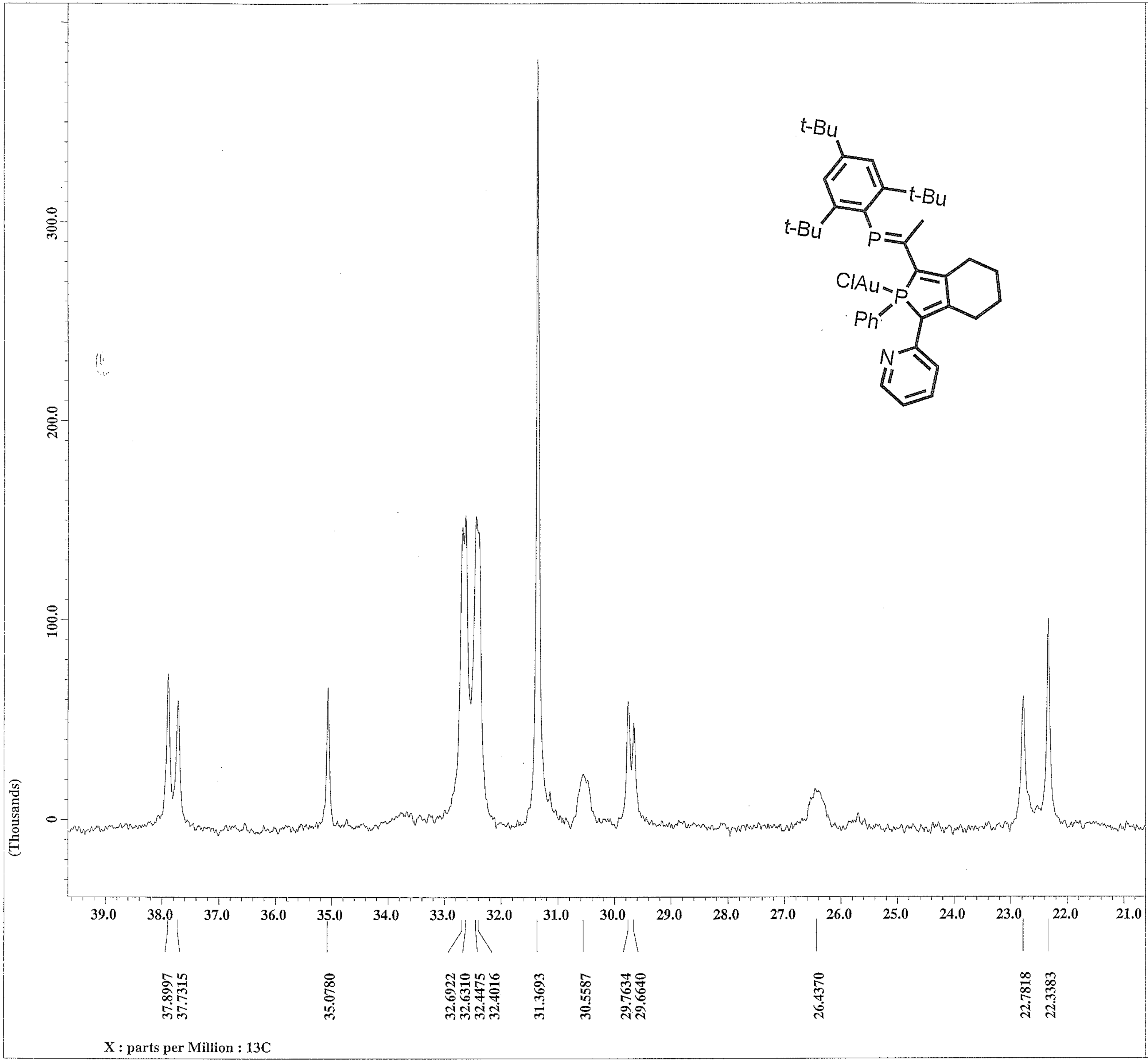

JEOL

Filename	=	EO_1_AuCl-4.jdf
Author	=	fotomol
Experiment	=	single_pulse_dec
Sample_id	=	S#637595
Solvent	=	CHLOROFORM-D
Creation_time	=	19-NOV-2012 09:30:19
Revision_time	=	26-NOV-2012 08:26:47
Current_time	=	26-NOV-2012 08:28:13
Content	=	Single Pulse with Bro
Data_format	=	1D COMPLEX
Dim_size	=	32768
Dim_title	=	13C
Dim_units	=	[ppm]
Dimensions	=	X
Site	=	Eclipse+ 400
Spectrometer	=	DELTA_NMR
Field_strength	=	9.389766[T] (400[MHz]
X_acq_duration	=	1.3008896[s]
X_domain	=	13C
X_freq	=	100.52530333[MHz]
X_offset	=	100[ppm]
X_points	=	32768
X_prescans	=	4
X_resolution	=	0.76870474[Hz]
X_sweep	=	25.18891688[kHz]
Irr_domain	=	1H
Irr_freq	=	399.78219838[MHz]
Irr_offset	=	5[ppm]
Clipped	=	TRUE
Mod_return	=	1
Scans	=	100000
Total_scans	=	100000
X_90_width	=	9.7[us]
X_acq_time	=	1.3008896[s]
X_angle	=	30[deg]
X_pulse	=	3.23333333[us]
Initial_wait	=	1[s]
Phase_preset	=	3[us]
Recvr_gain	=	29
Relaxation_delay	=	1[s]
Temp_get	=	24.5[dC]
Unblank_time	=	2[us]

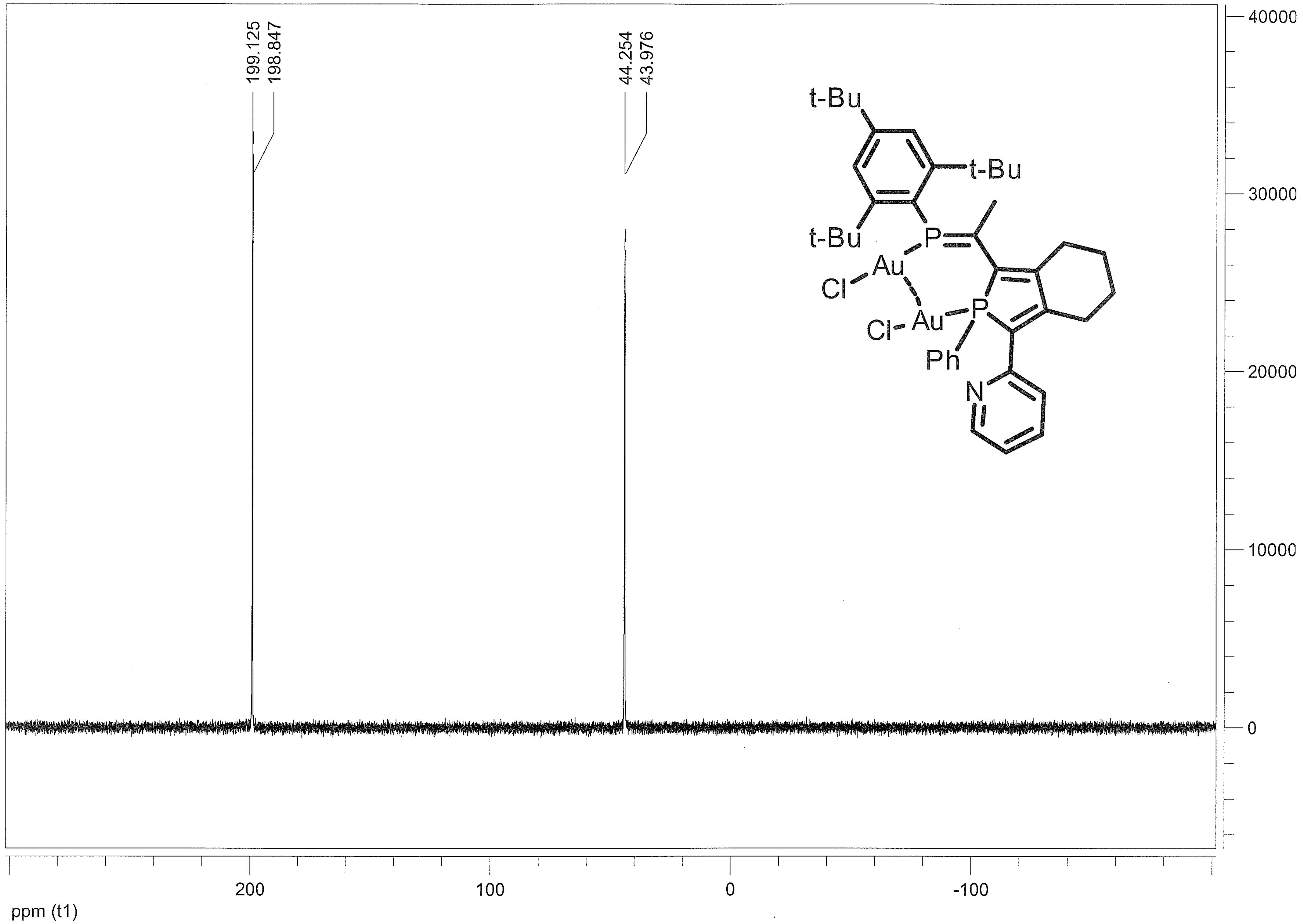

199.125
198.847
44.254
43.976
t-Bu
t-Bu
t-Bu
P
Au
Cl
Au
Cl
P
Ph
N
40000
30000
20000
10000
0
200
100
0
-100
ppm (t1)

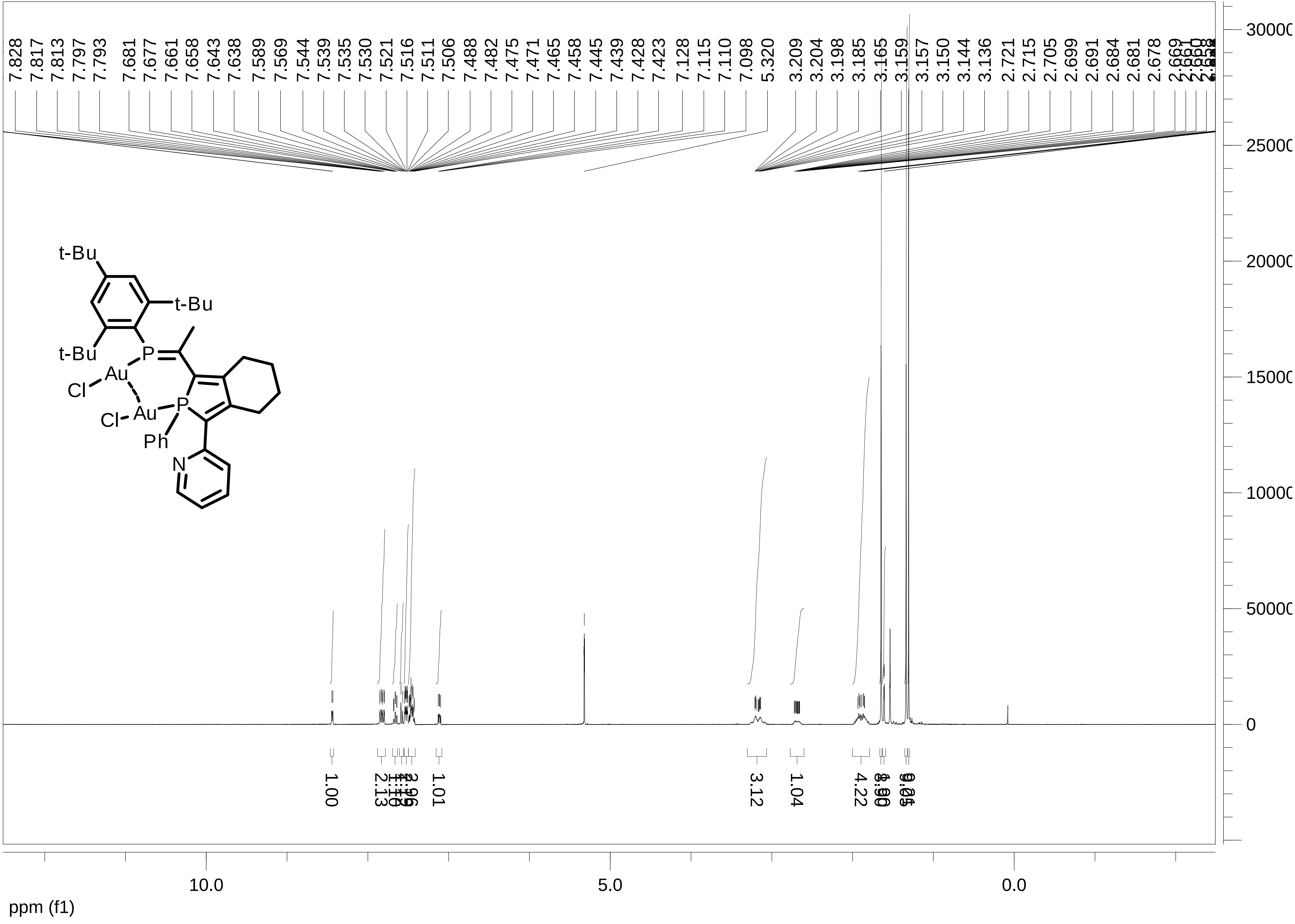

7.828
7.817
7.813
7.797
7.793
7.681
7.677
7.661
7.658
7.643
7.638
7.589
7.569
7.544
7.539
7.535
7.530
7.521
7.516
7.511
7.506
7.488
7.482
7.475
7.471
7.465
7.458
7.445
7.439
7.428
7.423
7.128
7.115
7.110
7.098
5.320
3.209
3.204
3.198
3.185
3.165
3.159
3.157
3.150
3.144
3.136
2.721
2.715
2.705
2.699
2.691
2.684
2.681
2.678
2.669
t-Bu
t-Bu
t-Bu
P
Au
Cl
Cl
Au
P
Ph
N
1.00
2.13
1.01
3.12
1.04
4.22
10.0
5.0
0.0
ppm (f1)

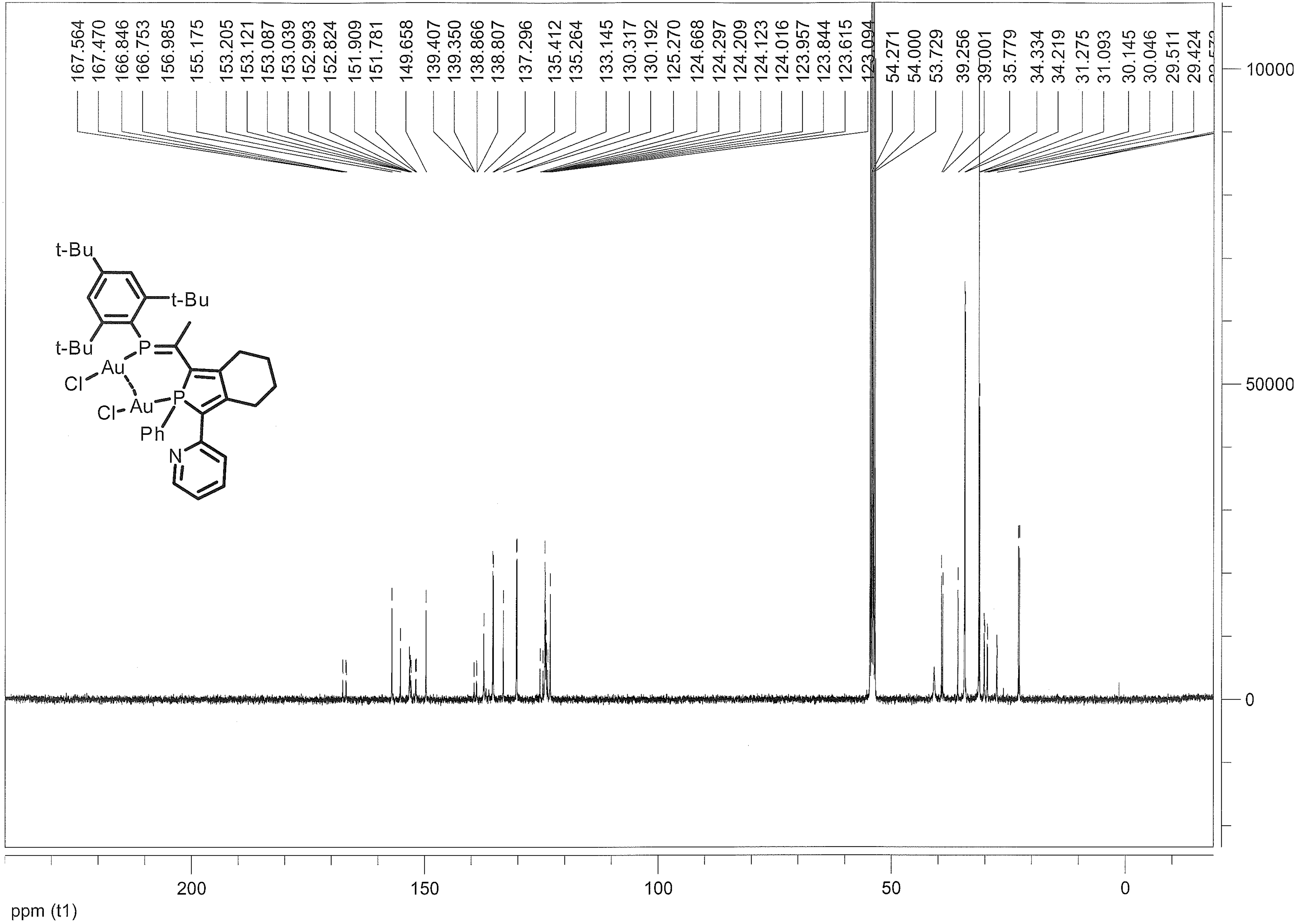

167.564
167.470
166.846
166.753
156.985
155.175
153.205
153.121
153.087
153.039
152.993
152.824
151.909
151.781
149.658
139.407
139.350
138.866
138.807
137.296
135.412
135.264
133.145
130.317
130.192
125.270
124.668
124.297
124.209
124.123
124.016
123.957
123.844
123.615
123.094
54.271
54.000
53.729
39.256
39.001
35.779
34.334
34.219
31.275
31.093
30.145
30.046
29.511
29.424
t-Bu
t-Bu
t-Bu
P
Au
Cl
Au
Cl
P
Ph
N
10000
50000
0
200
150
100
50
0
ppm (t1)

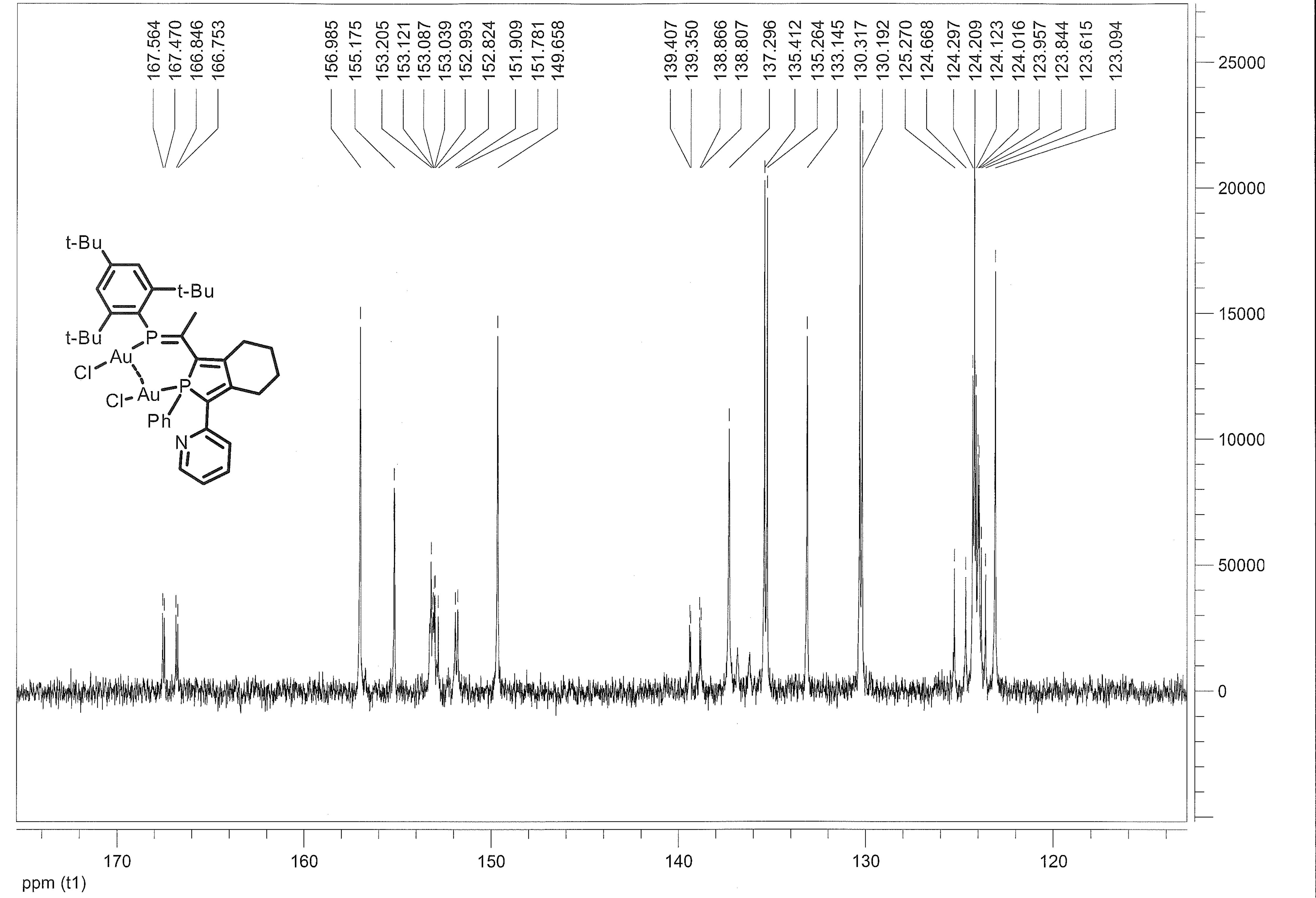

167.564
167.470
166.846
166.753
156.985
155.175
153.205
153.121
153.087
153.039
152.993
152.824
151.909
151.781
149.658
139.407
139.350
138.866
138.807
137.296
135.412
135.264
133.145
130.317
130.192
125.270
124.668
124.297
124.209
124.123
124.016
123.957
123.844
123.615
123.094
25000
20000
15000
10000
50000
0
t-Bu
t-Bu
t-Bu
P
Au
Cl
Cl
Au
P
Ph
N
170
160
150
140
130
120
ppm (t1)

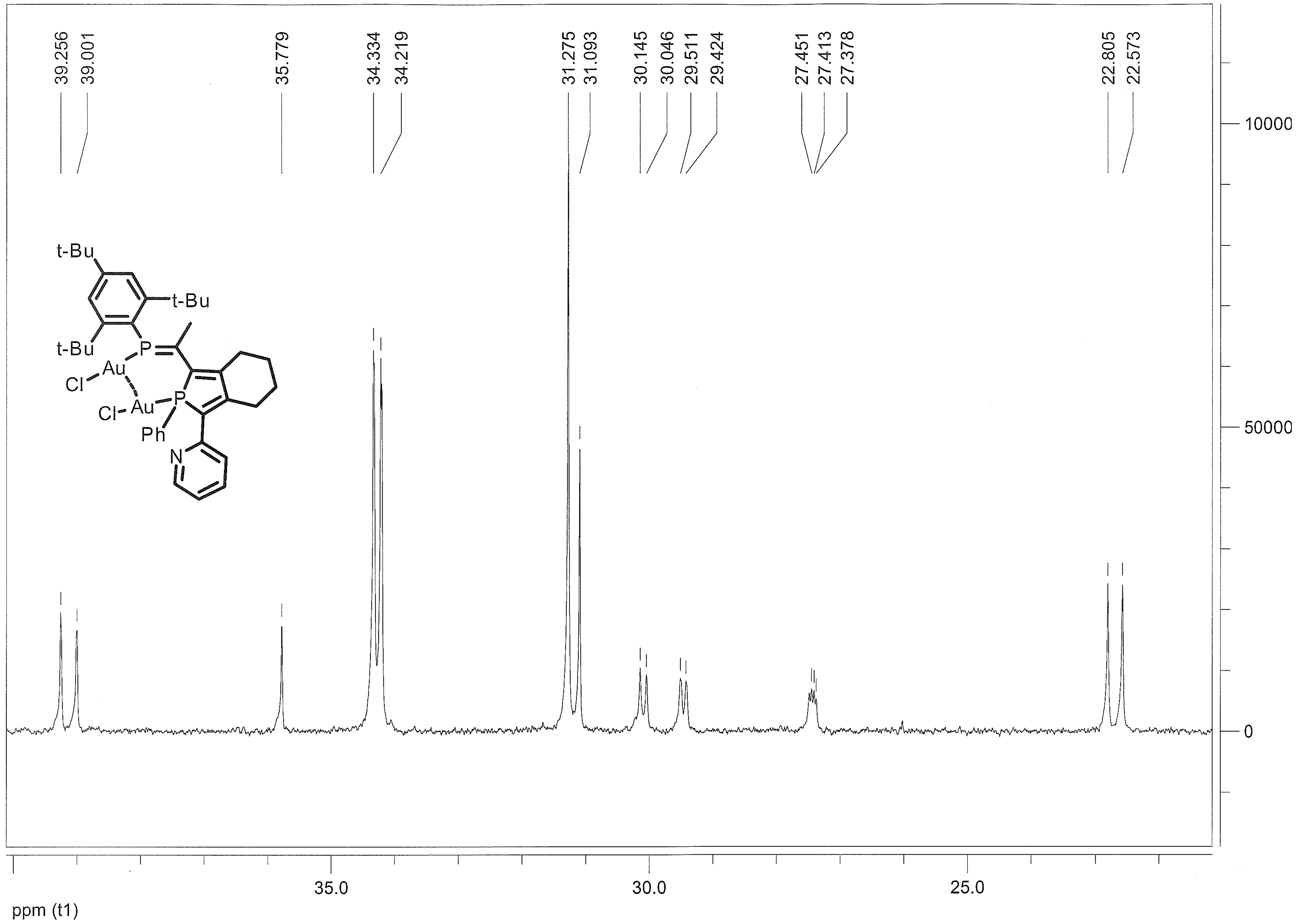

39.256
39.001
35.779
34.334
34.219
31.275
31.093
30.145
30.046
29.511
29.424
27.451
27.413
27.378
22.805
22.573
10000
50000
0
35.0
30.0
25.0
ppm (t1)
t-Bu
t-Bu
t-Bu
P
Au
Cl
Au
Cl
P
Ph
N
